# Closely related yet different: a borylene and its dimer are non-interconvertible but connected through reactivity†
†Electronic supplementary information (ESI) available: Synthesis and characterization of new compounds, NMR spectra, crystallographic details and supplementary structures. CCDC 1582403–1582412. For ESI and crystallographic data in CIF or other electronic format see DOI: 10.1039/c7sc04789d


**DOI:** 10.1039/c7sc04789d

**Published:** 2018-01-04

**Authors:** Dominic Auerhammer, Merle Arrowsmith, Rian D. Dewhurst, Thomas Kupfer, Julian Böhnke, Holger Braunschweig

**Affiliations:** a Institut für Anorganische Chemie , Julius-Maximilians-Universität Würzburg , Am Hubland , 97074 Würzburg , Germany . Email: h.braunschweig@uni-wuerzburg.de; b Institute for Sustainable Chemistry & Catalysis with Boron , Julius-Maximilians-Universität Würzburg , Am Hubland , 97074 Würzburg , Germany

## Abstract

The self-stabilizing, tetrameric cyanoborylene [(cAAC)B(CN)]_4_ (**I**, cAAC = 1-(2,6-diisopropylphenyl)-3,3,5,5-tetramethylpyrrolidin-2-ylidene) and its diborene relative, [(cAAC)(CN)B=B(CN)(cAAC)] (**II**), both react with disulfides and diselenides to yield the corresponding cAAC-supported cyanoboron bis(chalcogenides). Furthermore, reactions of **I** or **II** with elemental sulfur and selenium in various stoichiometries provided access to a variety of cAAC-stabilized cyanoboron–chalcogen heterocycles, including a unique dithiaborirane, a diboraselenirane, 1,3-dichalcogena-2,4-diboretanes, 1,3,4-trichalcogena-2,5-diborolanes and a rare six-membered 1,2,4,5-tetrathia-3,6-diborinane. Stepwise addition reactions and solution stability studies provided insights into the mechanism of these reactions and the subtle differences in reactivity observed between **I** and **II**.

## Abstract 2

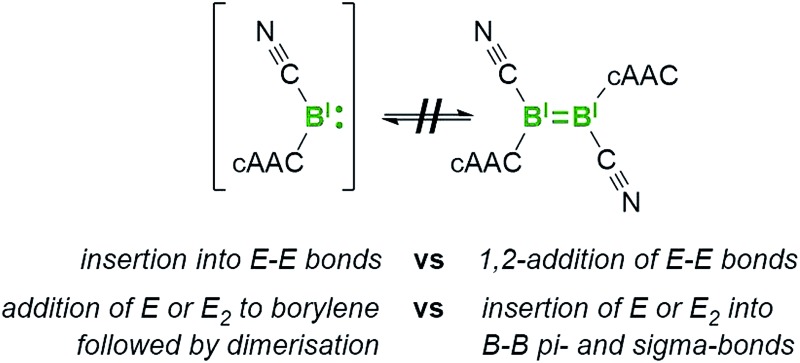
This side-by-side reactivity study of a borylene and a diborene with the same empirical formula demonstrates their non-interconvertibility.

## Introduction

**Fig. 1:**
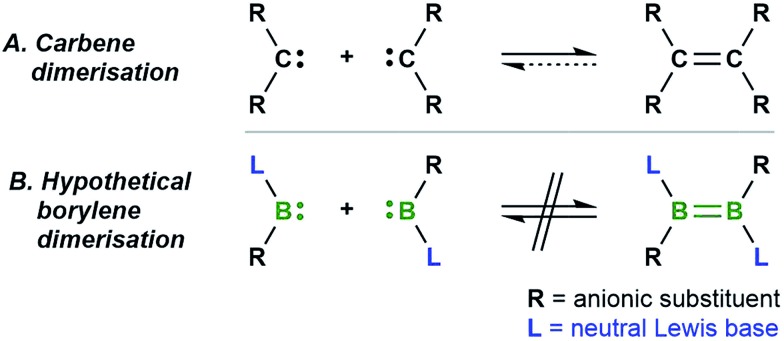
The well-known dimerisation of carbenes (**A**) and the currently unknown dimerisation of isoelectronic [:BRL] borylenes (**B**).

The dimerisation of carbenes [R_2_C:] to form alkenes (Fig. 1A) is a fundamental reaction pattern in carbene chemistry^1^ and the basis of the so-called Wanzlick equilibrium.^2^ A similar dimerisation process has also been observed with heavier analogues of carbenes.^3^ However, despite the existence of isoelectronic group 13 analogues of carbenes – borylenes of the form [LRB:] (R = anionic substituent, L = neutral Lewis basic substituent) – no carbene-analogous dimerisation (Fig. 1B) has been observed for group 13 species.

Singly base-stabilised [LRB:] borylenes belong to the family of monovalent boron species discovered and recently reviewed by Bertrand *et al.*^4^ Perhaps the most definitive and well-defined example of this class of compounds is the linear borylene [(cAAC^Cy^)BN(SiMe_3_)_2_] (cAAC^Cy^ = 2-(2,6-diisopropylphenyl)-3,3-dimethyl-2-azaspiro[4.5]decan-1-ylidene^5^) reported by Stephan and Bertrand.^6^ Over the past few years, however, our group has demonstrated that a number of doubly base-stabilized borylene species ([LL′RB:]) can act as synthons for [LRB:] borylenes, either through photodecarbonylation^7^,^8^ or the base-mediated deaggregation of tetrameric cyanoborylene [(cAAC)B(CN)]_4_ (**I**, cAAC = 1-(2,6-diisopropylphenyl)-3,3,5,5-tetramethylpyrrolidin-2-ylidene).^9^

The generation of [LRB:] borylenes, either stable or transient, has allowed the study of coordination chemistry at monovalent boron centers through binding of new Lewis bases, in addition to intramolecular C–H and C–C bond activations in the absence of a suitable base.^6^–^9^ The currently unrealised possibility of dimerising [LRB:] species would provide a new synthetic route to doubly base-stabilised diborenes of the form [LRB=BRL], which still suffer from considerable synthetic restrictions.^10^

**Scheme 1:**
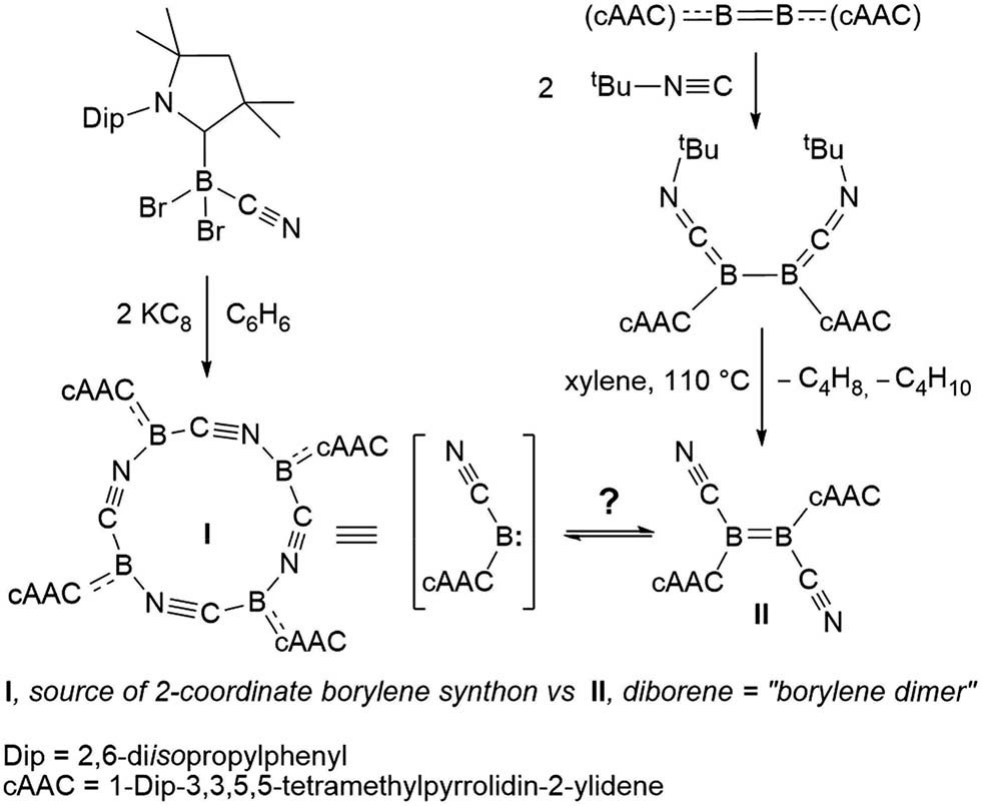
Syntheses of two boron(i) compounds of the same empirical formula, cyanoborylene **I** and dicyanodiborene **II**.

Our recent synthesis of the aforementioned tetraborylene macrocycle [(cAAC)B(CN)]_4_ (**I**; Scheme 1, left)^9^ and the doubly base-stabilised dicyanodiborene [(cAAC)(NC)B=B(CN)(cAAC)] (**II**; Scheme 1, right)^11^ presented an interesting juxtaposition of related molecules. Macrocycle **I** is a tetramer (and synthetic equivalent) of the borylene [(cAAC)(CN)B:], while diborene **II** is a formal dimer of the same borylene. Having failed thus far to realise the interconversion of **I** and **II** through photolysis and/or heating, we were eager to test their respective reactivity with suitable reagents in order to define any differences or similarities, and hopefully gain definitive proof of their ability (or inability) to interconvert. Herein we present the reactivity of **I** and **II** with elemental chalcogens and dichalcogenides, based on our recently-reported reactions of these chalcogen reagents with B–B multiply-bonded species.^12^,^13^ Our results indicate that, although extensive similarities exist between borylene **I** and its formal dimer **II**, and they may therefore be viewed as close relatives, subtle differences in reactivity confirm that no Wanzlick-type equilibrium exists between the two.

## Results and discussion

### Synthetic studies and characterisation

**Scheme 2:**
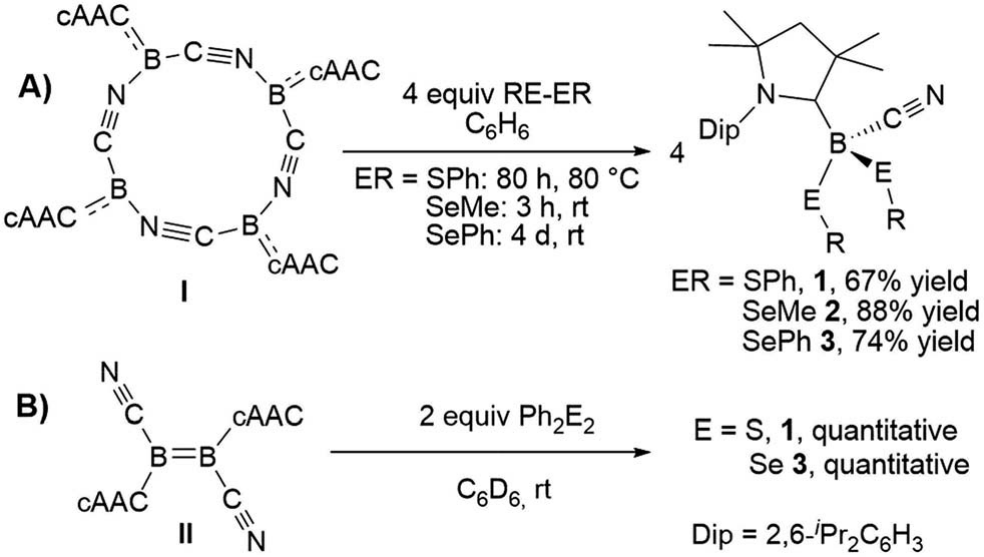
Reactivity of **I** and **II** with dichalcogenides.

The reaction of tetrameric borylene **I** with four equivalents of diorganyldichalcogenides, E_2_R_2_ (E = S, Se, R = Ph; E = Se, R = Me) in benzene proceeded selectively to the corresponding cAAC-supported cyanoboron dichalcogenides, [(cAAC)B(CN)(ER)_2_] (ER = SPh **1**, SeMe **2**, SePh **3**), which were isolated in moderate to good yields (Scheme 2A). Whereas the reaction with diphenyldisulfide required prolonged heating at 80 °C to proceed to completion, reactions with diselenides proceeded at room temperature, the reaction with Se_2_Me_2_ being significantly faster than that with Se_2_Ph_2_. Compounds **1–3** showed ^11^B NMR resonances typical of sp^3^ hybridized boranes, with the bis(selenides) **2** (–18.4 ppm) and **3** (–14.4 and –15.8 ppm) exhibiting shifts significantly downfield from the bis(sulfide) **1** (–9.6 ppm). Furthermore, at room temperature the bis(phenylselenide) **3** displayed highly broadened ^1^H NMR ligand resonances as well as two distinct, very broad ^11^B NMR resonances (–14.4 and –15.8 ppm), which coalesced upon heating to 70 °C, indicating hindered rotation, presumably owing to steric interactions between the bulky cAAC substituents and the large phenylselenide ligands.

NMR spectroscopic data of reaction mixtures of diborene **II** and Ph_2_S_2_ or Ph_2_Se_2_ showed the monoboron bis(chalcogenides) **1** and **3** to be sole products of these reactions, independent of the stoichiometry used (Scheme 2B). These reactions proceeded much faster and at lower temperatures than the corresponding additions of dichalcogenides to the tetrameric borylene **I**. Although no intermediates were observed under these reaction conditions, it is likely that the formation of **1** and **3** from **II** proceeds *via* the successive 1,2-addition of the E–E σ-bond first across the B=B double bond and, subsequently, across the remaining B–B single bond. Although both compounds **I** and **II** reacted with Ph_2_Te_2_ at high temperatures, these reactions were rather unselective and did not yield any tractable products.

**Fig. 2:**
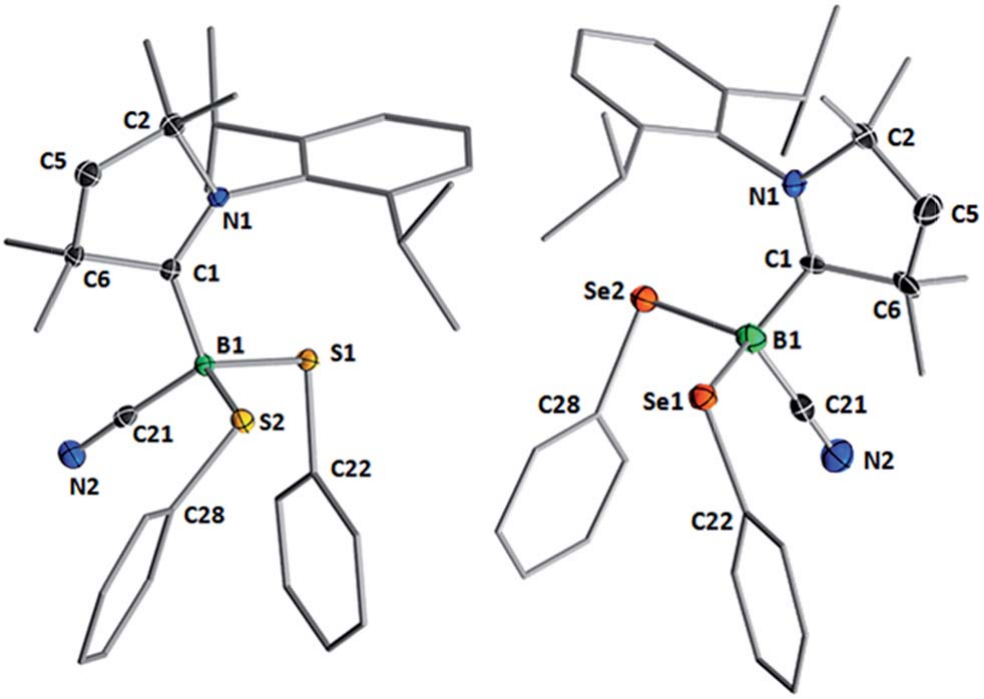
Crystallographically determined solid-state structures of **1** (left) and **3** (right). Atomic displacement ellipsoids depicted at 50% probability level. Hydrogen atoms and atomic displacement ellipsoids of peripheral substituents omitted for clarity. Selected bond lengths (Å) and angles (°) for **1**: B1–C1 1.6297(19), B1–C21 1.584(2), B1–S1 1.9248(15), B1–S2 1.9578(16); B1–S1–C22 100.21(6), B1–S2–C28 112.33(6). For **3**: N1–C1 1.295(7), B1–C1 1.597(9), B1–C21 1.580(9), C21–N2 1.139(7), B1–Se1 2.109(7), B1–Se2 2.056(8); B1–Se1–C22 107.1(3), B1–Se2–C28 97.8(3).

Compounds **1–3** readily crystallized from THF at room temperature, **1** as a colorless crystalline solid, and **2** and **3** as yellow crystals.‡
‡Compound **2** repeatedly crystallized as extremely thin, overlapping yellow plates, which could not be separated. X-ray crystallographic analysis of these provided proof of connectivity but data were of insufficient quality for further structural discussions (see ESI, Fig. S44†).
Fig. 2 shows the crystallographically determined structures of the bis(phenylchalcogenides) **1** and **3**, which crystallize in isomorphous unit cells. The B–C_cAAC_ bond length in disulfide **1** (1.6297(19) Å) is slightly elongated compared to that in diselenide **3** (1.597(9) Å). The B–C_CN_ bond lengths (**1**: 1.584(2); **3**: 1.580(9)) are similar to those found in other cAAC-supported cyanoboranes (1.574(5)–1.589(3) Å).^9^,^14^ While the B–E bond lengths (B–S: 1.9248(15), 1.9578(16) Å; B–Se: 2.109(7), 2.056(8) Å) are within the typical range for Lewis-base-stabilized boron organochalcogenides, such as Marder and Norman's pyridine and phosphine adducts of 2,2′-bibenzo[*d*][1,3,2]dithiaboroles (B–S: 1.899(5)–1.930(2) Å)^15^ or Wrackmeyer's base-stabilized boron 1,2-diselenato-*ortho*-carboranes (B–Se: 2.031(6)–2.065(5) Å).^16^ While structurally unspectacular, species **1–3** represent the first examples of boron chalcogenides synthesized by the atom efficient insertion of a borylene into the E–E bond of a diorganodichalcogenide.

**Scheme 3:**
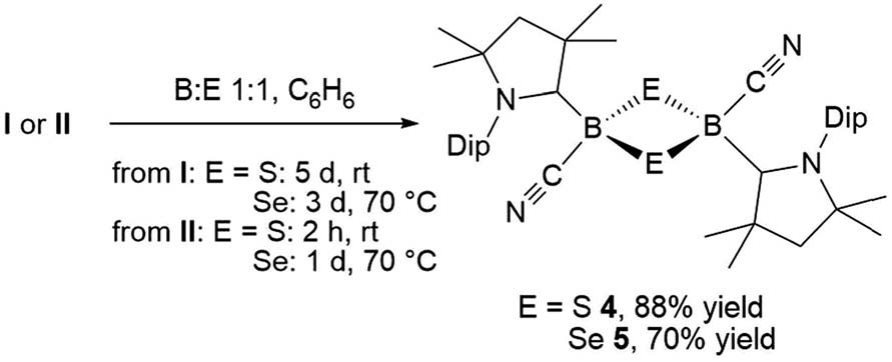
Synthesis of 1,3-dichalcogena-2,4-diboretanes from **I** and **II**.

Stirring of a dark red suspension of tetrameric borylene **I** with elemental sulfur in a 1 : 1 boron-to-sulfur ratio in benzene for 5 d at room temperature resulted in a yellow suspension, which upon filtration and slow evaporation yielded compound **4** as a yellow crystalline solid (Scheme 3). Compound **4** displayed a ^11^B NMR singlet at –17.9 ppm and a single set of ^1^H NMR resonances for the cAAC ligand, suggesting a highly symmetrical species. Despite repeated recrystallization attempts, crystalline samples of **4** always contained 5–10% of another species displaying a higher field ^11^B NMR resonance at –9 ppm (*vide infra*). The analogous reaction with elemental selenium at 60 °C for 3 d similarly provided compound **5** as an orange crystalline solid in 70% yield. Compound **5** was isolated as a single species with an ^11^B NMR singlet at –33.5 ppm, shifted *ca.* 25 ppm upfield from that of **4**, and a highly shielded, broad ^77^Se{^1^H} NMR resonance at –143.1 ppm. In solution at room temperature **5** partially isomerized to a second species presenting an ^11^B NMR shift at –31.8 ppm and slightly shifted ^1^H and ^13^C NMR resonances. High resolution mass spectrometry experiments performed on **4** and **5** provided molecular masses consistent with dimeric compounds of the formula [(cAAC)B(CN)E]_2_ (E = S **4**, Se **5**).

**Fig. 3:**
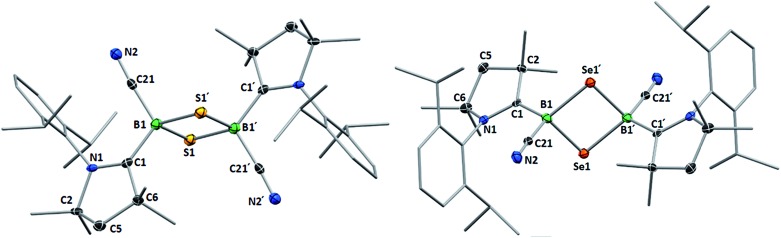
Crystallographically determined solid-state structures of **4** (left) and **5** (right). Atomic displacement ellipsoids depicted at 50% probability level. Hydrogen atoms and atomic displacement ellipsoids of peripheral substituents omitted for clarity. Selected bond lengths (Å) and angles (°) for **4**: N1–C1 1.310(3), B1–C1 1.639(4), B1–C21 1.597(4), C21–N2 1.146(3), B1–S1 1.939(3), B1–S1′ 1.940(3); B1–S1–B1′ 85.21(12), S1–B1–S1′ 94.80(12). For **5**: N1–C1 1.321(4), B1–C1 1.606(5), B1–C21 1.577(6), C21–N2 1.157(4), B1–Se1 2.069(4), B1–Se1′ 2.100(4); B1–Se1–B1′ 85.13(17), Se1–B1–Se1′ 94.87(17).

This was confirmed by X-ray crystallographic analyses, the results of which are displayed in Fig. 3. Compounds **4** and **5** crystallized in near-identical triclinic unit cells as centrosymmetric species presenting planar 1,3-dithia- and 1,3-diselena-2,4-diboretane cores, respectively. The B_2_S_2_ ring in **4** is an approximate square with four almost identical B–S bonds (1.939(3) and 1.940(3) Å) and near-perpendicular B–S–B and S–B–S angles (85.21(12) and 94.80(12)°, respectively), whereas the planar B_2_Se_2_ ring in **5** displays two slightly different B–Se bond lengths (2.069(4) and 2.100(4) Å) as well as near-perpendicular B–Se–B and Se–B–Se angles (85.13(17) and 94.87(17)°, respectively). There are only a couple of structurally characterized 1,3,2,4-dichalcogenadiboretanes in the literature, all displaying sp^2^ hybridized boron centers stabilized by π-donating amino substituents.^17^,^18^ Due to the lower coordination number at boron, the B–E bonds in Nöth's 2,2,6,6-tetramethylpiperidine-supported B_2_S_2_ and B_2_Se_2_ heterocycles are *ca.* 0.08–0.10 Å shorter than in **4** and **5**, while the B–E–B and E–B–E angles are *ca.* 3° narrower and wider, respectively.^18^

For both compounds the plane consisting of the cyanoboron moiety and the π-framework of the cAAC ligand forms a *ca.* 80° angle with the B_2_E_2_ plane, with the respective ligands lying in *trans*-conformation with respect to the B_2_E_2_ core. While the solid-state structure of **5** shows a single conformational isomer, it remains unclear whether the observation of two isomers in solution is the result of a 180° rotation of one of the cAAC ligands around the B–C_cAAC_ bond or the existence of a *cis*-conformer, in which both cyano ligands occupy a *cis*-arrangement with respect to the heterocyclic core. The B–C_cAAC_ bond lengths (**4**: 1.639(4), **5**: 1.606(5) Å) suggest that the cAAC ligand functions as a pure σ-donor ligand to the sp^3^ borane, unlike in borylene **I**, where a significant contribution of π-backbonding from the electron-rich B(i) center shortens the bond (*ca.* 1.47 Å). It is noteworthy that both the B–C_cAAC_ and B–C_CN_ bond lengths are significantly longer in **4** than in **5** (B–C_CN_: **4** 1.597(4), **5** 1.577(6) Å).

**Scheme 4:**
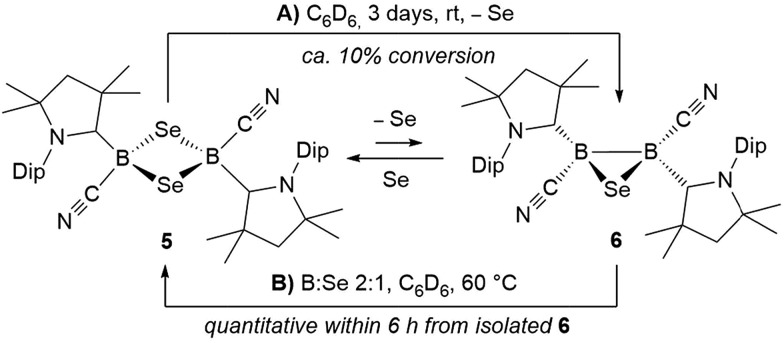
Reversible ring contraction of 1,3-diselena-2,4-diboretane **5**.

**Fig. 4:**
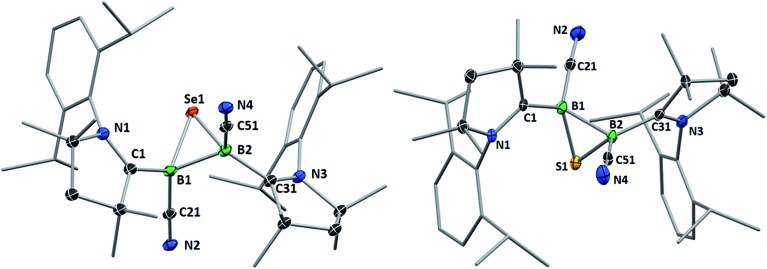
Crystallographically determined solid-state structures of the major one (88%) of the two overlapping disordered molecules of **6** in the asymmetric unit (left)§ and **7** (right). Atomic displacement ellipsoids depicted at 50% probability level. Hydrogen atoms and atomic displacement ellipsoids of peripheral substituents omitted for clarity. Selected bond lengths (Å) and angles (°) for **6**: B1–C1 1.585(5), B2–C31 1.599(6), B1–C21 1.597(4), B2–C51 1.571(6), B1–B2 1.757(6), B1–Se1 2.030(4), B2–Se1 2.027(4); B1–Se1–B2 51.31(18), Se1–B1–B2 64.27(18), Se1–B2–B1 64.42(19). For **7**: B1–C1 1.575(6), B2–C31 1.580(6), B1–C21 1.557(6), B2–C51 1.549(7), B1–B2 1.777(6), B1–S1 1.860(5), B2–S1 1.869(5); B1–S1–B2 56.9(2), S1–B1–B2 61.8(2), S1–B2–B1 61.3(2).

**Scheme 5:**
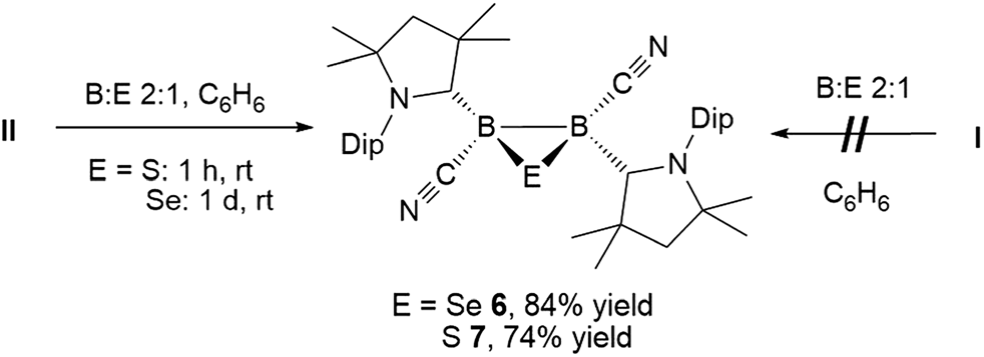
Direct synthesis of 1,2,3-chalcogenadiboriranes from **II** only.

Whereas **4** proved indefinitely stable in solution at room temperature, the ^11^B NMR spectrum of an analytically pure sample of **5** in CD_2_Cl_2_ left at room temperature for 3 d showed the partial disappearance (<10%) of **5** concomitant with the appearance of a new boron-containing species at –22.8 ppm (Scheme 4A). A few crystals of this species could be isolated by recrystallization from diethyl ether and analyzed by mass spectroscopy, revealing a compound of the formula [(cAAC)_2_B_2_(CN)_2_Se] (**6**). X-ray crystallographic analysis of this compound showed that **6** is indeed a bis(cAAC)-stabilized 2,3-dicyano-2,3,1-diboraselenirane (Fig. 4).§
§The solid-state structure of molecule **6** presented a two-fold mirror disorder in a 88 : 12 ratio around the central diboraselenirane core, with the mirror plane running approximately through the plane containing (N1, Se1, N2). The disordered 2,3-dicyano-2,3-diboraselenirane cores of both parts were freely refined. Fig. S45† provides an overlay of the two parts. The structure of **6** is similar to that of the bis(NHC)-stabilized 2,3-dithienyl-2,3,1-diboraselenirane obtained by our group upon reaction of one equivalent of selenium with the corresponding diborene precursor,^13^ and likewise displays a *trans*-arrangement of the cyano and cAAC ligands with respect to the B_2_Se core. Attempts to generate **6** from **I** using a 2 : 1 boron-to-selenium ratio failed, resulting instead in 50% conversion to the diselenadiboretane **5**, with the remaining 50% of **I** left unreacted (Scheme 5). It thus appears that **6** is not accessible directly from borylene **I** but can be generated in solution at room temperature through loss of one selenium atom from **5**, concomitant with B–B bond formation. This was also confirmed by closer inspection of the mass spectrum of compound **5** which showed mass patterns corresponding to **6**.

Conversely, compound **6** was the only product of the reaction of diborene **II** with Se (2 : 1 ratio of boron to selenium) at room temperature in C_6_H_6_, as shown by the appearance of the ^11^B NMR shift around –22 ppm (Scheme 5). Isolated samples of **6** showed asymmetric ^1^H NMR cAAC resonances due to the presence of the chiral centers in B1 and B2. Attempts to detect the ^77^Se NMR resonance of **6** failed due to coupling to the two adjacent quadrupolar boron nuclei. Similarly, the reaction of diborene **II** with S (2 : 1 ratio of boron to sulfur) at room temperature in C_6_H_6_ yielded the corresponding 2,3-dicyano-1,2,3-thiadiborirane **7** (Scheme 5), which presented an intense orange coloration in solution and is, to our knowledge, the first example of a thiadiborirane. The ^11^B NMR shift of **7**, observed at –22.6 ppm, is nearly identical to that of the selenium analogue **6**, suggesting little electronic influence of the chalcogen atom in these B_2_E heterocycles. Both **6** and **7** also displayed a near-identical set of unsymmetrical ^1^H NMR cAAC resonances owing to the presence of the two chiral centers at boron.

**Scheme 6:**
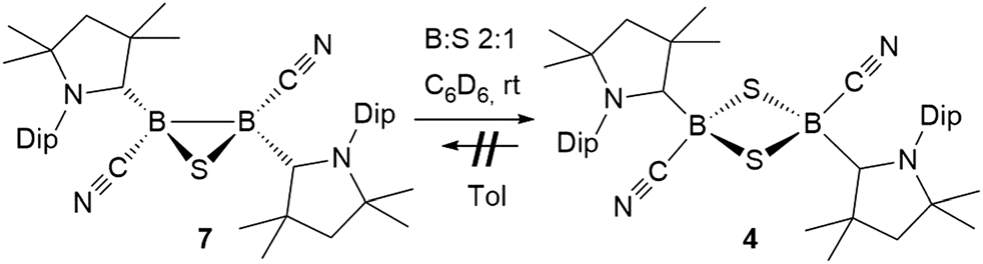
Irreversible ring expansion of 1,2,3-thiadiborirane **7**.

The reaction of **6** with one molar equivalent of selenium heated for 6 hours at 60 °C in benzene resulted in clean conversion to the 1,3-diselena-2,4-diboretane **5**, as indicated by the ^11^B NMR shifts of the two isomers observed at –31.8 and –33.5 ppm (Scheme 4B). Similarly, the addition of one equivalent of elemental sulfur to thiadiborirane **7** cleanly yielded the 1,3-dithia-2,4-diboretane **4** (Scheme 6). But whereas compounds **5** and **6** are in equilibrium in the presence of selenium, with {**5** + Se} favoured at room temperature and **6** favoured at elevated temperature, toluene solutions of **5** showed no evidence of ring contraction to **7** even after prolonged storage at –30 °C. The equilibrium between **5** and **6** could not be further quantified due to the poor solubility of compound **5** and elemental selenium at low temperature in toluene. It does, however, constitute a rare example of fully reversible cleavage of a B–B single bond under extremely mild conditions, and is thereby reminiscent of the reversible insertion of molecular CO into the B–B bond of the borylene borane [(cAAC)H_2_B–B(CO)(cAAC)], which we reported recently.^19^

Single crystals of **7** showed a structure very similar to that of its selenium analogue **6**, displaying the same *trans*-arrangement of the cyano and cAAC ligands with respect to the B_2_S core (Fig. 4). The only notable difference lies in the slightly longer B–B bond (**7** 1.777(6); **6** 1.757(6) Å) enforced by the significantly shorter B–E bonds (**6**: B–S *ca.* 1.86; **7**: B–Se *ca.* 2.03 Å) and the slightly wider B–E–B angle (**6**: 56.9(2); **7**: 51.31(18)°). Unlike the IMe-supported dithienyldiborene^13^ or the manganese-bound borylene [Cp(OC)_2_Mn=B^*t*^Bu(IMe)] (Cp = cyclopentadienyl),^20^ neither compound **I** or **II** showed any reactivity toward elemental tellurium in toluene, even after prolonged heating in benzene at 80 °C.

**Scheme 7:**
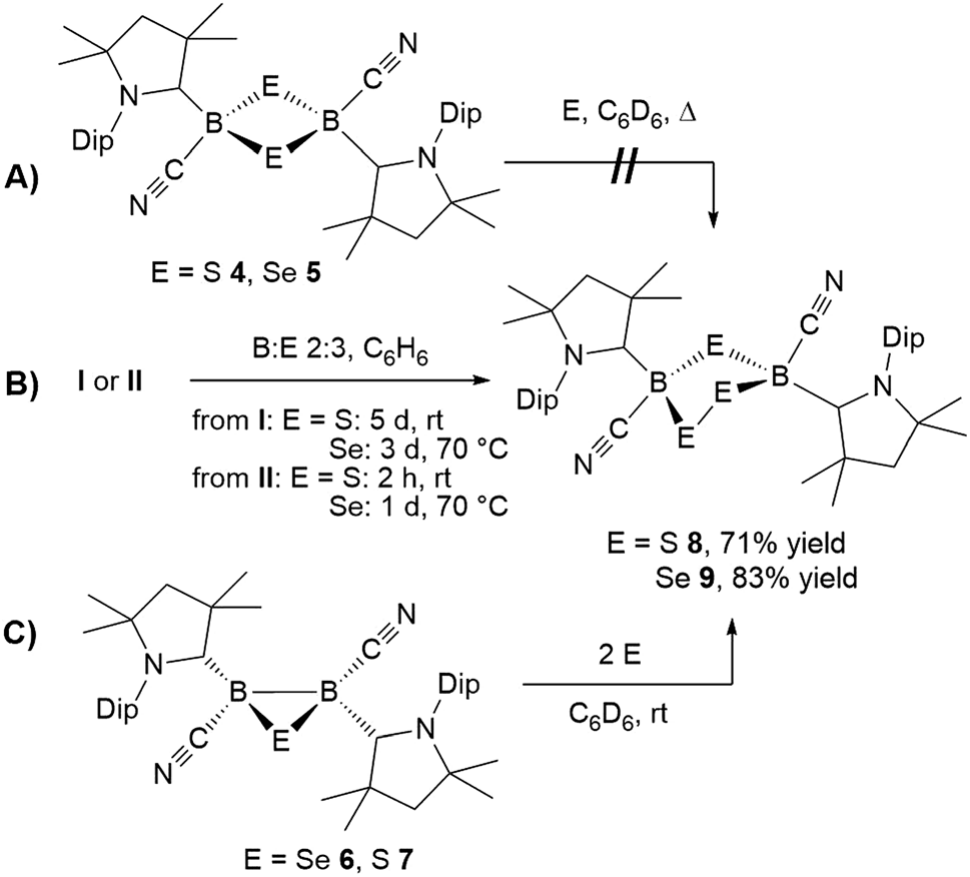
Unsuccessful ring-expansion of 1,3-dichalcogen-2,4-diboretanes (A) and successful syntheses of 1,2,4-trichalcogena-3,5-diborolanes from **I** or **II** (B) and by ring-expansion of 1,2,3-dichalcogenadiboriranes (c).

In repeated X-ray crystallographic experiments on single crystals in **4** the refinement of the data generated some residual electron density around the B_2_S_2_ ring that hinted at its overlap with a five-membered 1,2,4-dithia-3,5-diborolane ring representing less than 5% of the structure. This was consistent with the observation that recrystallized samples of **4** always showed some contamination with another species showing an ^11^B NMR resonance at –9 ppm, and that closer inspection of the mass spectrum of **4** revealed traces of a compound with the formula [(cAAC)_2_B_2_(CN)_2_S_3_]. Based upon this observation, attempts were made to obtain this compound by addition of sulfur to **4**. However, even in the presence of excess sulfur with prolonged heating at 60 °C, only starting material was recovered (Scheme 7A).

In contrast, the room temperature reaction of tetrameric borylene **I** with elemental sulfur in a 2 : 3 boron-to-sulfur ratio yielded the corresponding yellow 1,2,4-trithia-3,5-diborolane **8** as the major reaction product in 71% isolated yield (Scheme 7B). This suggests that **8** is formed directly from **I** and independently of **4**, rather than by insertion of a sulfur atom into the 1,3-disulfa-2,4-diboretane ring. Isolated samples of **8** showed two distinct ^11^B NMR resonances at –8.5 and –9.1 ppm in a 55 : 45 ratio, *ca.* 9 ppm downfield from that of **4**. The ^1^H NMR spectrum of **8** displayed two isomers in that same ratio, each presenting two sets of cAAC resonances in a 1 : 1 ratio. Furthermore, for each isomer, the isopropyl resonances of the Dip residue were split into two sets of asymmetric resonances. The ratio of the two isomers was temperature-independent, suggesting they may be non-exchanging diastereomers.

**Scheme 8:**
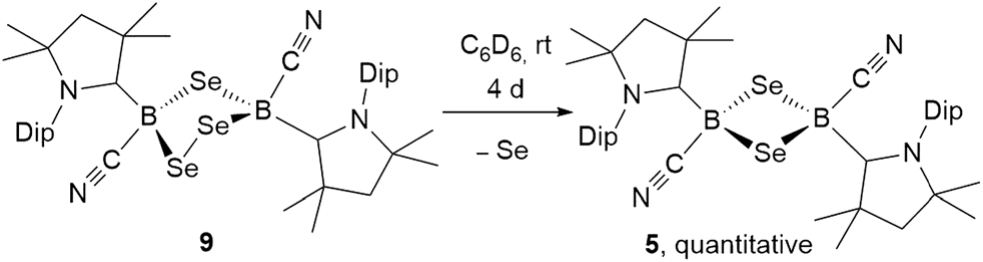
Spontaneous ring contraction of 1,2,4-triselena-3,5-diborolane **9**.

The analogous 1,2,4-triselena-3,5-diborolane **9** was successfully isolated as an orange-colored solid as the major product (83% yield) of the reaction of borylene **I** with selenium in a 2 : 3 boron-to-selenium ratio in benzene at 60 °C (Scheme 7B). Compound **9** presented an ^11^B NMR resonance at –12.3 ppm, *ca.* 20 ppm downfield from that of **5**. Similarly to **8**, the ^1^H NMR resonances of the isoproyl groups of the Dip residue were split into two sets, indicative of an asymmetric structure. Attempts to detect the ^77^Se{^1^H} NMR resonances of **9** failed due to strong broadening of the signal. Furthermore, while the B_2_S_3_ analogue **7** proved stable in solution, compound **9** was observed to fully decompose to the B_2_Se_2_ heterocycle **5** in benzene solution over a period of 4 days at room temperature (Scheme 8).

Both 1,2,4-trichalcogena-3,5-diborolanes **8** and **9** could also be obtained by reacting diborene **II** with three molar equivalents of elemental sulfur or selenium, respectively, or from the reaction of the corresponding B_2_E heterocycles, **7** and **6**, with two molar equivalents of sulfur or selenium, respectively (Scheme 7C).

**Fig. 5:**
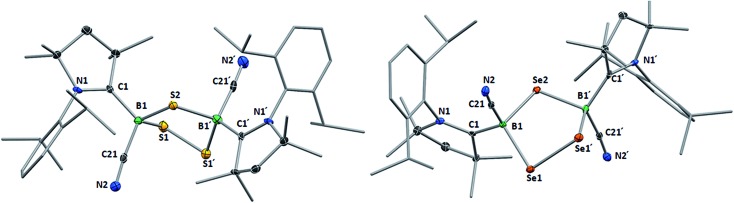
Crystallographically determined solid-state structures of **8** (top) and **9** (bottom) atomic displacement ellipsoids depicted at 50% probability level. Hydrogen atoms and atomic displacement ellipsoids of peripheral substituents omitted for clarity. Selected bond lengths (Å) and angles (°) for **8**: B1–C1 1.622(5), B1–C21 1.587(6), B1–S1 1.943(4), B1–S2 1.931(4), S1–S1′ 2.0680(19); B1–S2–B1′ 101.0(2), S1–B1–S2 107.9(2), B1–S1–S1′ 95.80(13). For **9**: B1–C1 1.614(6), B1–C21 1.579(6), B1–Se1 2.068(4), B1–Se2 2.086(4), Se1–Se1′ 2.3302(9); B1–Se2–B1′ 99.9(2), Se1–B1–Se2 109.4(2), B1–Se1–Se1′ 93.63(13).

Like their B_2_E_2_ counterparts, compounds **8** and **9** crystallized in isomorphous unit cells. For both compounds, the X-ray crystallographic structures (Fig. 5) show a five-membered central 1,2,4-trichalcogena-3,5-diborolane ring bearing one cyano and one cAAC ligand on each boron atom, arranged in a *trans*-configuration with respect to the B_2_E_3_ ring. While the structures thus represent a single (*R*,*R*/*S*,*S*) diastereomer, the observation of two non-exchanging isomers of **8** in solution suggests that the meso diastereomer of **8** may also be formed. The formation of a single diastereomer of **9** may be attributable to the higher reaction temperature favoring the thermodynamic (*R*,*R*/*S*,*S*) diastereomer. The structure of **8** is reminiscent of that of the bis(NHC)-stabilized 3,5-dithienyl-1,2,4-trithia-3,5-diborolane obtained by the reductive insertion of three sulfur atoms into the B=B double bond of a diborene precursor, with very similar B–S bond lengths and B–S–B and S–B–S angles.^13^ The 1,2,4-triselena-3,5-diborolane ring of **9** is reminiscent of that obtained by Tokitoh and co-workers upon irradiation of a boron bis(methylselenide) bound to a very bulky aryl ligand (Tbt = 2,4,6-(C(SiMe_3_)_2_H)_3_C_6_H_2_).^21^ Due to the sp^3^ hybridization of the cAAC-supported boron atoms in **9**, however, the B–Se bonds (2.068(4), 2.086(4) Å) are slightly elongated and the Se–B–Se angle (109.4(2)°) is significantly more acute than in [(Tbt)_2_B_2_Se_3_] (B–Se: 1.942(7), 1.926(8) Å; Se–B–Se: 118.8(4)°).^21^

**Scheme 9:**
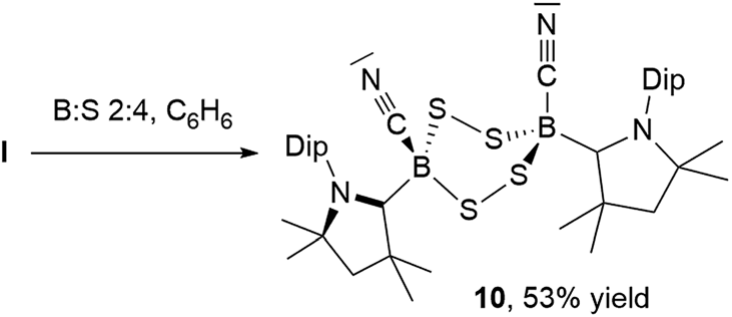
Synthesis of 1,2,4,5-tetrasulfa-3,6-diborinane **10** from **I**.

NMR spectroscopic analysis of the crystallization filtrate of 1,2,4-trithia-3,5-diborolane **8** revealed the presence of another boron-containing species presenting an ^11^B NMR singlet at –11.2 ppm and a single symmetrical cAAC ligand environment in its ^1^H NMR spectrum. Surmising that this may be a tetrathiadiborinane resulting from a 1 : 4 boron-to-sulfur reaction, a scaled-up reaction with this stoichiometry was carried out (Scheme 9). The resulting orange suspension was filtered and the filtrate slowly evaporated to give compound **10** as a pale yellow solid in 53% isolated yield based on boron and its formulation confirmed by LC-MS.

Multiple recrystallizations of **10** from various solvents provided extremely thin, heavily twinned crystals, which diffracted too weakly to provide data suitable for structural discussion. However, the results of these X-ray crystallographic experiments provided conclusive proof of connectivity, confirming that **10** is indeed a bis(cAAC)-stabilized 3,6-dicyano-1,2,4,5-tetrasulfa-3,6-diborinane displaying a *cis*-arrangement of the cyano and cAAC ligands with respect to the central B_2_S_4_ ring, which displays a boat conformation (see Fig. S46† for the solid-state structure of **10**). Attempts to insert a fourth sulfur atom into the isolated 1,2,4-trithia-3,5-diborolane **8** failed to yield **9**, suggesting that, like **8**, compound **9** forms directly from **I**, presumably by dimerization of a monomeric [(cAAC)B(CN)S_2_] intermediate. While 1,2,4,5-tetrasulfa-3,6-diborinanes have been postulated as minor reaction products by both Lappert and later Nöth, based solely on elemental analysis, mass spectrometry and IR spectroscopy data,^22^ compound **10** constitutes the first structurally and NMR-spectroscopically characterized B_2_S_4_ heterocycle.

### Mechanistic considerations

**Scheme 10:**
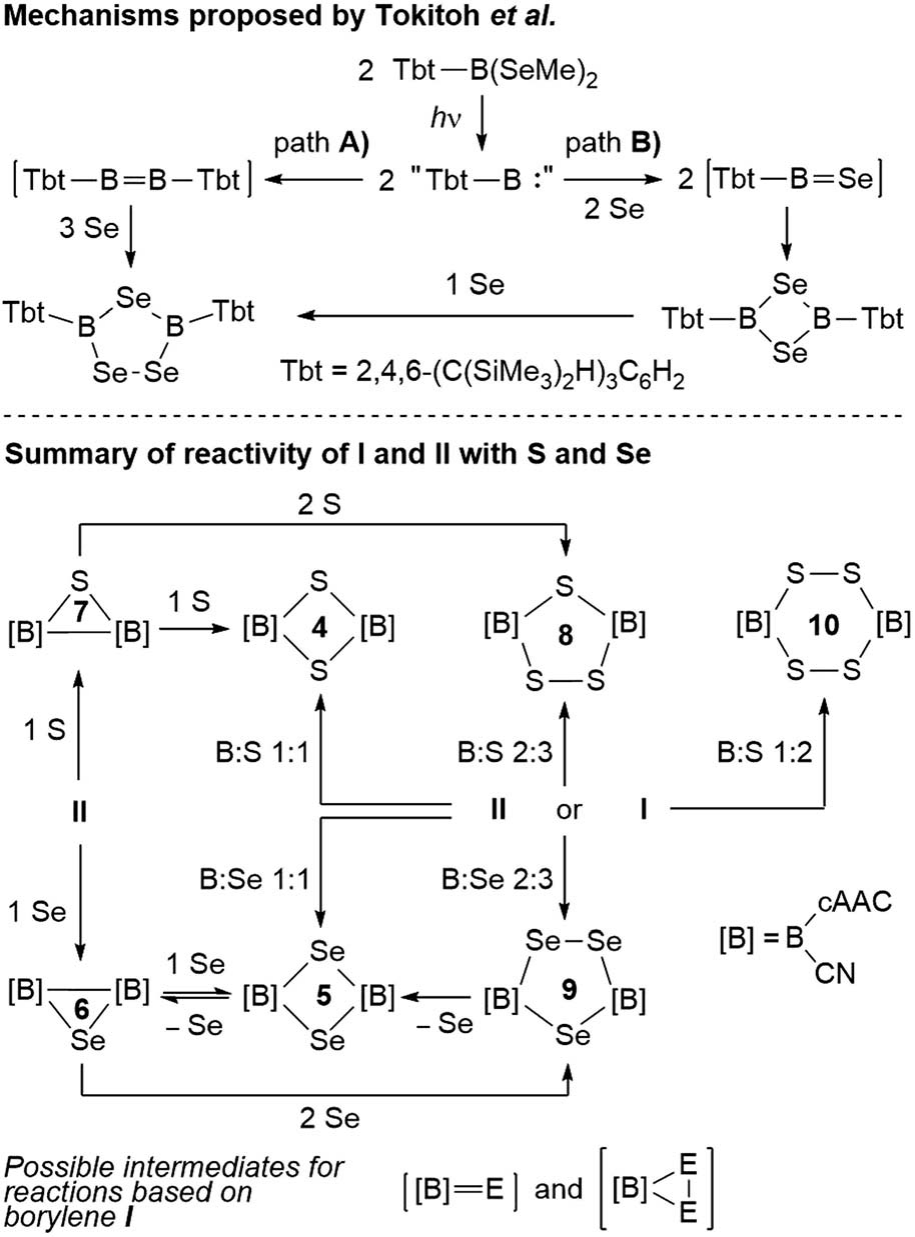
Possible pathways to the boron–chalcogen heterocycles presented herein.

With all these data in hand, it was now possible to reassess the viability of the mechanism proposed by Tokitoh and co-workers for the formation of [(Tbt)_2_B_2_Se_3_] from [(Tbt)B(SeMe)_2_].^21^ In a first step, the authors postulated the formation of a “(Tbt)B:” borylene intermediate upon irradiation of [(Tbt)B(SeMe)_2_]. This monomeric borylene may then dimerize to an electron-deficient diborene [(Tbt)B=B(Tbt)] (Scheme 10, path **A**), which then undergoes the reductive insertion of three atoms of selenium atoms with full cleavage of the B=B bond. For path **B**, the authors proposed a monomeric [(Tbt)B=Se] intermediate resulting from the reaction of “(Tbt)B:” with Se, which dimerizes to a 1,3-diselena-2,4-diboretane and finally inserts the third selenium atom. Regarding path **A**, numerous attempts on our part have failed to convert borylene **I** into its diborene relative, compound **II**, under thermal and/or photolytic conditions. The fact that the three-membered B_2_E heterocycles **6** and **7** can only be accessed directly from diborene **II**, but not from borylene **I**, is further evidence that, despite extensive overlap of reactivity outcomes, **I** and **II** do not interconvert.

Our previous report on the deaggregation of tetramer **I** by Lewis bases showed that only a relatively small and strong Lewis base – the NHC 1,3,4,5-tetramethylimiazol-2-ylidene – is able to break up the tetramer and generate the mixed base-stabilised [(cAAC)(NHC)B(CN)] borylene.^9^ This leads us to conclude that, while acting in all appearance as a source of monomeric borylene “[B]:” ([B] = (cAAC)(CN)B, Scheme 10), such a species is not, in fact, ever present in solution. Reactivity with **I** instead occurs by association of the reactant with the tetramer itself, ultimately causing it to deaggregate into mononuclear intermediates and products. For reactions involving **I**, the generation of intermediate [[B]=E] (E = S, Se) monomers following path **B** therefore seems the most likely. While the dimerization of [(cAAC)(CN)B=E] *via* bridging chalcogen atoms provides compounds **4** and **5**, the latter do not insert another chalcogen atom to form **8** and **9**, respectively, under the reaction conditions employed herein (Scheme 10). This means that the addition of the third chalcogen atom must occur prior to ring-closure, leading us to propose a monomeric dichalcogenaborirane [[B]E_2_] intermediate, which upon reaction with [[B]=E] yields the B_2_E_3_ heterocycles **8** and **9**. Similarly, the B_2_S_4_ heterocycle **10** would result from the dimerization of [[B]S_2_].

It is noteworthy that, while the selenium-based reactions were highly selective, those based on sulfur always yielded a mixture of products. For example, based on ^11^B NMR spectroscopic analysis of the final reaction mixture, the reaction of **I** with sulfur in a 2 : 3 boron-to-sulfur ratio yielded **8** in 70–85% selectivity at most, alongside the smaller and larger heterocycles **4** and **10**, whereas the analogous reaction with selenium provided **9** in near-quantitative yield. The increased selectivity in the formation of **9** may be ascribed to the possibility of selenium de-insertion, which allows any B_2_Se_2_ heterocycle (**5**) formed in the course of the reaction to lose a Se atom, forming the diboraselenirane **6**, which can in turn be converted to **9** (Scheme 10). In contrast, any B_2_S_2_ (**4**) and B_2_S_4_ (**10**) heterocycles formed in the course of the reaction are inert towards sulfur de-insertion and will therefore remain as by-products. Finally, the fact that the four- and five-membered B_2_E_2_ and B_2_E_3_ heterocycles are inert towards chalcogen insertion, whereas the three-membered B_2_E heterocycles may be converted to both the larger B_2_E_2_ and B_2_E_3_ heterocycles, suggests that ring-expansion only proceeds by insertion of a single chalcogen atom or an E_2_ unit into any remaining B–B bonds.

## Conclusions

**Scheme 11:**
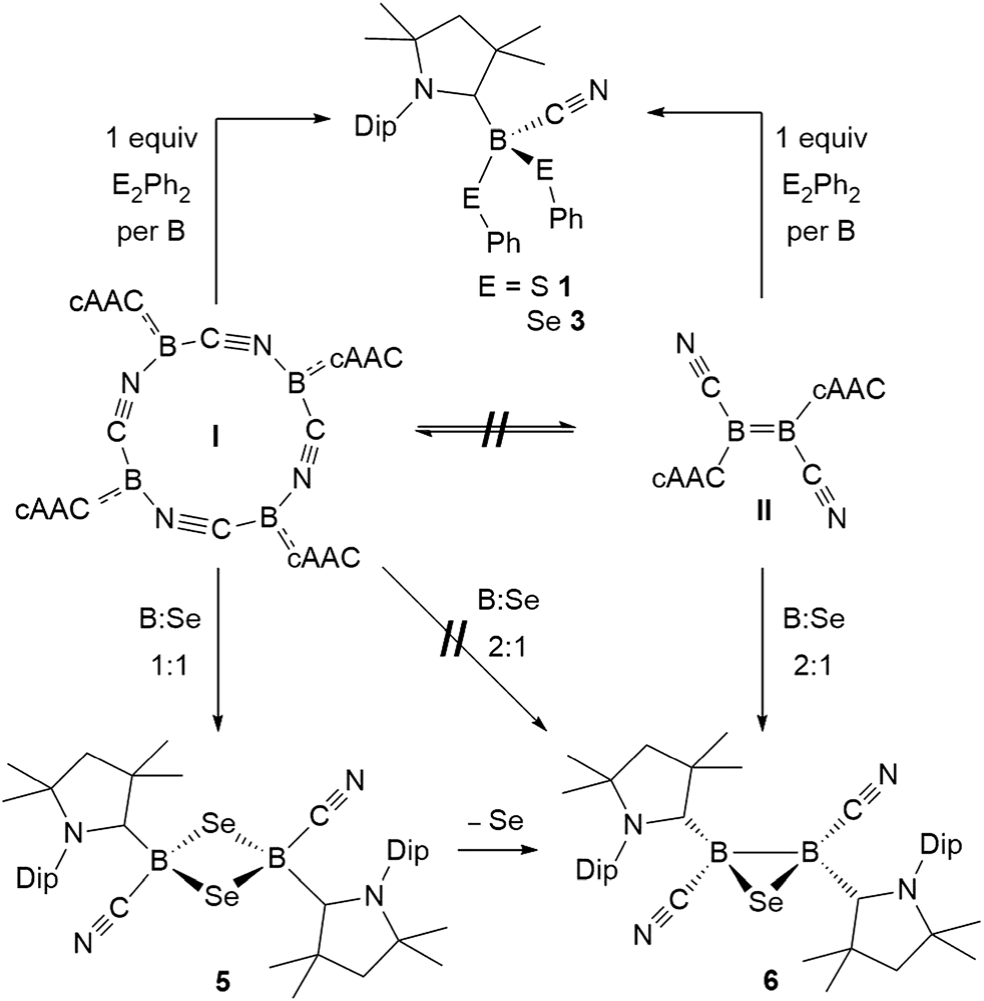
Selected reactions showing the convergent and divergent reactivity of the two boron(i) isomers **I** and **II** with dichalcogenides and elemental selenium.

This first comparative study on the reactivity of two boron(i) compounds of the same empirical formula, the tetrameric, self-stabilizing cyanoborylene **I** and its dicyanodiborene relative **II**, has demonstrated that, while both compounds provide access to the same products in many cases, this occurs *via* different pathways as shown in Scheme 11.

In reactions with dichalcogenides, both **I** and **II** yielded the mononuclear cyanoboron bis(chalcogenides) as sole products (Scheme 11). In the case of **I** this presumably occurs by insertion of a monomeric borylene into the chalcogen–chalcogen bond, whereas for **II** a successive 1,2-addition mechanism across the B=B double bond, resulting in full B–B bond cleavage is most likely at work.

Reactions with elemental sulfur and selenium were found to be highly dependent on the stoichiometry used. Reactions of **I** or **II** employing a 1 : 1 or 2 : 3 boron-to-chalcogen ratio yielded the corresponding 1,3-dichalcogena-2,4-diboretanes or 1,2,4-trichalcogena-3,5-diborolanes, respectively. A unique 1,2,3-thiadiborirane could only be accessed from the reaction of diborene precursor **II** with sulfur in a 2 : 1 boron-to-sulfur ratio, whereas the corresponding diboraselenirane was accessible both directly from the analogous reaction with selenium, and indirectly by de-insertion of one selenium atom from the four-membered 1,3-diselena-2,4-diboretane (Scheme 11). In the case of sulfur a rare example of a 1,2,4,5-tetrasulfa-3,6-diborinane was isolated from the reaction of borylene **I** with sulfur in a 1 : 2 boron-to-sulfur ratio.

Careful stepwise addition of chalcogen equivalents to either **I** or **II** and stability studies of the resulting heterocycles also gave insight into several mechanistic aspects of these reactions:

(i) Borylene-based reactions do not proceed *via* a diborene intermediate but likely *via* monomeric borachalcone and dichalcogenaborirane intermediates;

(ii) Ring-expansion reactions can only proceed by insertion of chalcogens into existing B–B bonds;

(iii) Ring-contraction is possible in the case of boron–selenium heterocycles only by de-insertion of Se atoms.

Although the subtle divergences in reactivity between **I** and **II** provide confirmation that no Wanzlick-type equilibrium exists between a putative monomeric form of **I** and its B–B bonded dimer, diborene **II**, the question remains as to whether this is a result of the extremely stable, tetrameric constitution of borylene **I**. To date, **I** and **II** represent the only existing borylene/diborene pair with the same empirical formula, however, recent advances in the synthesis of borylenes will hopefully enable a more definitive answer to the question of a possible interconversion between [LRB:] borylenes and [LRB=BRL] diborenes. Beyond the interest of such an interconversion from a fundamental point of view, its undeniable potential for providing a new, more reliable route towards hitherto inaccessible diborenes should continue to stimulate research into this area.

## Conflicts of interest

The authors declare no conflicts of interest.

## Supplementary Material

Supplementary information

Crystal structure data
