# Supplementary material for: Closely related yet different: a borylene and its dimer are non-interconvertible but connected through reactivity
Source: Chem Sci. 2018 Jan 4;9(8):2252–60. doi: 10.1039/c7sc04789d (PMC5897878; doi:10.1039/c7sc04789d)
Supplement: Supplementary file 1 [file SC-009-C7SC04789D-s001.pdf]

Electronic Supplementary Material (ESI) for Chemical Science.
This journal is © The Royal Society of Chemistry 2018

Supporting information

Closely Related yet Different: a Borylene and its Dimer are Non-Interconvertible but Connected through Reactivity

Dominic Auerhammer,[a,b] Merle Arrowsmith,[a,b] Julian Böhnke,[a,b] Holger Braunschweig,[a,b,†] Rian D. Dewhurst[a,b] and Thomas Kupfer[a,b]

[a] Institut für Anorganische Chemie, Julius-Maximilians-Universität Würzburg, Am Hubland, 97074 Würzburg (Germany)

[b] Institute for Sustainable Chemistry & Catalysis with Boron, Julius-Maximilians-Universität Würzburg, Am Hubland, 97074 Würzburg (Germany)

Table of contents

Methods and materials 2
NMR spectra of isolated compounds 7
Mass Spectrometry 38
UV-vis spectroscopy 46
X-ray crystallographic details 47
References 52

Methods and materials

All manipulations were performed either under an atmosphere of dry argon or in vacuo using standard Schlenk line or glovebox techniques. Deuterated solvents were dried over molecular sieves and degassed by three freeze-pump-thaw cycles prior to use. All other solvents were distilled and degassed from appropriate drying agents. Solvents (both deuterated and non-deuterated) were stored under argon over activated 4 Å molecular sieves. NMR spectra were acquired on a Bruker Avance 300 NMR spectrometer (^{1}H: 300.1 MHz, ^{77}Se{^{1}H}: 57.25 MHz), a Bruker Avance 400 NMR spectrometer (^{1}H: 400.1 MHz, ^{11}B: 128.3 MHz) and a Bruker Avance 500 NMR spectrometer (^{1}H: 500.1 MHz, ^{11}B: 160.5 MHz, ^{13}C{^{1}H}: 125.8 MHz, ^{77}Se{^{1}H}: 95.38 MHz). Chemical shifts (δ) are given in ppm and internally referenced to the carbon nuclei (^{13}C{^{1}H}) or residual protons (^{1}H) of the solvent. ^{11}B and ^{77}Se{^{1}H} NMR spectra are referenced to external standards [BF_3·OEt_2] or Me_2Se, respectively. UV/vis spectra were acquired on a JASCO-V660 UV/vis spectrometer. High-resolution mass spectrometry was obtained from a Thermo Scientific Exactive Plus spectrometer. Compounds **I**[1] and **II**[2] were synthesized according to literature procedures.

Synthesis and characterization

[(cAAC)B(CN)(SPh)$_2$], 1

Ph_2S_2 (33.8 mg, 155 µmol) and **I** (50.0 mg, 38.8 µmol) were dissolved in 1 mL of benzene. The resulting orange reaction mixture was heated at 60 °C for 80 h. After cooling the mixture was filtered and the remaining solid extracted with C_6D_6. The filtrate was dried in vacuo and the crude solid recrystallized from THF to give **1** as a colourless solid (56.1 mg, 104 µmol, 67%). ^{1}H NMR (500 MHz, C_6D_6): δ = 7.72-7.57 (br m, 4H, Ar*H*), 6.95-7.07 (m, 9H, Ar*H*), 2.85 (sept, ^3J = 6.6 Hz, 2H, CH_{iPr}), 1.57-1.80 (broad, overlapping resonances, 12H, $CH_{3\text{-}iPr}$ and CH_3), 1.47 (s, 2H, CH_2), 1.08 (d, ^3J = 6.6 Hz, 6H, $CH_{3\text{-}iPr}$), 0.90 (br s, 6H, CH_3) ppm. ^{13}C{^{1}H} NMR (125.8 MHz, C_6D_6): δ = 146.4, 141.5, 137.2 (C^q), 136.0, 131.0, 129.3, 127.5, 126.6, 125.9 (CH_{Ar}), 67.8, 55.2 (C^q), 51.7 (CH_2), 31.3 (*C*H), 29.5, 28.3, 26.8, 25.8, 25.2 (CH_3) ppm. Note: the $C_{carbene}$ resonance could not be detected by HMBC due to broadening of all resonances. ^{11}B NMR (160.5 MHz, C_6D_6): δ = –9.6 (s) ppm. LIFDI (*m/z*) calculated for [$C_{33}H_{41}B_1N_2S_2$–CN] = 514.2768; found: 514.2762; and calculated for [$C_{33}H_{41}B_1N_2S_2$–SPh] = 431.2687; found: 431.2682.

[(cAAC)B(CN)(SeMe)$_2$], 2

A solution of Me_2Se_2 (67.0 mg, 356 µmol) in 1 mL of benzene was added to a solution of **I** (122 mg, 94.6 µmol) in 1 mL of benzene. The yellow reaction mixture was stirred for 1 h at rt. The resulting orange solution was dried in vacuo and the crude solid recrystallized from diethyl ether to give **2** as a pale orange solid (160 mg, 314 µmol, 88%). ^{1}H NMR (500 MHz, CD_2Cl_2): δ = 7.48 (t, ^3J = 7.85 Hz, 1H, *p*-Ar*H*), 7.28 (d, ^3J = 7.8 Hz, 2H, *m*-Ar*H*), 2.79 (sept, ^3J = 6.6 Hz, 2H, CH_{iPr}), 2.13 (s, 2H, CH_2), 1.82 (s, 6H, CH_3), 1.74 (s, 6H, CH_3), 1.40-1.36 (overlapping d and s, 6H, $CH_{3\text{-}iPr}$ and CH_3), 1.32 (d, ^3J = 6.6 Hz, 6H, $CH_{3\text{-}iPr}$) ppm. ^{13}C{^{1}H} NMR (125.8 MHz, CD_2Cl_2): δ = 215.1 ($C_{carbene}$ detected by HMBC), 146.7 (C^q), 130.9, 125.8 (CH_{Ar}), 79.7 (C^q), 52.0 (CH_2), 30.7 (CH_3), 29.5 (*C*H), 29.2, 27.4, 25.0, 4.79 (CH_3) ppm. ^{11}B NMR (160.5 MHz, CD_2Cl_2): δ = –18.4 (s) ppm. ^{77}Se{^{1}H} NMR (95.38 MHz, CD_2Cl_2): δ = –102.1 ppm. LIFDI (*m/z*) calculated for [$C_{23}H_{37}B_1N_2Se_2$] = 512.1375; found: 512.1371.

[(cAAC)B(CN)(SePh)$_2$], 3

A solution of Ph_2Se_2 (194 mg, 621 µmol) in 1 mL of benzene was added to a solution of **I** (200 mg, 155 µmol) in 1 mL of benzene. The yellow reaction mixture was stirred for 4 d at rt. The resulting orange solution was dried in vacuo and the crude solid recrystallized from THF to give **3** as an orange solid (291 mg, 459 µmol, 74%). ^{1}H NMR (500 MHz, C_6D_6): δ = 8.00-7.57 (m, 4H, Ar*H*), 7.09-6.96 (m, 9H, Ar*H*), 2.83 (sept, ^3J = 6.6 Hz, 2H, CH_{iPr}), 1.56-1.88 (broad, overlapping resonances, 12H, $CH_{3\text{-}iPr}$ and CH_3), 1.49 (s, 2H, CH_2), 1.08 (d, ^3J = 6.6 Hz, 6H, $CH_{3\text{-}iPr}$), 0.77-1.03 (broad, 6H, CH_3) ppm. ^{13}C{^{1}H} NMR (125.8 MHz, C_6D_6): δ = 146.7, 137.6 (C^q), 136.5, 132.5, 131.2, 127.2, 126.1 (CH_{Ar}), 77.7, 55.2 (C^q), 52.0 (CH_2), 31.9 (CH_3), 29.5 (*C*H), 27.5, 26.6, 25.6 (CH_3) ppm. Note: the $C_{carbene}$ resonance could not be detected by HMBC due to broadening of all resonances. ^{11}B NMR (160.5 MHz, C_6D_6): δ = –14.4 (major), –15.8 (minor) ppm. ^{77}Se{^{1}H} NMR

(95.4 MHz, C_6D_6): δ = 160.2 ppm. LIFDI (*m/z*) calculated for [$C_{33}H_{40}B_1N_2Se_2$] = 635.1609; found: 635.1596.

[(cAAC)B(CN)S]$_2$, 4

A suspension of S_8 (10.0 mg, 39.0 µmol, 0.5 equiv) in 2 mL of benzene was added to a solution of **I** (100 mg, 77.6 µmol) in 1 mL of benzene. The yellow reaction mixture was stirred for 5 d at rt. The resulting orange suspension was filtered and the filtrate slowly evaporated to give **4** as a yellow solid (98.0 mg, 137 µmol, 88% based on boron). Note: analytically pure **4** could not be obtained due to co-crystallization of small amounts of the reaction byproduct **8** (ca. –9.0 ppm), the formation of which could not be avoided. ^{1}H and ^{11}B NMR data of **4** was obtained from an 82:18 mixture of **4** and **8**. The overlap of the majority of ^{13}C NMR resonances of **4** and **8** prevented analysis of the ^{13}C{^{1}H} spectrum. ^{1}H NMR (400 MHz, CD_2Cl_2): δ = 7.42-7.37 (m, 2H, *p*-Ar*H*), 7.23-7.20 (m, 4H, *m*-Ar*H*), 2.68 (sept, ^{3}J = 6.7 Hz, 4H, CH_{iPr}), 2.09 (s, 4H, CH_2), 1.98 (s, 12H, CH_3), 1.34 (s, 12H, CH_3), 1.30 (d, ^{3}J = 6.6 Hz, 12H, C$H_{3\text{-iPr}}$), 1.25 (d, ^{3}J = 6.6 Hz, 12H, C$H_{3\text{-iPr}}$) ppm. ^{11}B NMR (128.3 MHz, CD_2Cl_2): δ = –17.9 (s) ppm. ASAP pos (*m/z*) calculated for [$C_{42}H_{62}B_2N_4S_2$+H]$^+$ = 709.4675; found: 709.4657.

[(cAAC)B(CN)Se]$_2$, 5

A suspension of elemental Se (65.0 mg, 823 µmol, 4 equiv) in 1 mL of benzene was added to a solution of **I** (250 mg, 194 µmol) in 1 mL of benzene. The yellow reaction mixture was stirred for 3 d at 70 °C. The resulting orange suspension was filtered and the filtrate slowly evaporated to give **5** as an orange solid (218 mg, 543 µmol, 70%). Note: compound **5** was isolated as a single isomer (δ_{11B} –33.5 ppm) which, over a period of 3 d at rt, partially isomerized (ca. 17%) to a second isomer (δ_{11B} –31.8 ppm). ^{1}H NMR (500 MHz, CD_2Cl_2), major isomer: δ = 7.42 (t, ^{3}J = 7.7 Hz, 1H, *p*-Ar*H*), 7.23 (d, ^{3}J = 7.7 Hz, 2H, *m*-Ar*H*), 2.61 (sept, ^{3}J = 6.6 Hz, 2H, CH_{iPr}), 2.12 (s, 6H, CH_3), 1.69 (s, 2H, CH_2), 1.33 (s, 6H, CH_3), 1.30 (d, ^{3}J = 6.6 Hz, 6H, C$H_{3\text{-iPr}}$), 1.26 (d, ^{3}J = 6.6 Hz, 6H, C$H_{3\text{-iPr}}$) ppm; minor isomer: δ = 7.43 (t, ^{3}J = 7.7 Hz, 1H, *p*-Ar*H*), 7.25 (d, ^{3}J = 7.7 Hz, 2H, *m*-Ar*H*), 2.68 (sept, ^{3}J = 6.6 Hz, 2H, CH_{iPr}), 2.04 (s, 6H, CH_3), 1.76 (s, 2H, CH_2), 1.58 (d, ^{3}J = 6.6 Hz, 6H, C$H_{3\text{-iPr}}$), 1.32 (s, 6H, CH_3), 1.28 (d, ^{3}J = 6.6 Hz, 6H, C$H_{3\text{-iPr}}$) ppm. ^{13}C{^{1}H} NMR (125.8 MHz, CD_2Cl_2) major isomer only: δ = 217.9 (br, $C_{carbene}$ detected by HMBC), 146.1, 134.0 (C^q), 130.5, 125.6 (CH_{Ar}), 77.9, 56.2 (*C*q), 53.0 (identified by HSQC, CH_2), 32.2 (CH_3), 29.4 (CH), 28.5, 26.7, 24.9 (CH_3) ppm. ^{11}B NMR (160.5 MHz, CD_2Cl_2): δ = –31.8 (s) (minor), –33.5 (s) (major) ppm. ^{77}Se{^{1}H} NMR (95.4 MHz, CD_2Cl_2): δ = –143.1 (s) ppm. LIFDI (*m/z*) calculated for [$C_{42}H_{62}B_2N_4Se_2$+H]$^+$ = 804.3486; found: 804.3473.

[(cAAC)B(CN)]$_2$Se, 6

From **I**: A few mg of crystals of **6** were obtained by slow crystallization from a NMR solution of **5** in CD_2Cl_2 left undisturbed for several days at rt. The amount was sufficient for X-ray crystallographic analysis and MS but not for acquiring NMR data. Attempts to generate **6** selectively by reacting tetramer **I** with 4 equiv Se only resulted in partial conversion to **5** and unreacted **I**.

From **II**: The reaction of diborene **II** (20 mg, 31 µmol) with 1 equiv elemental selenium (2.4 mg, 31 µmol) in 0.5 mL C_6H_6 at room temperature afforded compound **6** selectively as the sole reaction product, which was isolated as a yellow crystalline solid after solvent removal and washing with pentane (18 mg, 26 µmol, 84%).
^{1}H NMR (500 MHz, C_6D_6): 6.90-6.94 (m, 6H, Ar-*H*), 3.38, 2.60 (two sept, ^3J = 6.7 Hz, 2H each, CH_{iPr}), 2.19, 2.00 (two s, 6H each, CH_3), 1.74, 1.67 (two d, ^3J = 6.7 Hz, 6H each, C$H_{3\text{-}iPr}$), 1.61, 1.47 (two AB doublets, ^2J = 12.6 Hz, 2H each, CH_2), 1.58 (s, 6H, CH_3), 1.16, 1.09 (two d, ^3J = 6.7 Hz, 6H each, C$H_{3\text{-}iPr}$), 0.56 (s, 6H, CH_3). ^{13}C{^{1}H} NMR (125.8 MHz, C_6D_6): 224.8 (br, $C_{carbene}$ detected by HMBC), 146.5 (C^q), 143.7, 137.6 (C^q), 129.3, 125.5, 124.9 (CH_{Ar}), 76.7, 55.9 (*C*q), 54.4 (CH_2), 33.9, 31.7, 30.1 (CH_3), 29.9, 29.3 (CH), 27.7 (CH_3), 27.1, 26.6, 26.2, 25.3 (CH_3). ^{11}B NMR (160.5 MHz, C_6D_6): –22.0 (s). Note: the ^{77}Se NMR resonance could not be detected due to the strong coupling to the quadrupolar boron nuclei. LIFDI (*m/z*) calculated for [$C_{42}H_{62}B_2N_4Se$] = 724.4320; found: 724.4309.

[(cAAC)$_2$B$_2$(CN)$_2$S], 7

The reaction of diborene **II** (20 mg, 31 µmol) with 1 equiv elemental sulfur (1.0 mg, 31 µmol) in 0.5 mL C_6H_6 at room temperature afforded compound **7** as the sole reaction product, which was isolated as a bright orange crystalline solid after solvent removal and washing with pentane (16 mg, 23 µmol, 74%). ^{1}H NMR (500 MHz, C_6D_6): 6.83-6.93 (m, 6H, Ar-*H*), 3.45, 2.60 (two sept, ^3J = 6.7 Hz, 2H each, CH_{iPr}), 2.21, 1.99 (two s, 6H each, CH_3), 1.69, 1.62 (two d, ^3J = 6.7 Hz, 6H each, C$H_{3\text{-}iPr}$), 1.60, 1.49 (two AB doublets, the first overlapping with a CH_3 singlet, ^2J = 12.6 Hz, 2H each, CH_2), 1.59 (s, 6H, CH_3), 1.17, 1.08 (two d, ^3J = 6.7 Hz, 6H each, C$H_{3\text{-}iPr}$), 0.54 (s, 6H, CH_3). ^{13}C{^{1}H} NMR (125.8 MHz, C_6D_6): 225.8 (br, $C_{carbene}$ detected by HMBC), 146.0 (C^q), 143.6, 136.9 (C^q), 129.1, 125.3, 124.4 (CH_{Ar}), 75.8, 55.4 (*C*q), 53.5 (CH_2), 33.5, 31.6, 29.9 (CH_3), 29.5, 28.8 (CH), 27.1 (CH_3), 26.5, 26.4, 26.3, 24.8 (CH_3). ^{11}B NMR (160.5 MHz, C_6D_6): –22.6 (s). LIFDI (*m/z*) calculated for [$C_{42}H_{62}B_2N_4S$] = 676.4876; found: 676.4860.

[(cAAC)$_2$B$_2$(CN)$_2$S$_3$], 8

A suspension of S_8 (15.0 mg, 58.9 µmol, 0.75 equiv) in 2 mL of benzene was added to a solution of **I** (100 mg, 77.6 µmol) in 1 mL of benzene. The yellow reaction mixture was stirred for 5 d at rt. The resulting orange suspension was filtered and the filtrate slowly evaporated to give **9** as a yellow solid (82.1 mg, 111 µmol, 71% based on boron). NMR spectroscopic data revealed the presence of two non-exchanging isomers in a ca. 55:45 ratio. Major isomer: ^{1}H NMR (500 MHz, CD_2Cl_2): δ = 7.47 (t, ^3J = 7.8 Hz, 1H, *p*-Ar*H*), 7.29, 7.25 (two d, ^3J = 7.8 Hz, ^4J = 1.4 Hz, 1H each, *m*-Ar*H*), 2.81, 2.64 (two sept, ^3J = 6.6 Hz, 1H each, CH_{iPr}), 2.10 (s, 2H, CH_2), 1.90, 1.70 (two s, 3H each, CH_3), 1.46 (overlapping s and d, 3H each, C$H_{3\text{-}iPr}$ and CH_3), 1.31-1.33 (overlapping s and two d, 3H each, C$H_{3\text{-}iPr}$ and CH_3) ppm. ^{13}C{^{1}H} NMR (125.8 MHz, CD_2Cl_2): δ = 219.4 (br, carbene carbon detected by HMBC), 153.2 (*i-C*), 146.5, 146.1, 132.8, 130.9, 125.8, 125.6 (CH_{Ar}), 80.0, 55.8 (*C*q), 52.6 (CH_2), 31.5, 30.9, (CH_3), 29.7, 29.4 (CH), 29.6, 27.6, 27.0, 26.6, 25.5, 24.4 ppm. Minor isomer: 7.40 (t, ^3J = 7.8 Hz, 1H, *p*-Ar*H*), 7.21, 7.18 (two d, ^3J = 7.8 Hz, ^4J = 1.4 Hz, 1H each, *m*-Ar*H*), 2.75, 2.63 (two

sept, ^3J = 6.6 Hz, 1H each, CH_{iPr}), 2.17 (s, 2H, CH_2), 1.85, 1.79 (two s, 3H each, CH_3), 1.43, 1.39 (two s, 3H each, CH_3), 1.37, 1.34 (two d, ^3J = 6.6 Hz, 3H each, C$H_{3\text{-iPr}}$), 1.32, 1.29 (two d, ^3J = 6.6 Hz, 3H each, C$H_{3\text{-iPr}}$) ppm. ^{13}C{^{1}H} NMR (125.8 MHz, CD_2Cl_2): δ = 217.9 (br, $C_{carbene}$ detected by HMBC), 149.7 (*i-C*), 146.3, 146.2, 133.0 (C^q), 131.3, 125.8, 125.7 (CH_{Ar}), 80.3, 55.6 (C^q), 52.2 (CH_2), 31.2, 31.0, 30.0 (CH_3), 29.6, 29.5 (*C*H), 29.2, 27.4, 27.0, 25.0, 24.8 (CH_3) ppm. ^{11}B NMR (160.5 MHz, CD_2Cl_2): δ = –8.5 (s, major), –9.1 (s, minor) ppm. LIFDI (*m/z*) calculated for [$C_{42}H_{62}B_2N_4S_3$] = 740.4317; found: 740.4312.

[(cAAC)$_2$B$_2$(CN)$_2$Se$_3$], 9

A suspension of elemental Se (65.0 mg, 823 µmol, 6 equiv) in 3 mL of benzene was added to a solution of **I** (150 mg, 116 µmol) in 1 mL of benzene. The yellow reaction mixture was stirred for 3 days at 70 °C. The resulting orange suspension was filtered and the filtrate slowly evaporated to give **9** as an orange solid (171 mg, 193 µmol, 83%). ^{1}H NMR (400 MHz, C_6D_6): δ = 7.27-7.20 (m, 2H, *p*-Ar*H*), 7.12-7.03 (m, 4H, *m*-Ar*H*), 2.81, 2.62 (two sept, ^3J = 6.7 Hz, 2H each, CH_{iPr}), 2.27, 1.89 (two s, 6H each, CH_3), 1.79, 1.66 (two d, ^3J = 6.7 Hz, 6H each, C$H_{3\text{-iPr}}$), 1.55-1.35 (m, 4H, CH_2), 1.10, 1.04 (two d, ^3J = 6.7 Hz, 6H each, C$H_{3\text{-iPr}}$), 0.83, 0.73 (two s, 6H each, CH_3) ppm. ^{13}C{^{1}H} NMR (125.8 MHz, C_6D_6): δ = 219.5 (br, $C_{carbene}$ detected by HMBC), 146.5, 146.0 (C^q), 131.0, 125.7, 125.6 (CH_{Ar}), 78.0, 76.9, 55.9 (C^q), 52.1 (CH_2), 34.1, 31.7 (CH_3), 29.5, 29.3 (CH), 28.1, 27.3, 24.8, 24.8, 25.4, 22.7 ppm. ^{11}B NMR (128.3 MHz, C_6D_6): δ = –12.3 (s) ppm. Note: the ^{77}Se NMR resonance could not be detected due to the strong coupling to the quadrupolar boron nuclei. LIFDI (*m/z*) calculated for [$C_{42}H_{62}B_2N_4Se_3$] = 882.2659; found: 882.2661.

[(cAAC)B(CN)S$_2$]$_2$, 10

A suspension of S_8 (20.0 mg, 78.6 µmol, 1 equiv) in 4 mL of benzene was added to a solution of **I** (100 mg, 77.6 µmol) in 1 mL of benzene. The yellow reaction mixture was stirred for 5 d at rt. The resulting orange suspension was filtered and the filtrate slowly evaporated to give **10** as a yellow solid (63.1 mg, 81.7 µmol, 53% based on boron). ^{1}H NMR (500 MHz, CD_2Cl_2): δ = 7.51-7.46 (m, 2H, *p*-Ar*H*), 7.29-7.25 (m, 4H, *m*-Ar*H*), 2.65 (sept, ^3J = 6.7 Hz, 4H, CH_{iPr}), 2.15 (s, 4H, CH_2), 2.03-1.65 (broad, 12H, CH_3), 1.37 (s, 12H, CH_3), 1.33 (d, ^3J = 6.6 Hz, 12H, C$H_{3\text{-iPr}}$), 1.29 (d, ^3J = 6.6 Hz, 12H, C$H_{3\text{-iPr}}$) ppm. ^{11}B NMR (160.5 MHz, CD_2Cl_2): δ = –11.2 (s) ppm. ^{13}C{^{1}H} NMR (125.8 MHz, CD_2Cl_2): δ = 213.9 (br, $C_{carbene}$), 146.1, 132.8 (C^q), 131.0, 125.7 (CH_{Ar}), 80.0, 55.4 (C^q), 52.2 (CH_2), 30.1 (*C*H), 29.5, 29.2, 26.8, 24.8 (CH_3) ppm. LIFDI (*m/z*) calculated for [$C_{42}H_{62}B_2N_4S_4$] = 772.4038; found: 772.4025.

NMR spectra of isolated compounds

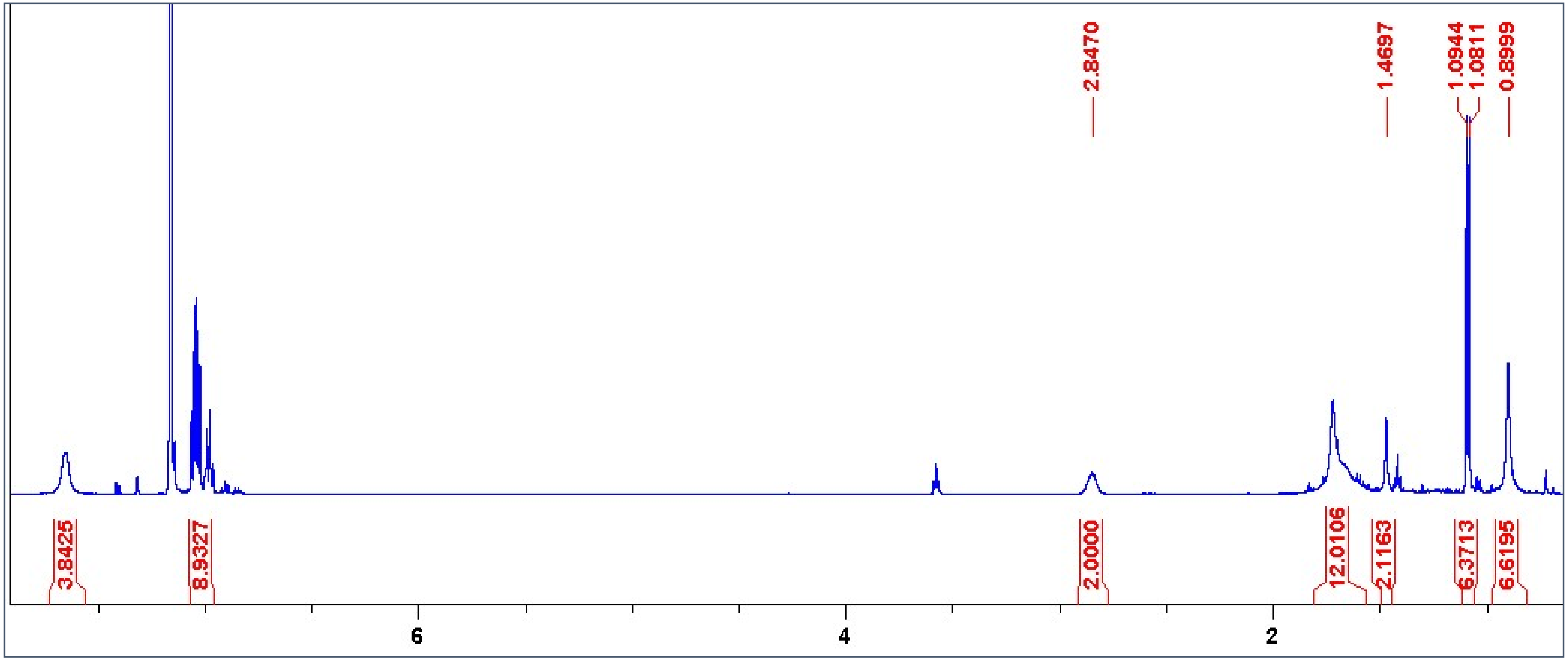

Figure S1. ^{1}H NMR spectrum of **1** in C_6D_6.

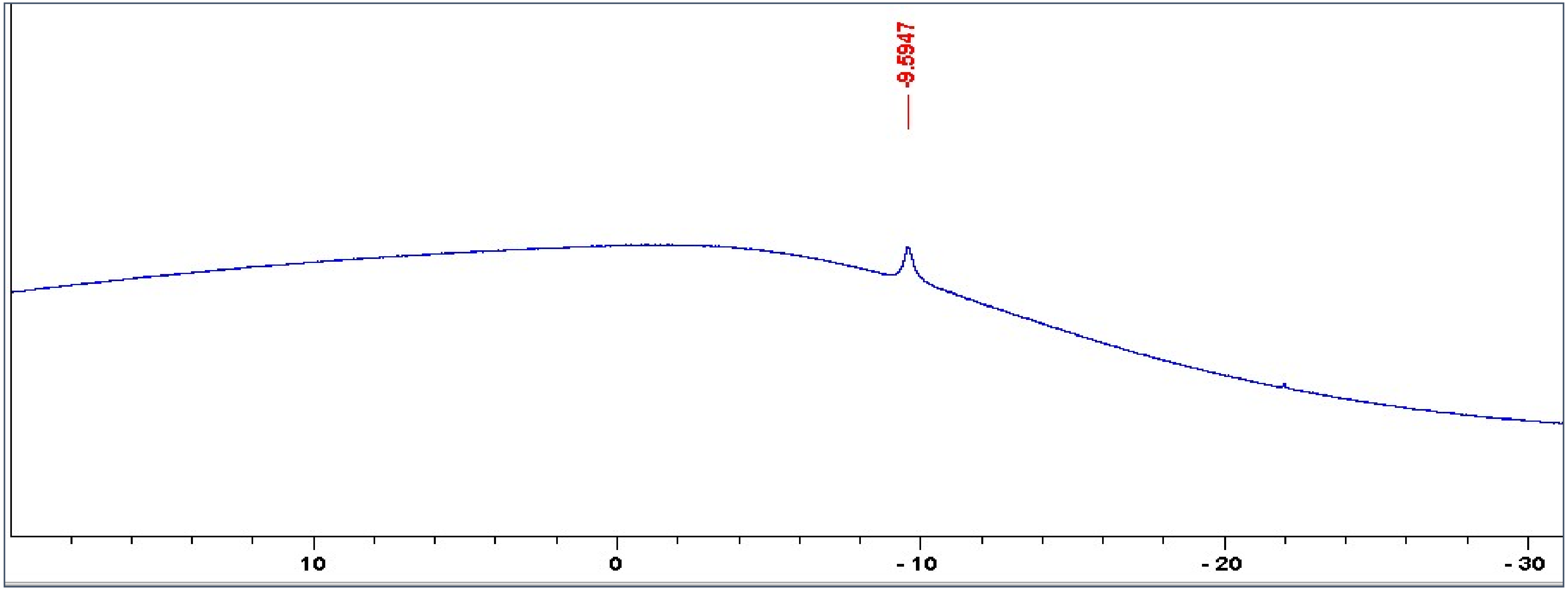

Figure S2. ^{11}B NMR spectrum of **1** in C_6D_6.

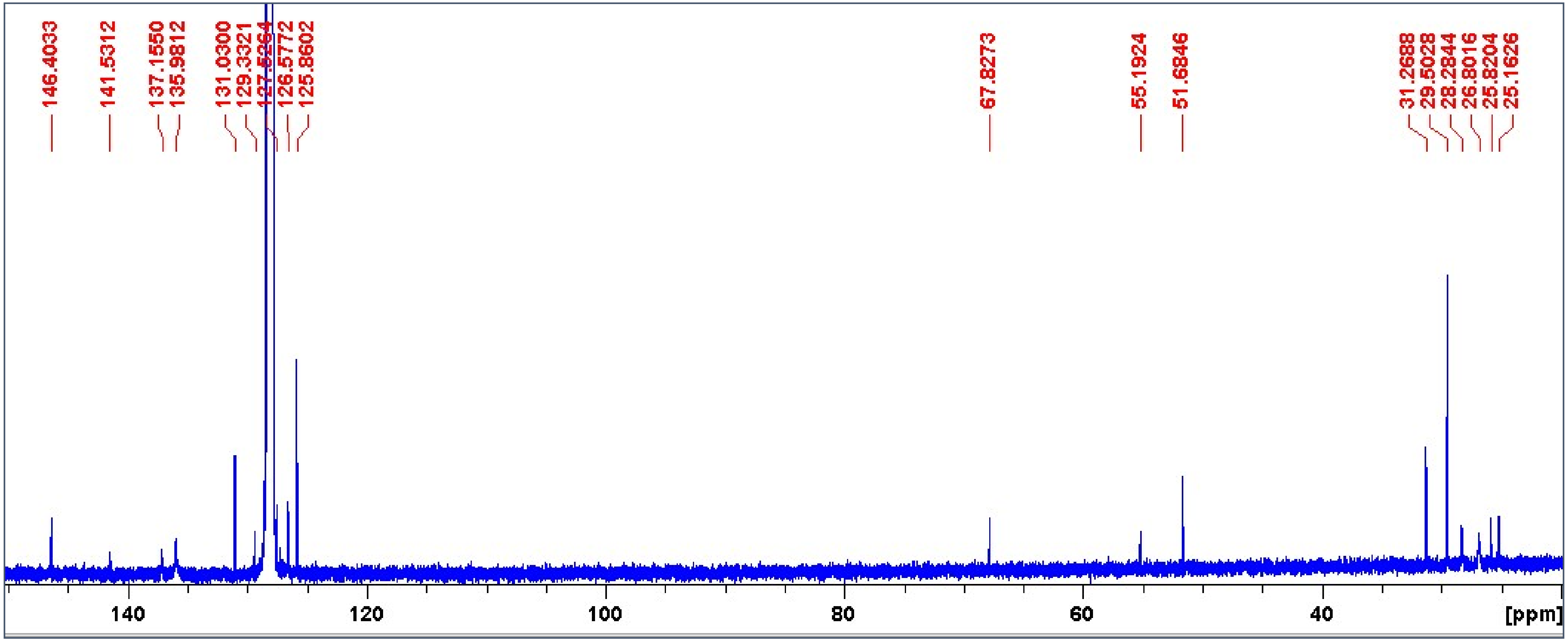

Figure S3. $^{13}C\{^1H\}$ NMR spectrum of **1** in C_6D_6.

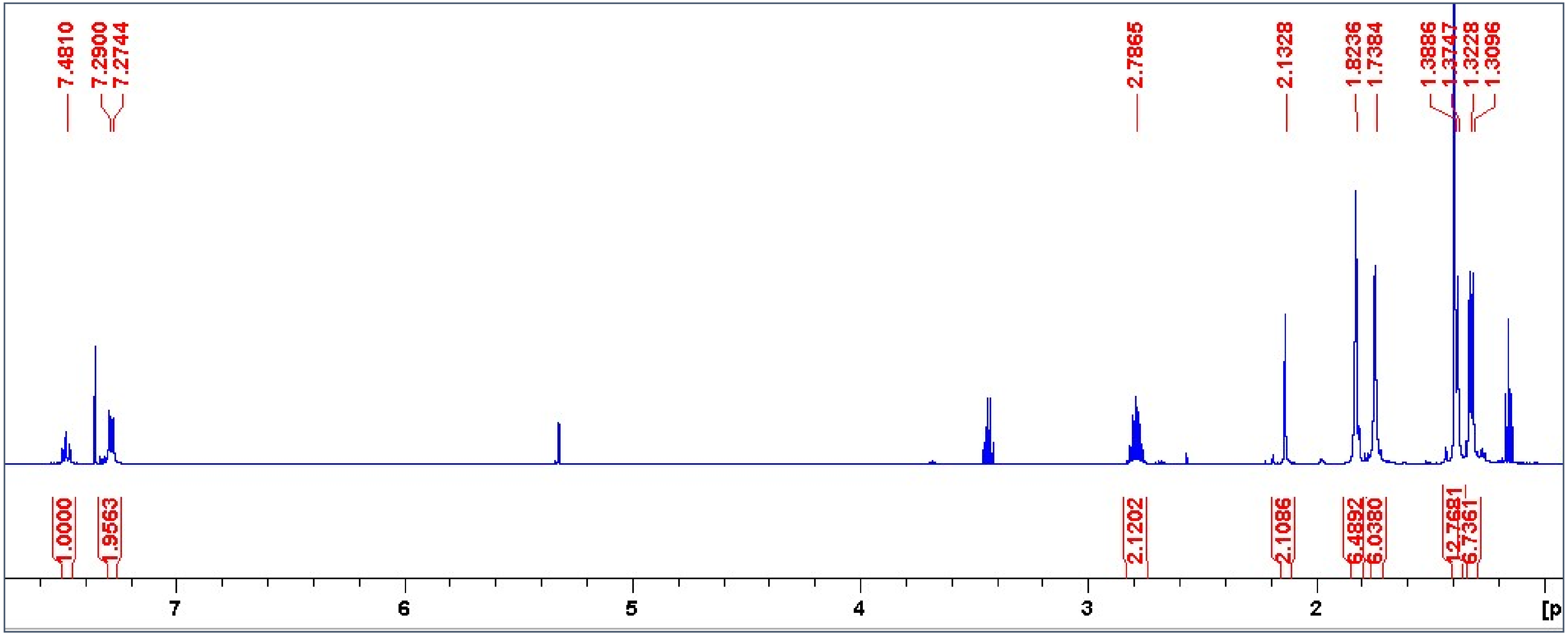

Figure S4. ^{1}H NMR spectrum of **2** in CD_2Cl_2. Additional resonance at 7.35 corrresponds to residual benzene, those at 3.4 (q) and 1.2 (t) ppm to residual Et_2O (crystallisation solvents).

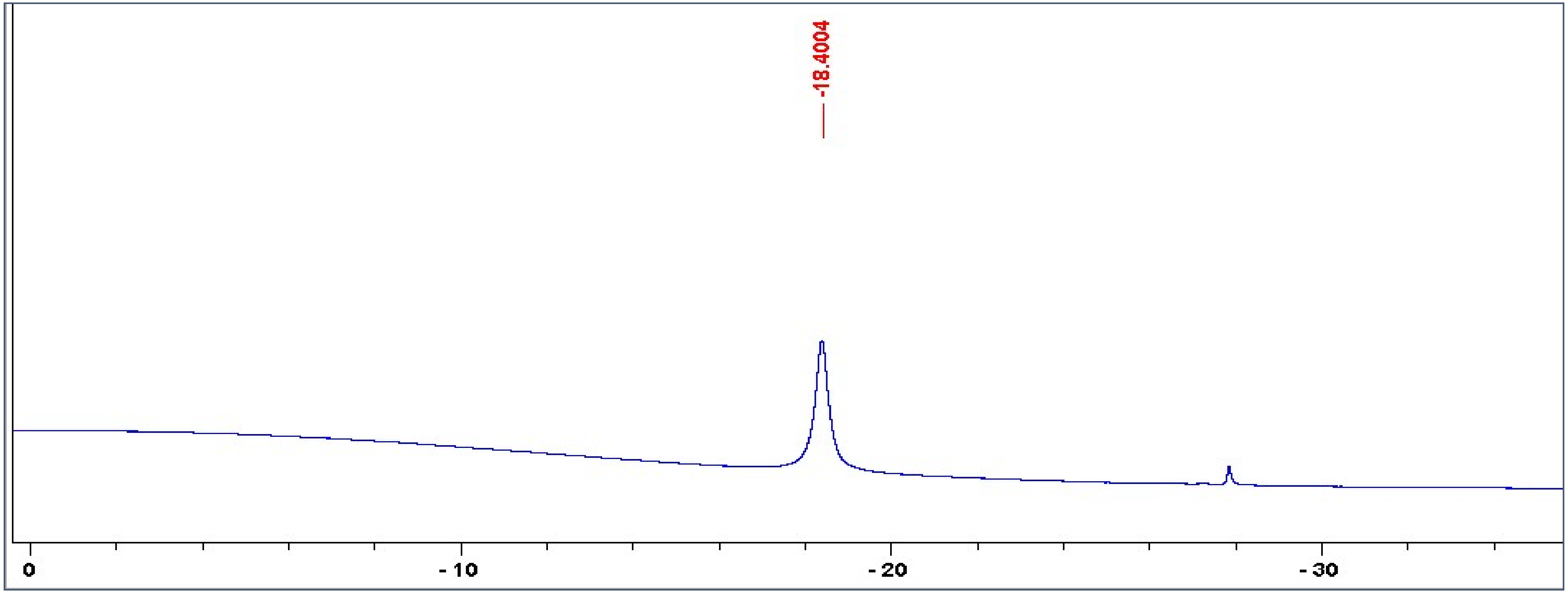

Figure S5. ^{11}B NMR spectrum of **2** in CD_2Cl_2. The small impurity at –28 ppm is the result of partial hydrolysis during isolation due to the extreme moisture-sensitivity of compound **2**.

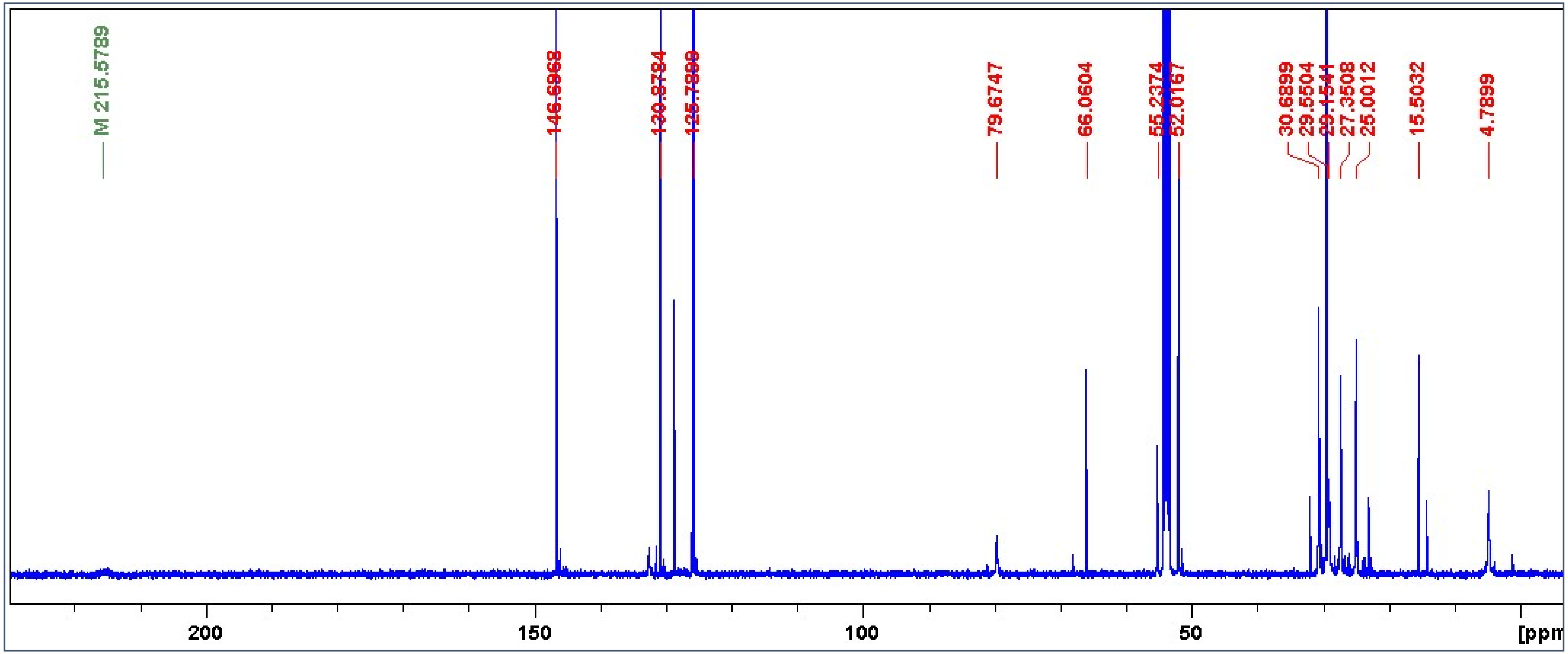

Figure S6. $^{13}C\{^{1}H\}$ NMR spectrum of **2** in CD_2Cl_2 (in green: manually picked broad resonance).

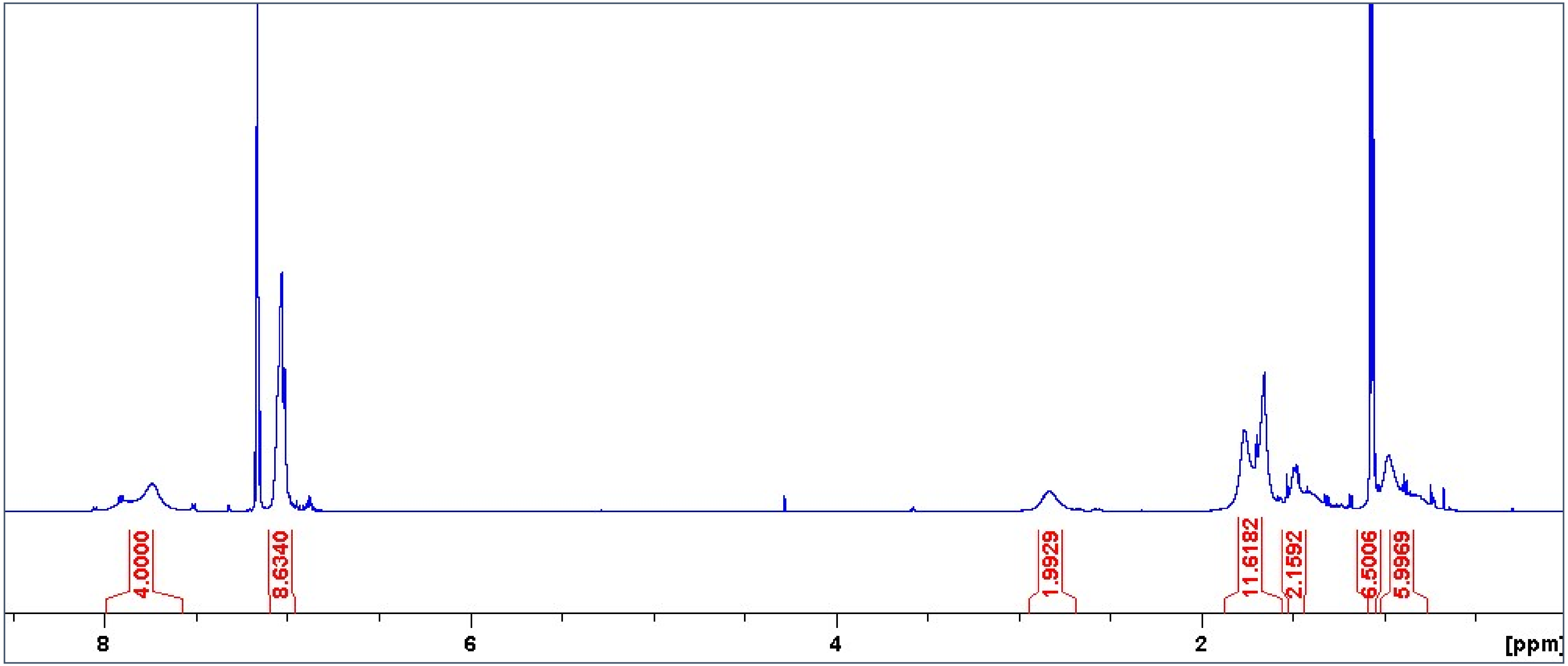

Figure S7. ^{1}H NMR spectrum of **3** in C_6D_6 at rt.

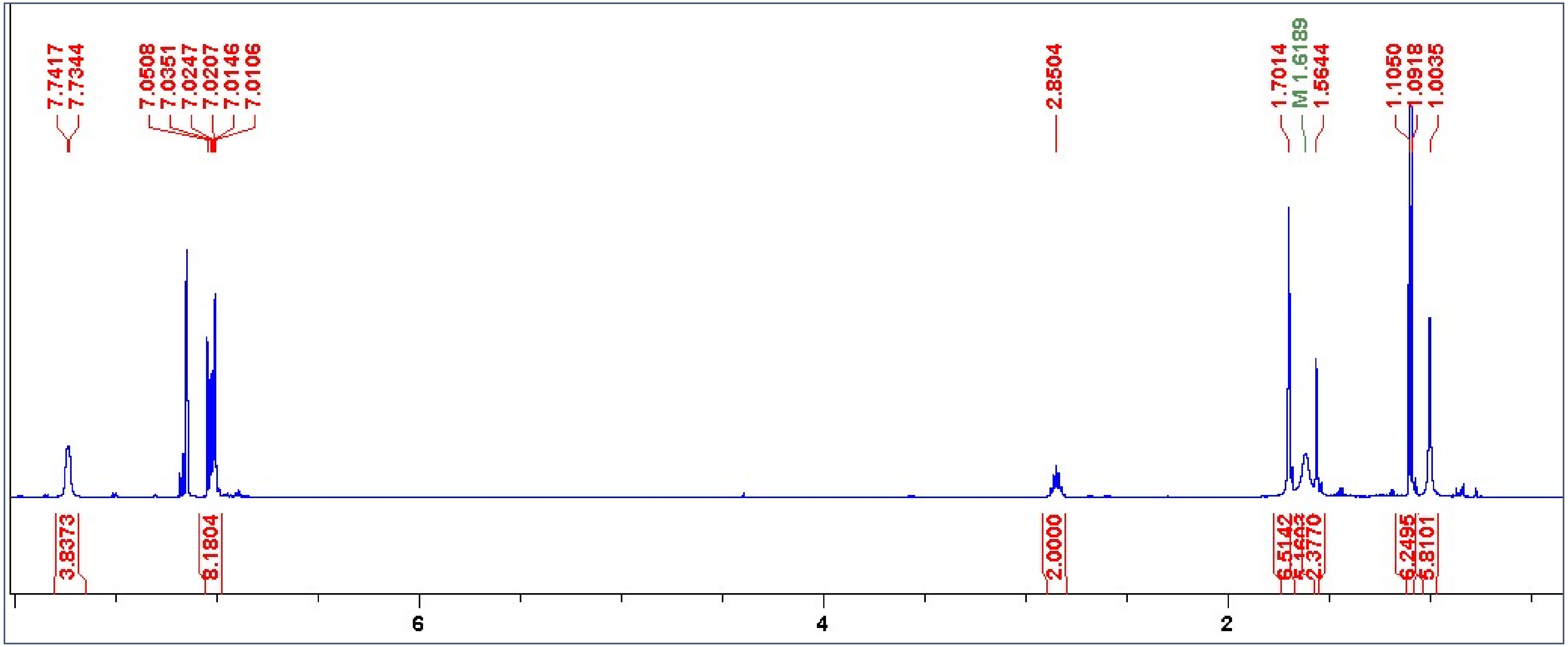

Figure S8. ^{1}H NMR spectrum of **3** in C_6D_6 at 70 °C.

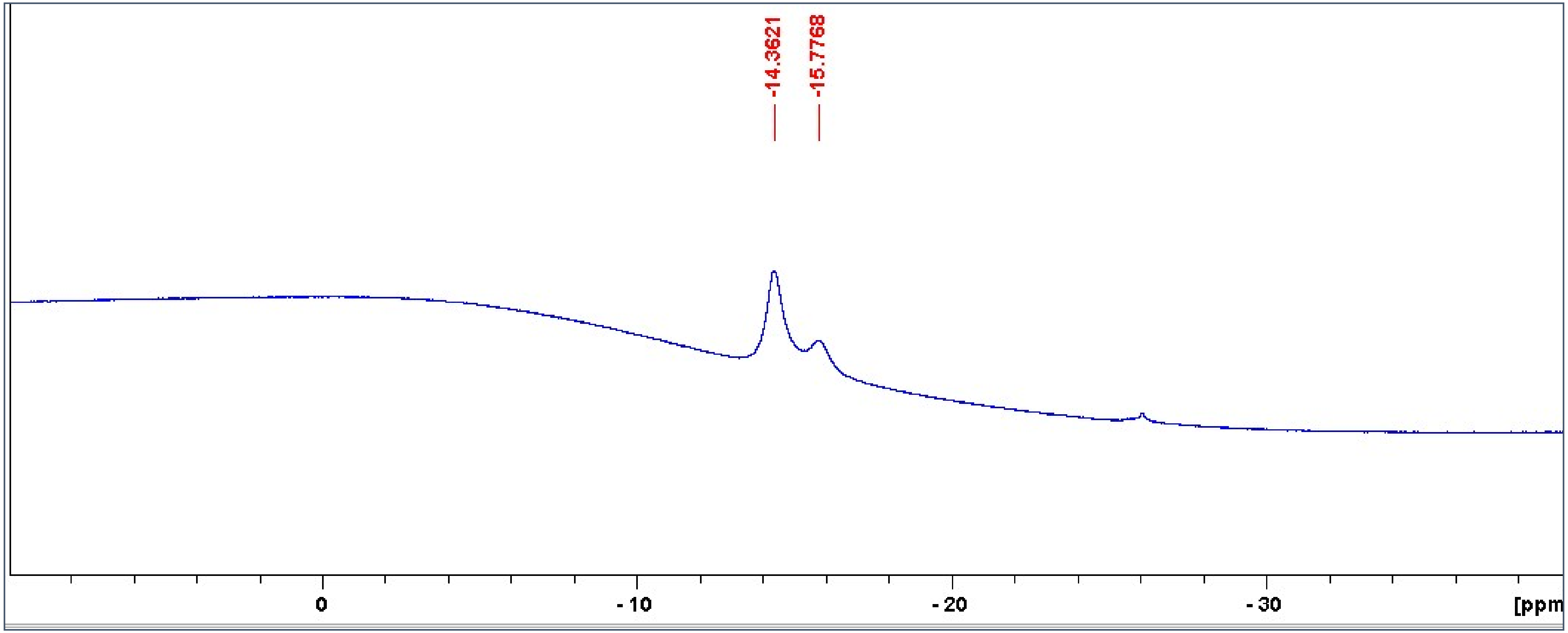

Figure S9. ^{11}B NMR spectrum of **3** (2 isomers) in C_6D_6 at rt. The small impurity at –26 ppm is the result of partial hydrolysis during isolation due to the extreme moisture-sensitivity of compound **3**.

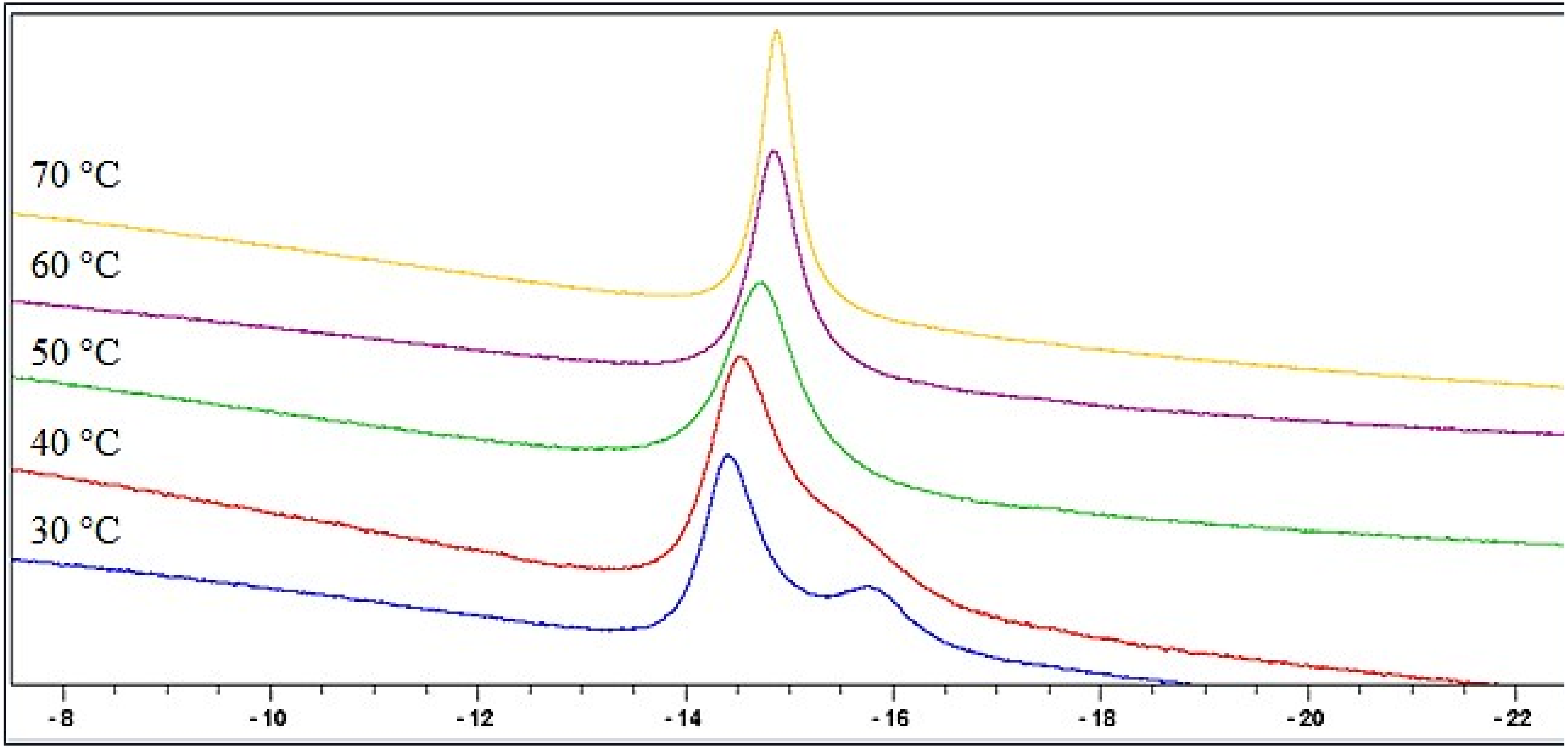

Figure S10. Variable temperature stackplot of ^{11}B NMR spectra of **3** in C_6D_6.

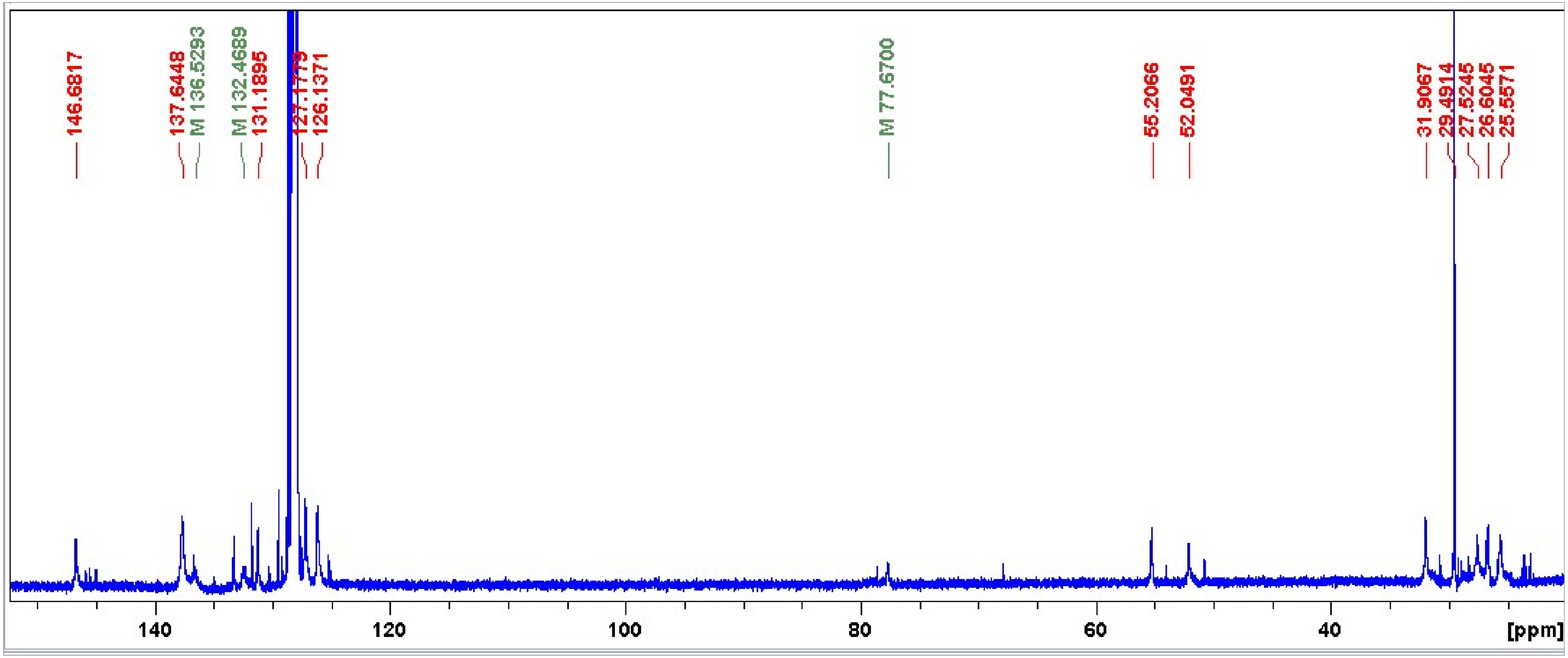

Figure S11. $^{13}C\{^1H\}$ NMR spectrum of **3** (2 isomers) in C_6D_6 at rt. The broadness of the resonances results from the fluxional behaviour of **3** in solution at room temperature.

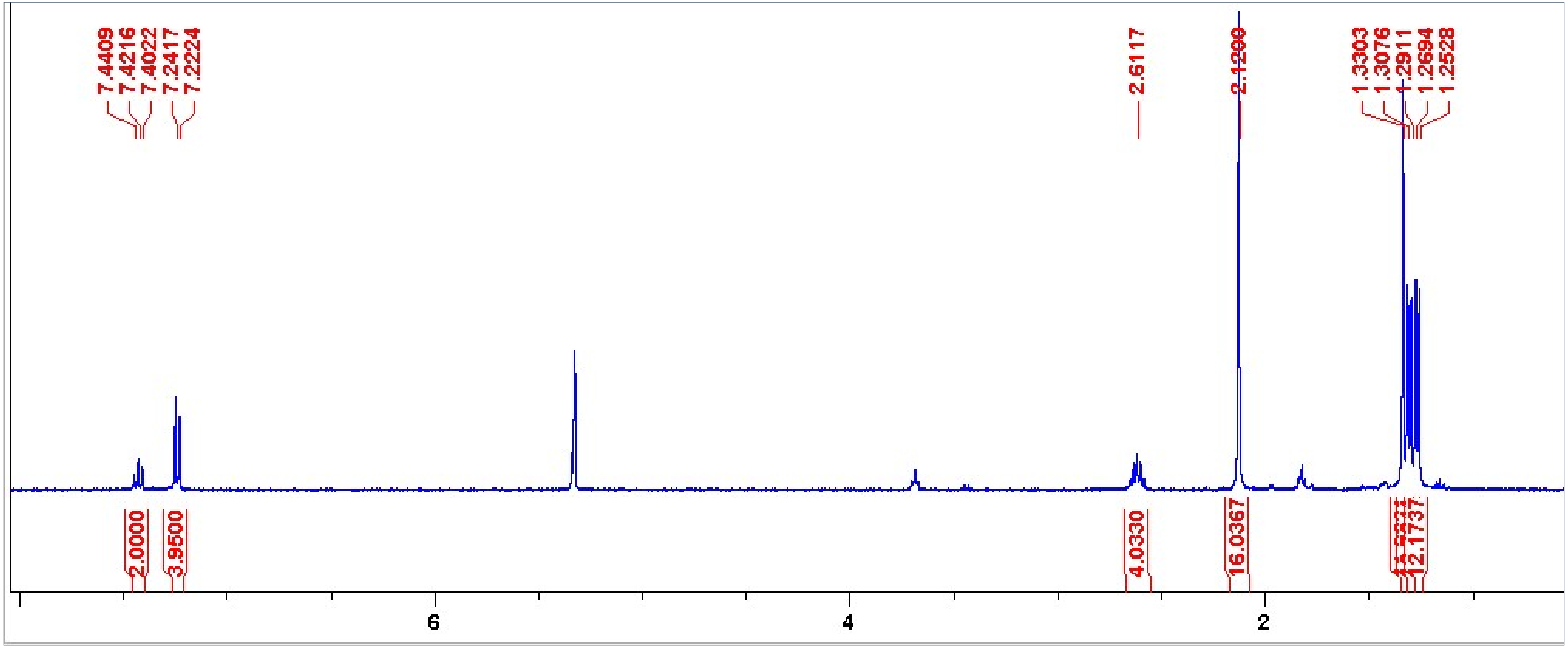

Figure S12. ^{1}H NMR spectrum of **5** (single isomer) in CD_2Cl_2 directly after dissolution. The additional multiplets at 3.69 and 1.82 ppm corrresponds to residual THF (crystallisation solvent).

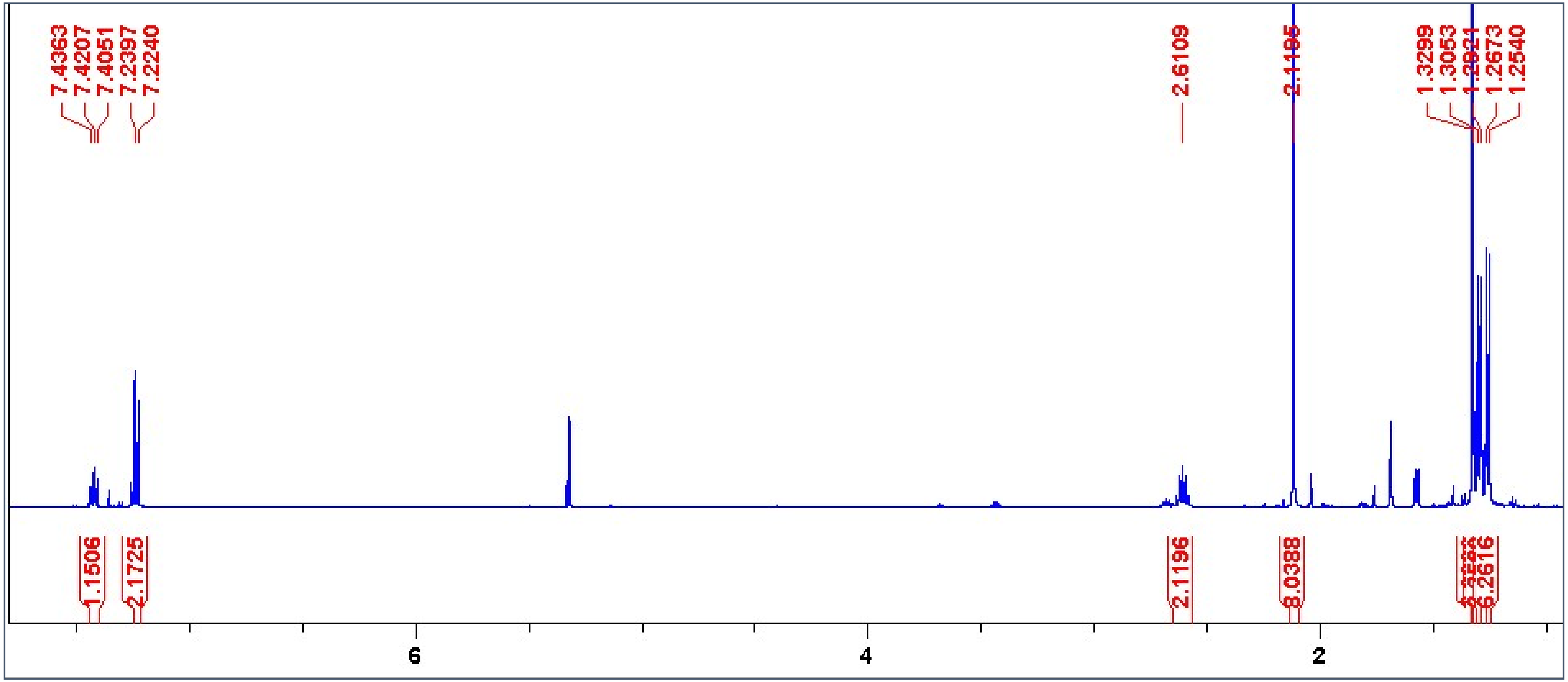

Figure S13. ^{1}H NMR spectrum of **5** (two isomers) in CD_2Cl_2 after three days at rt.

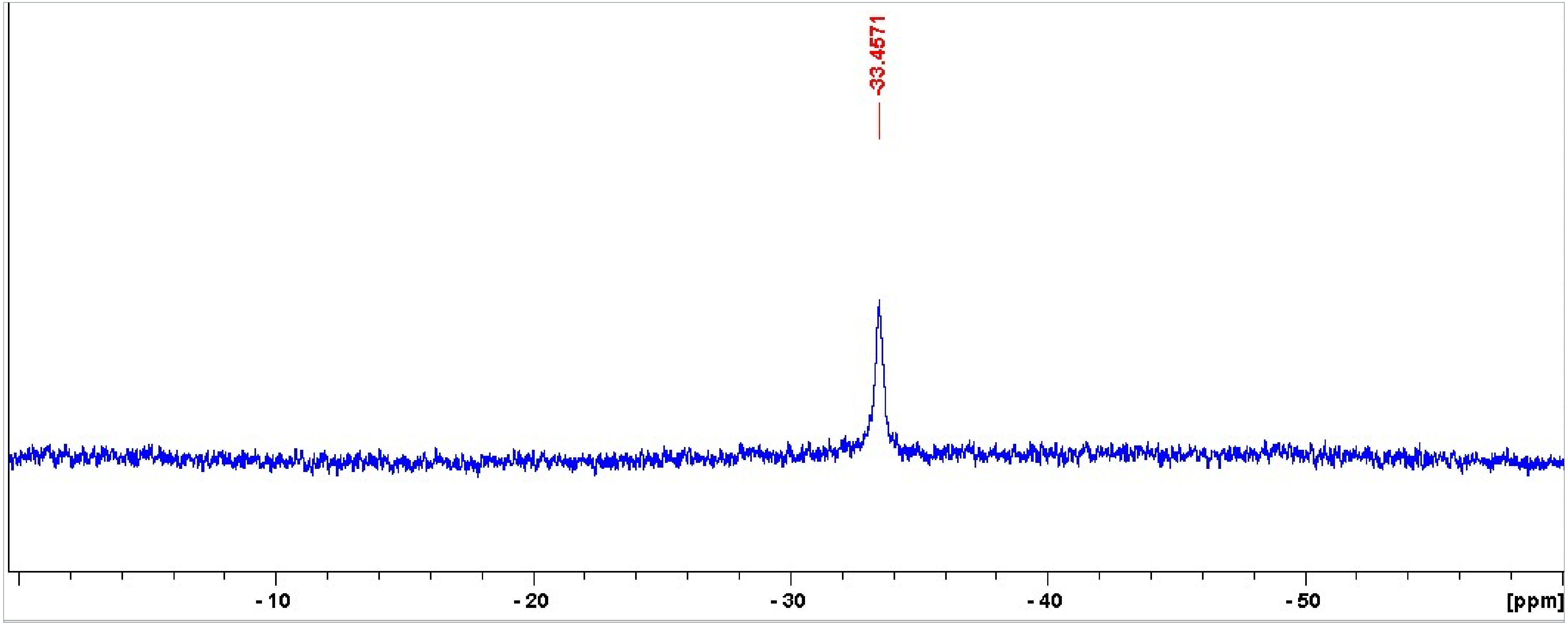

Figure S14. ^{11}B NMR spectrum of **5** in CD_2Cl_2 (single isomer) in CD_2Cl_2 directly after dissolution.

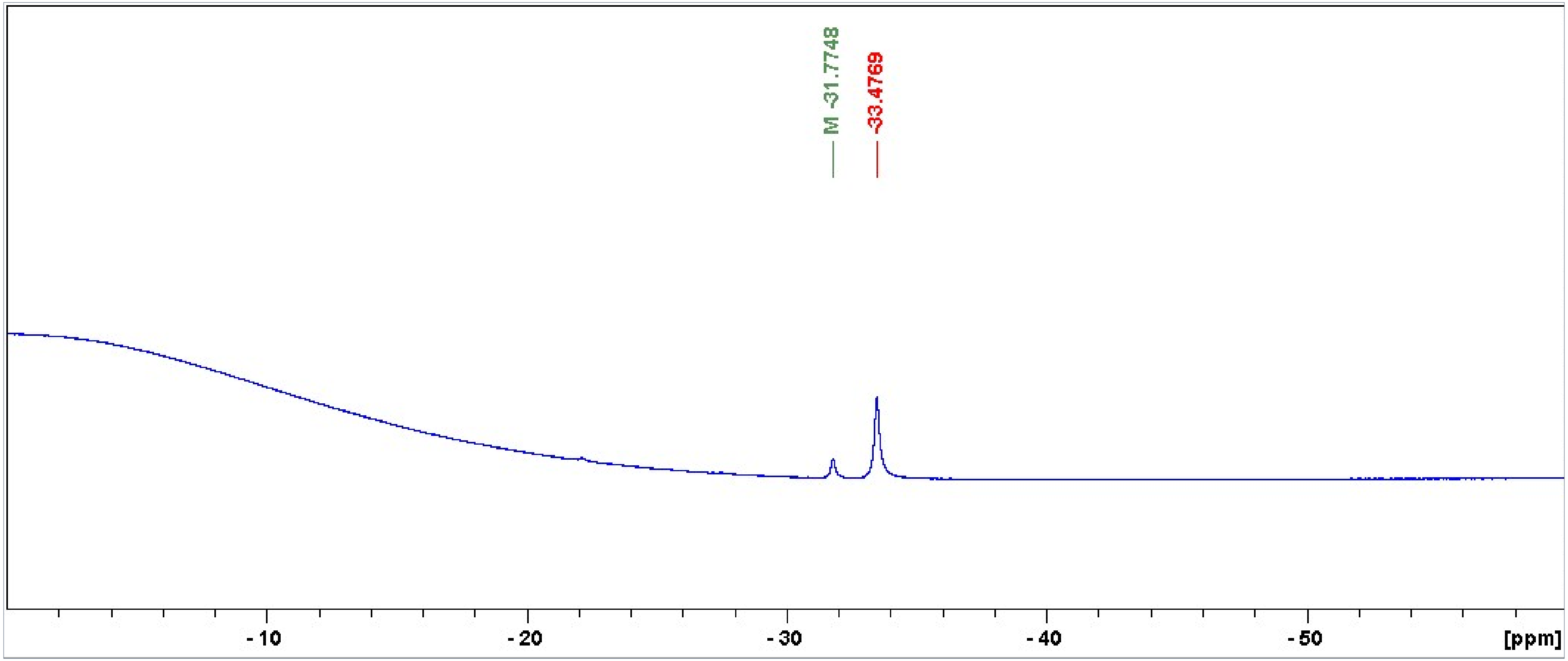

Figure S15. ^{11}B NMR spectrum of **5** (two isomers) in CD_2Cl_2 after three days at rt. The additional small resonance at ca. –22 ppm is compound **6**, which slowly forms upon decomposition of **4**.

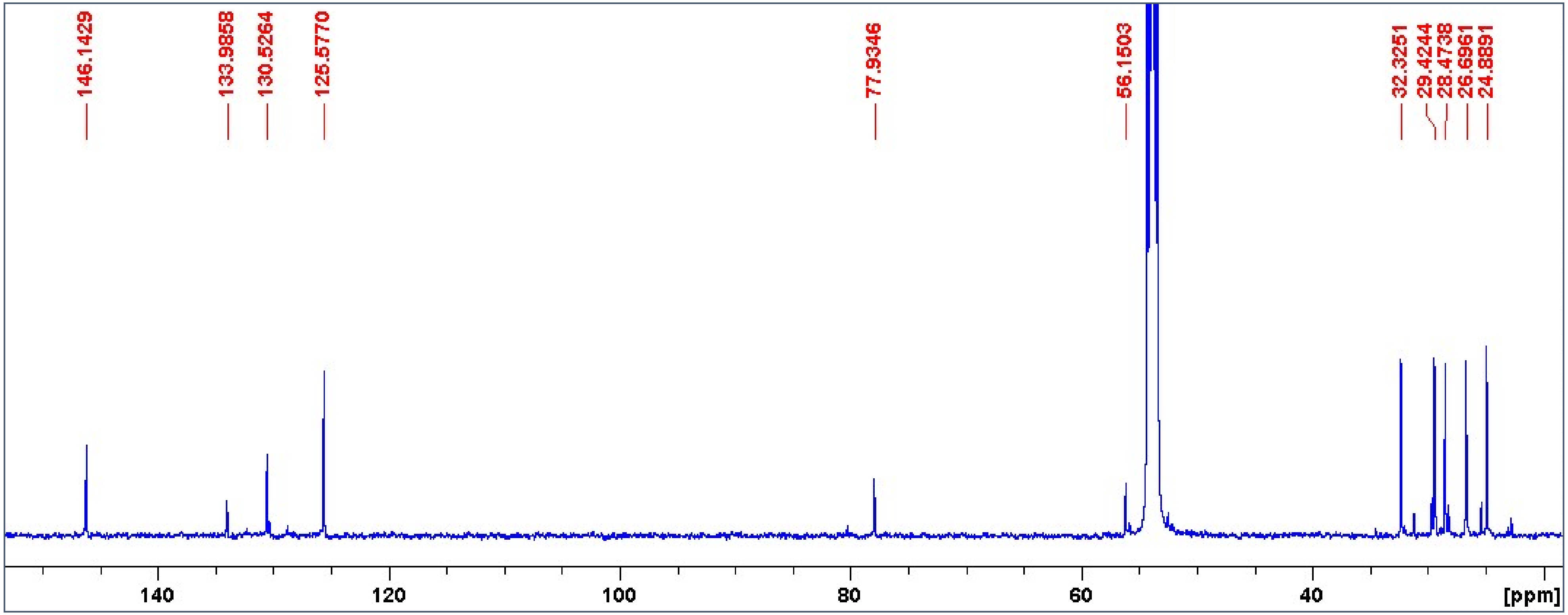

Figure S16. $^{13}C\{^{1}H\}$ NMR spectrum of **5** (two isomers) in CD_2Cl_2 after three days at rt.

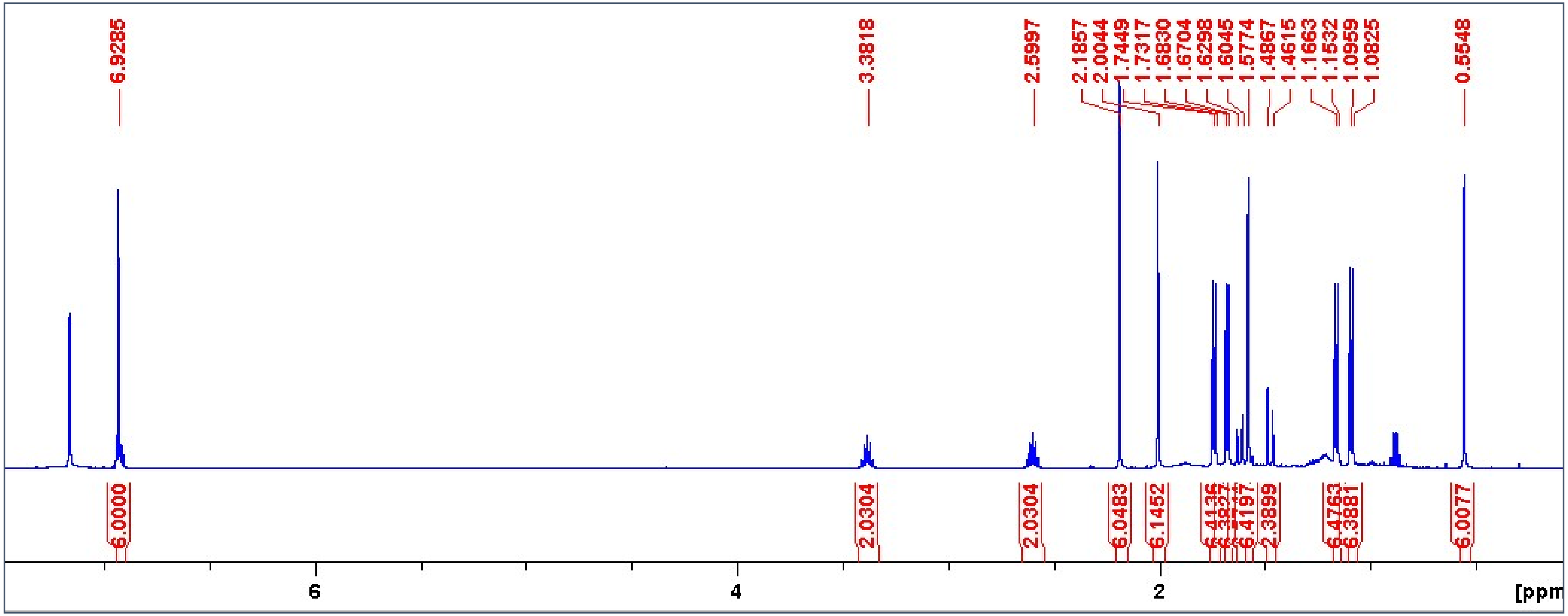

Figure S17. ^{1}H NMR spectrum of **6** in C_6D_6.

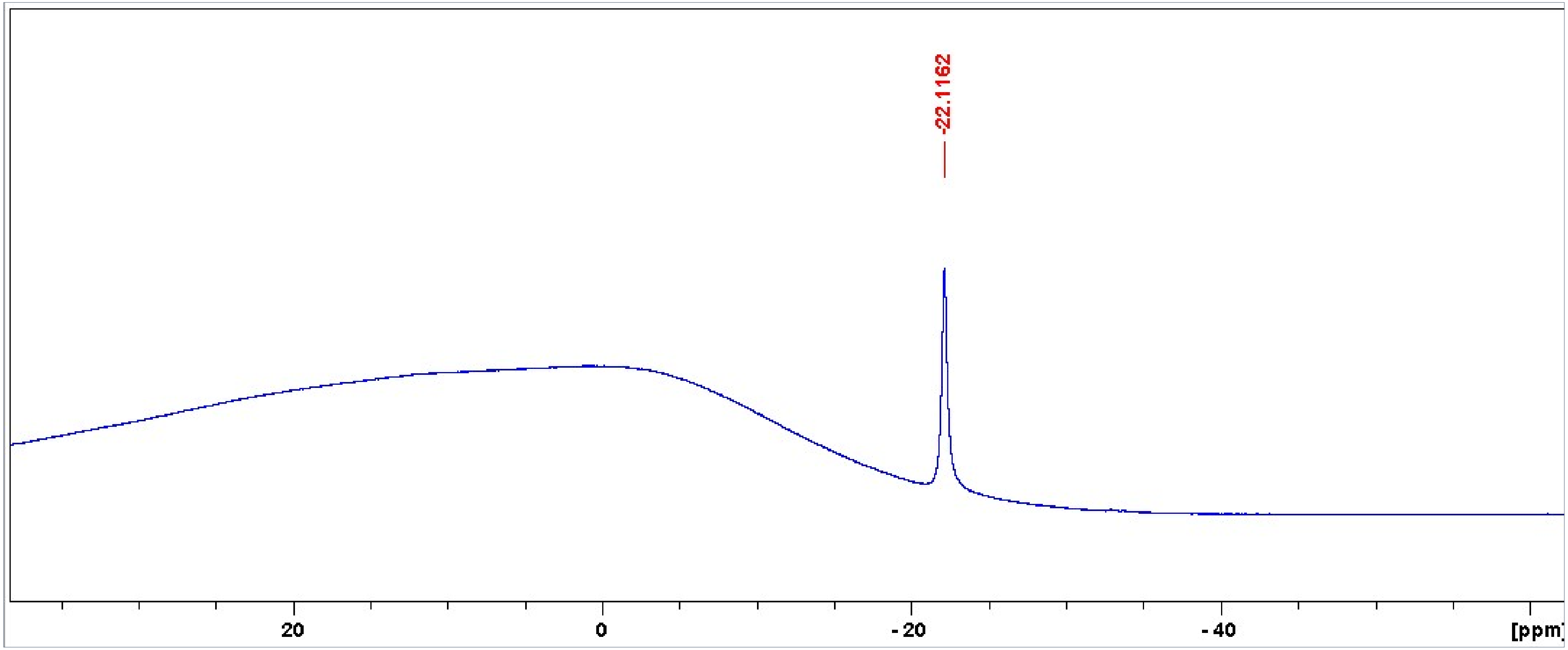

Figure S18. ^{11}B NMR spectrum of **6** in C_6D_6.

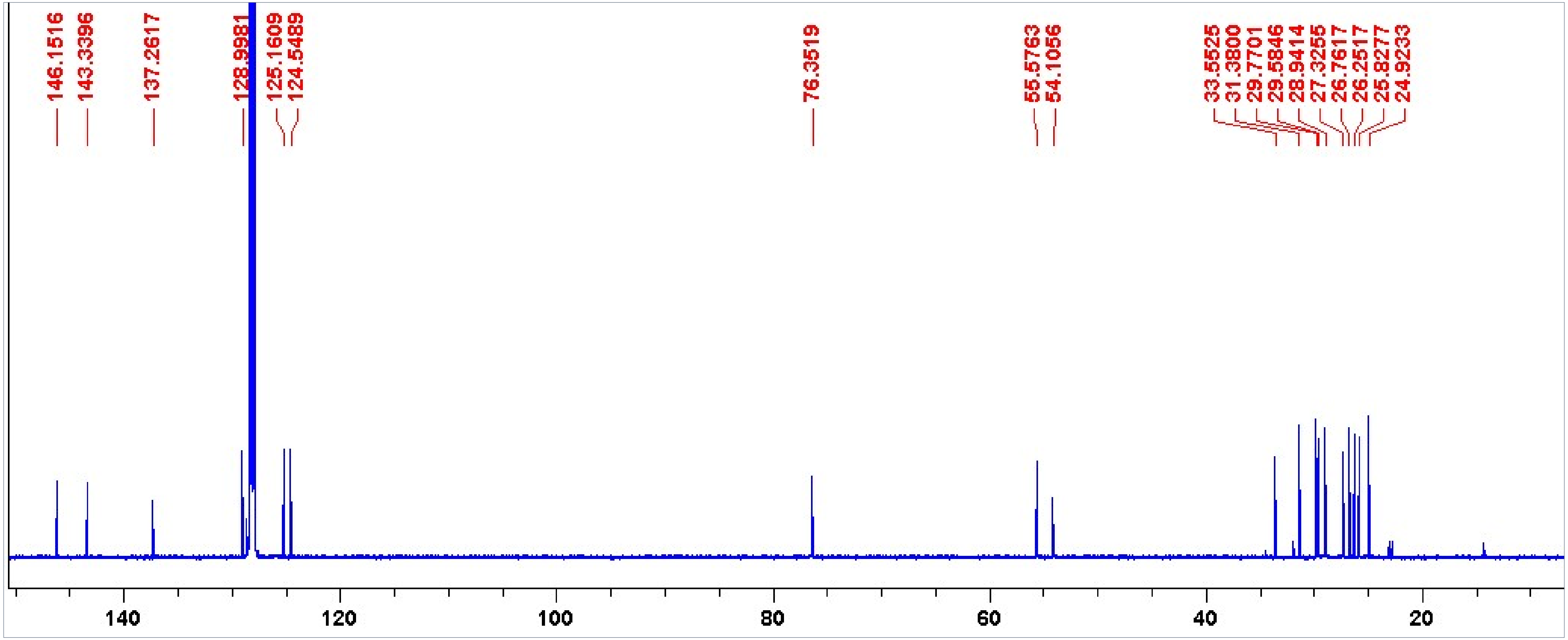

Figure S19. $^{13}C\{^{1}H\}$ NMR spectrum of **6** in C_6D_6.

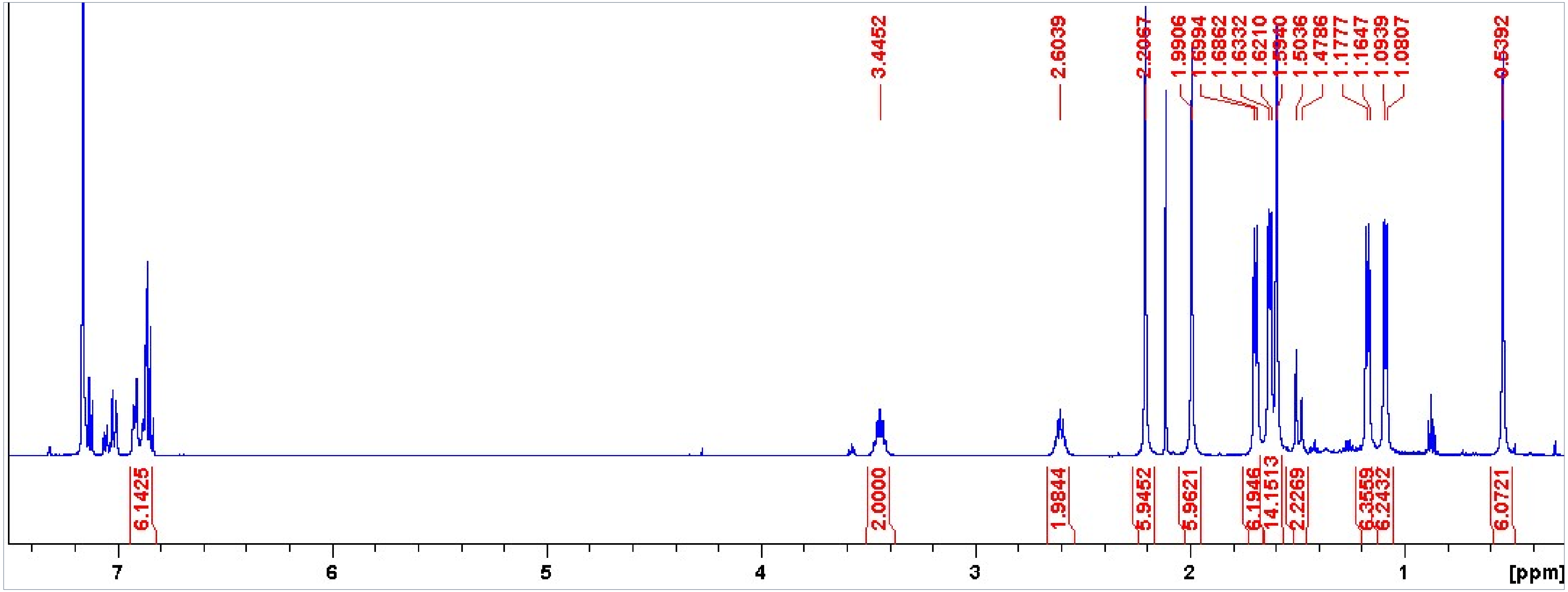

Figure S20. ^{1}H NMR spectrum of **7** in C_6D_6. The additional multiplet at 3.7 ppm corresponds to residual THF (crystallisation solvent).

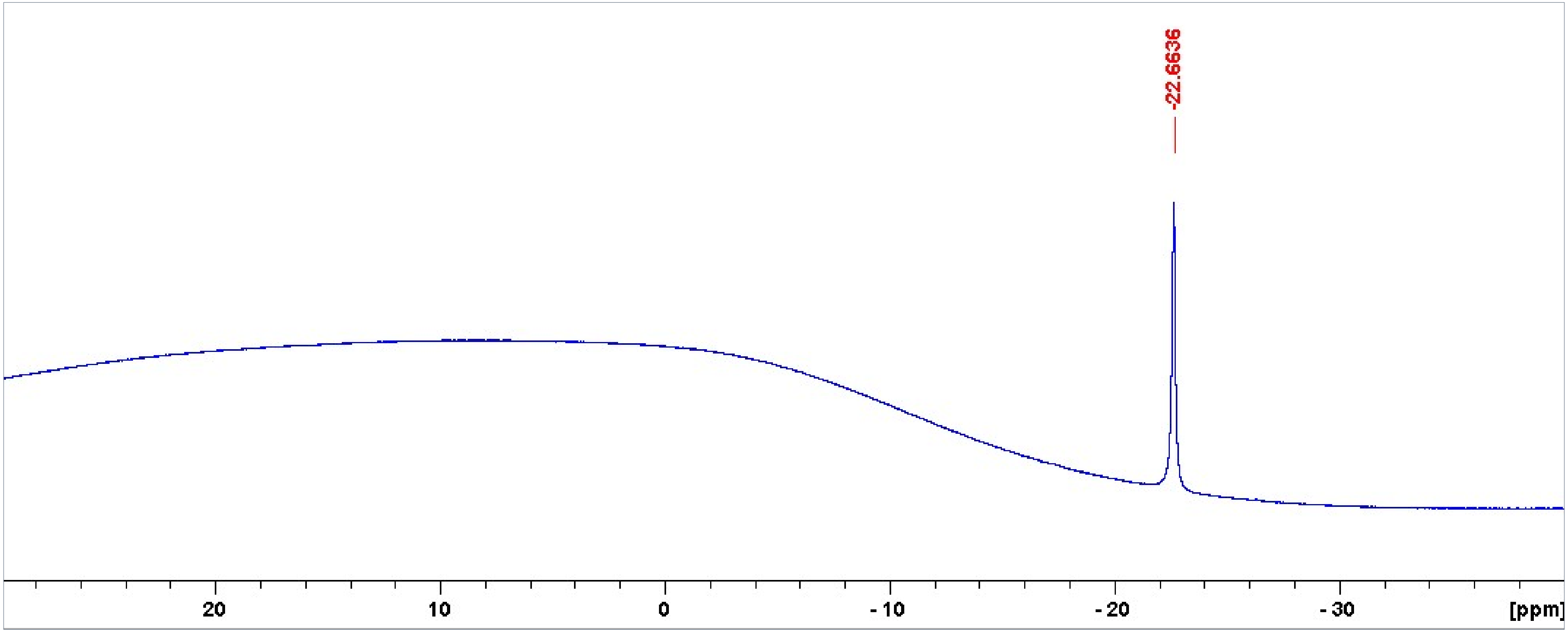

Figure S21. ^{11}B NMR spectrum of **7** in C_6D_6.

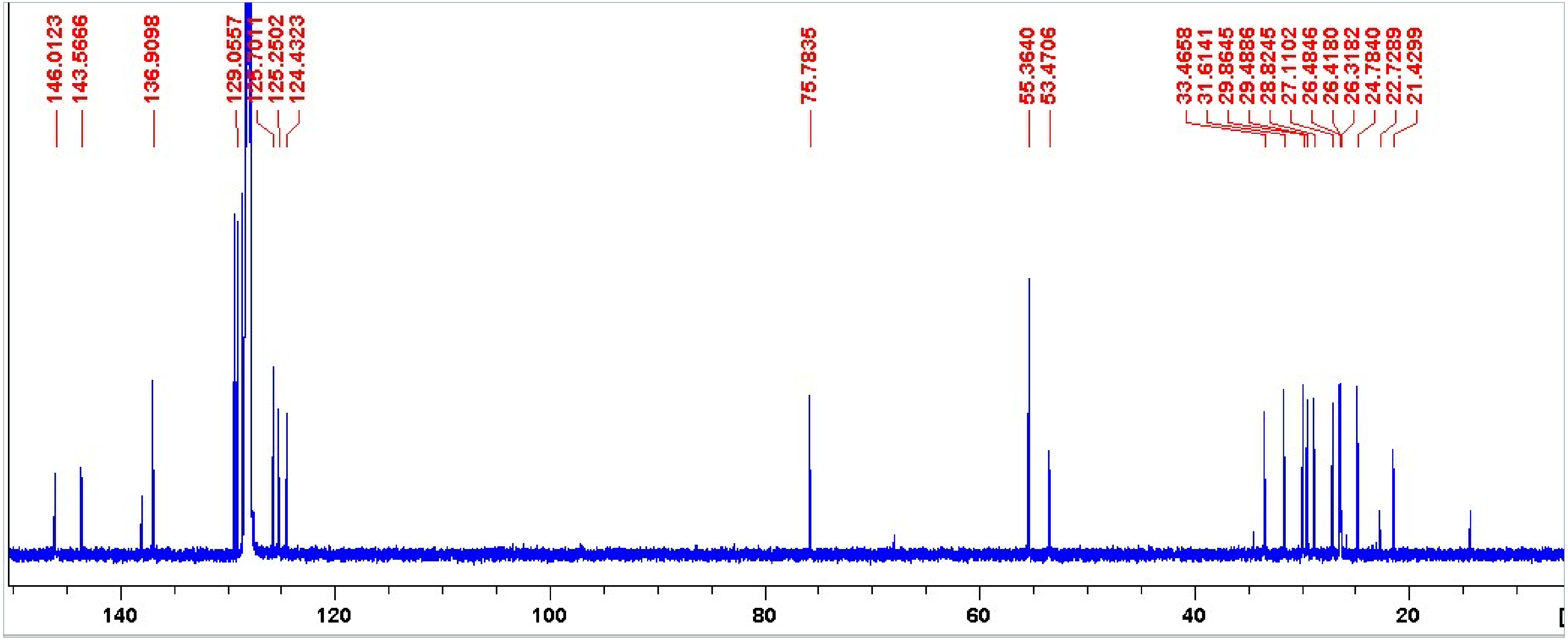

Figure S22. $^{13}C\{^1H\}$ NMR spectrum of **7** in C_6D_6.

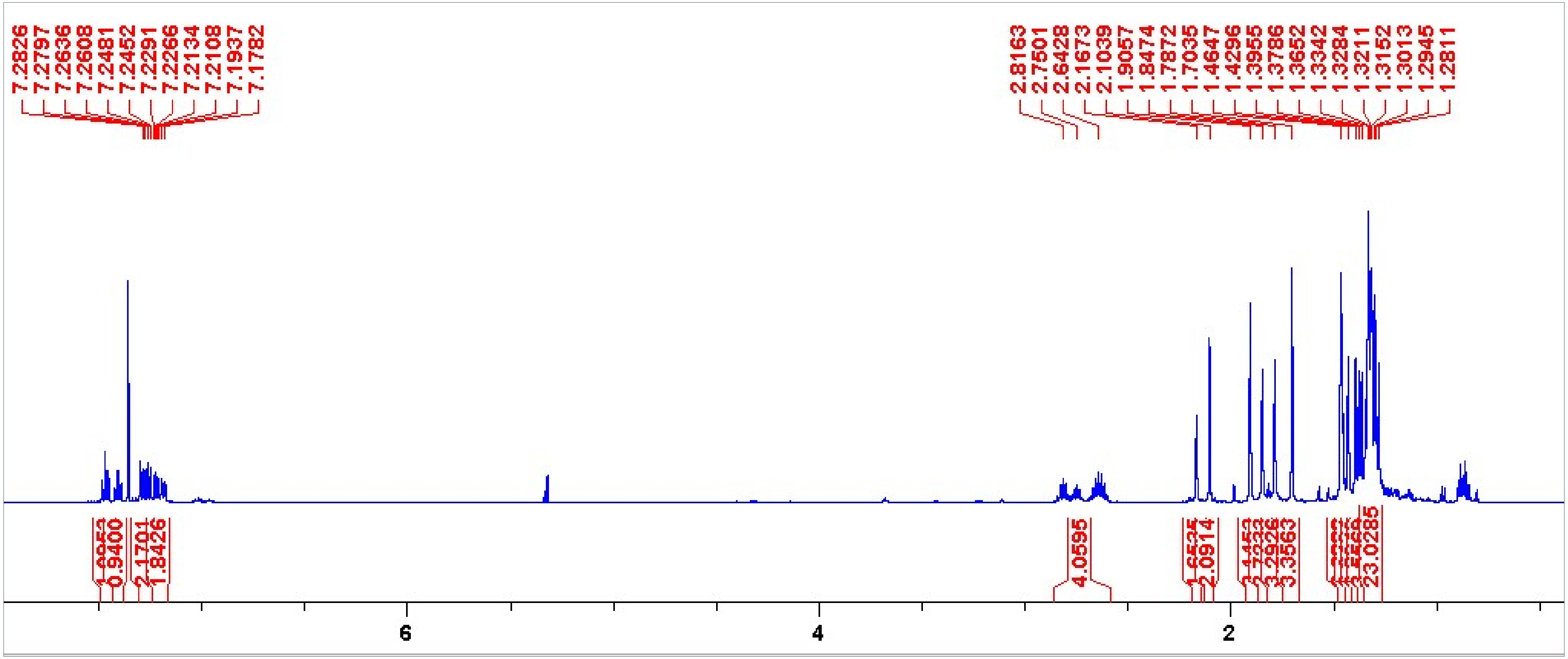

Figure S23. ^{1}H NMR spectrum of **8** (two isomers) in CD_2Cl_2.

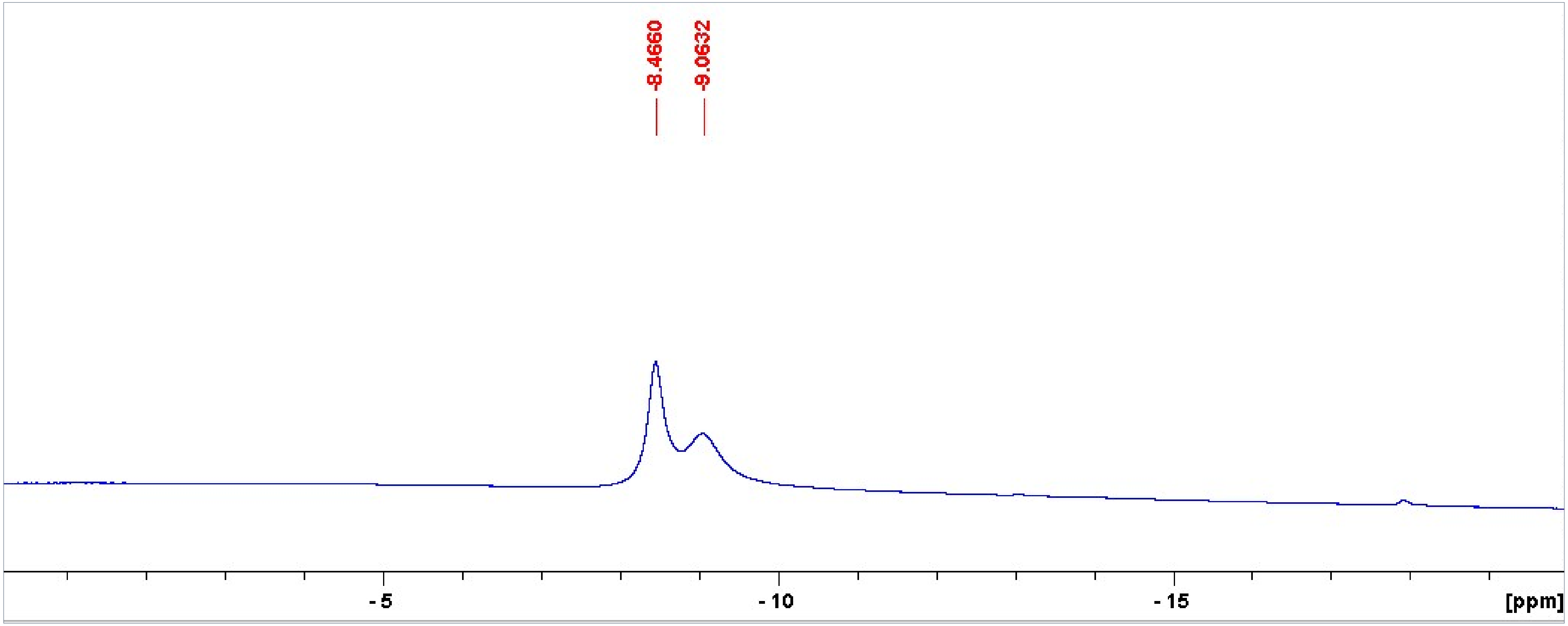

Figure S24. ^{11}B NMR spectrum of **8** (two isomers) in CD_2Cl_2.

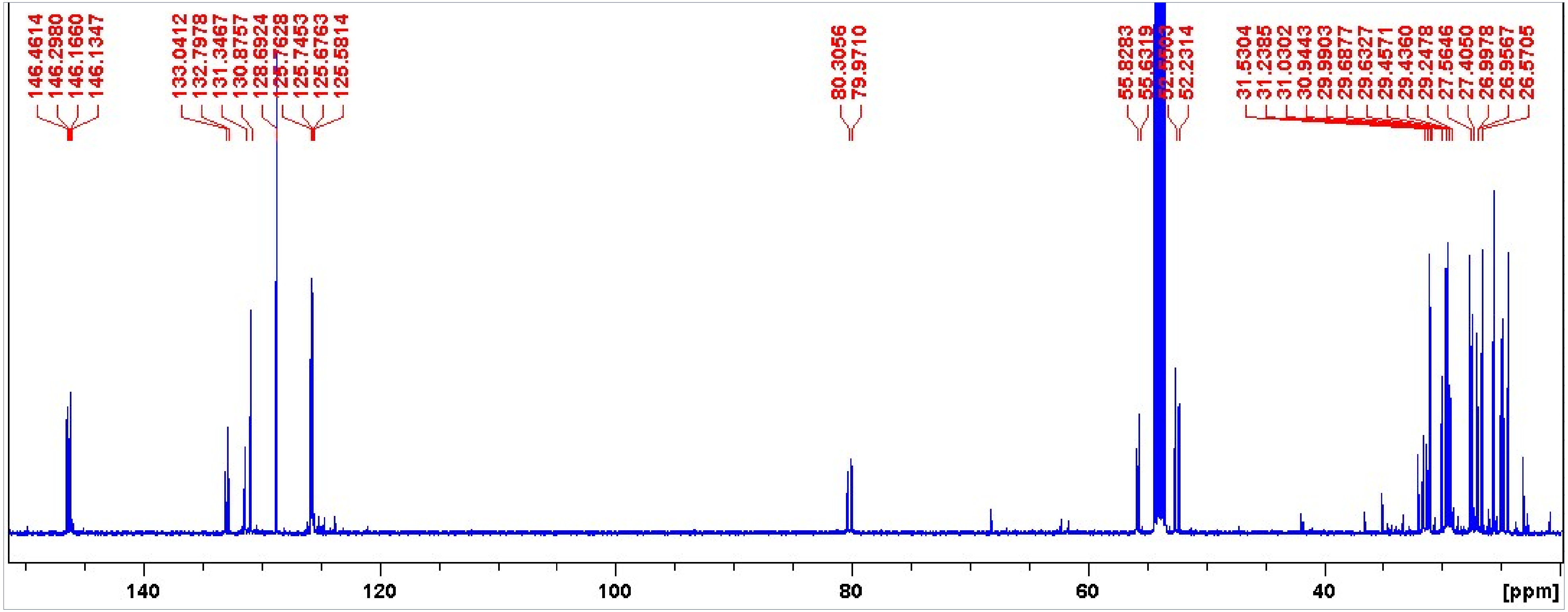

Figure S25. $^{13}C\{^{1}H\}$ NMR spectrum of **8** (two isomers) in CD_2Cl_2.

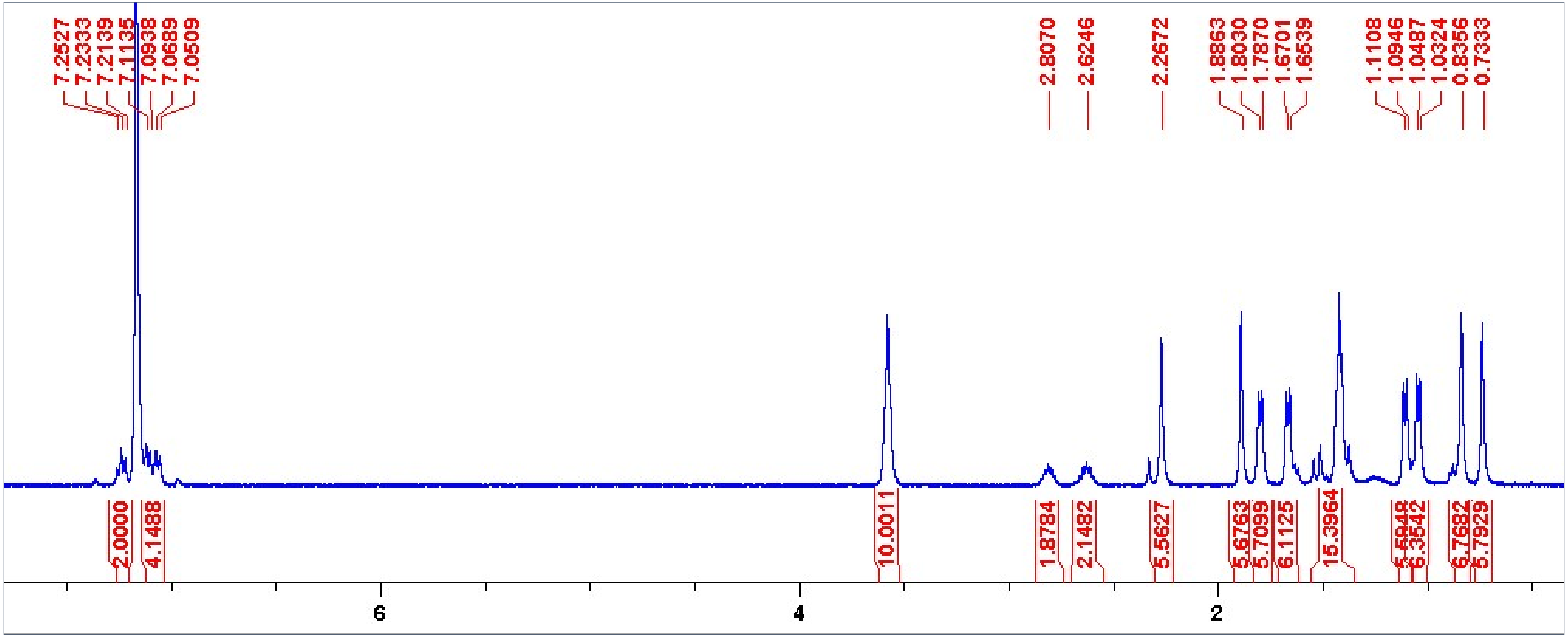

Figure S26. ^{1}H NMR spectrum of **9** in C_6D_6. Additional resonances at 3.6 and 1.4 ppm correspond to residual THF (crystallisation solvent).

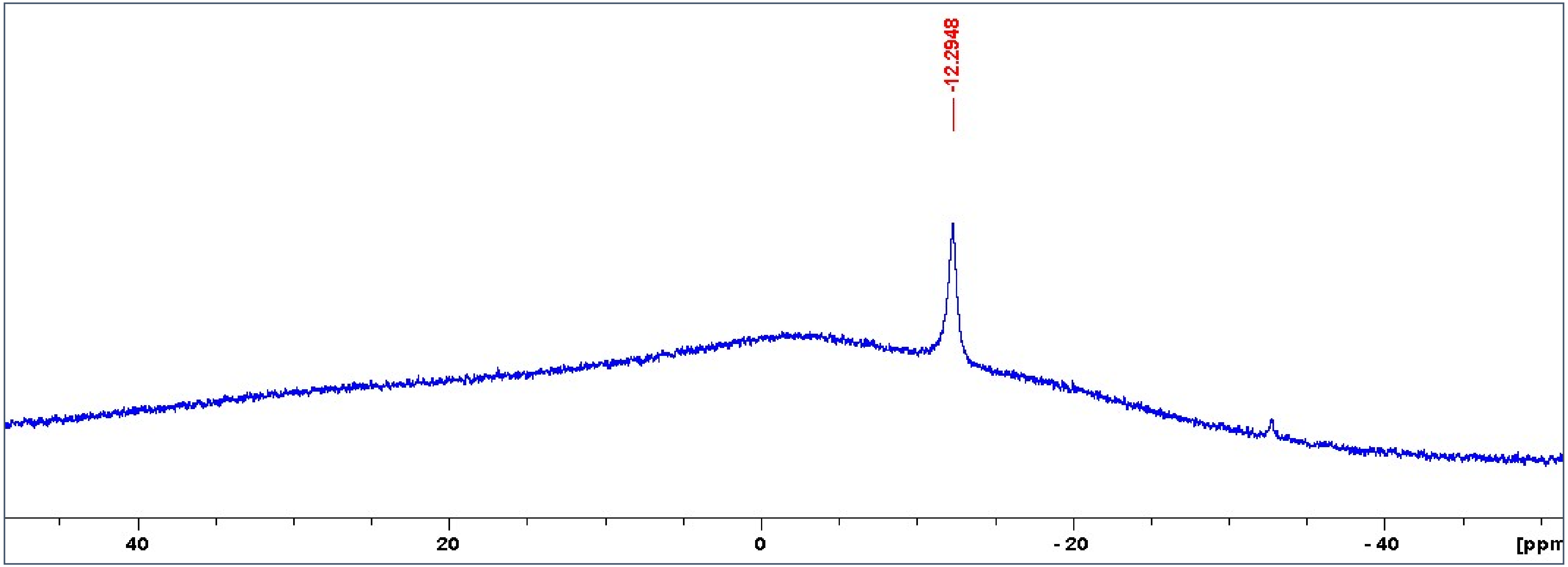

Figure S27. ^{11}B NMR spectrum of **9** in C_6D_6. The small impurity at –33 ppm is the minor reaction product **5**, which invariably co-crystallised in small amounts with **9**.

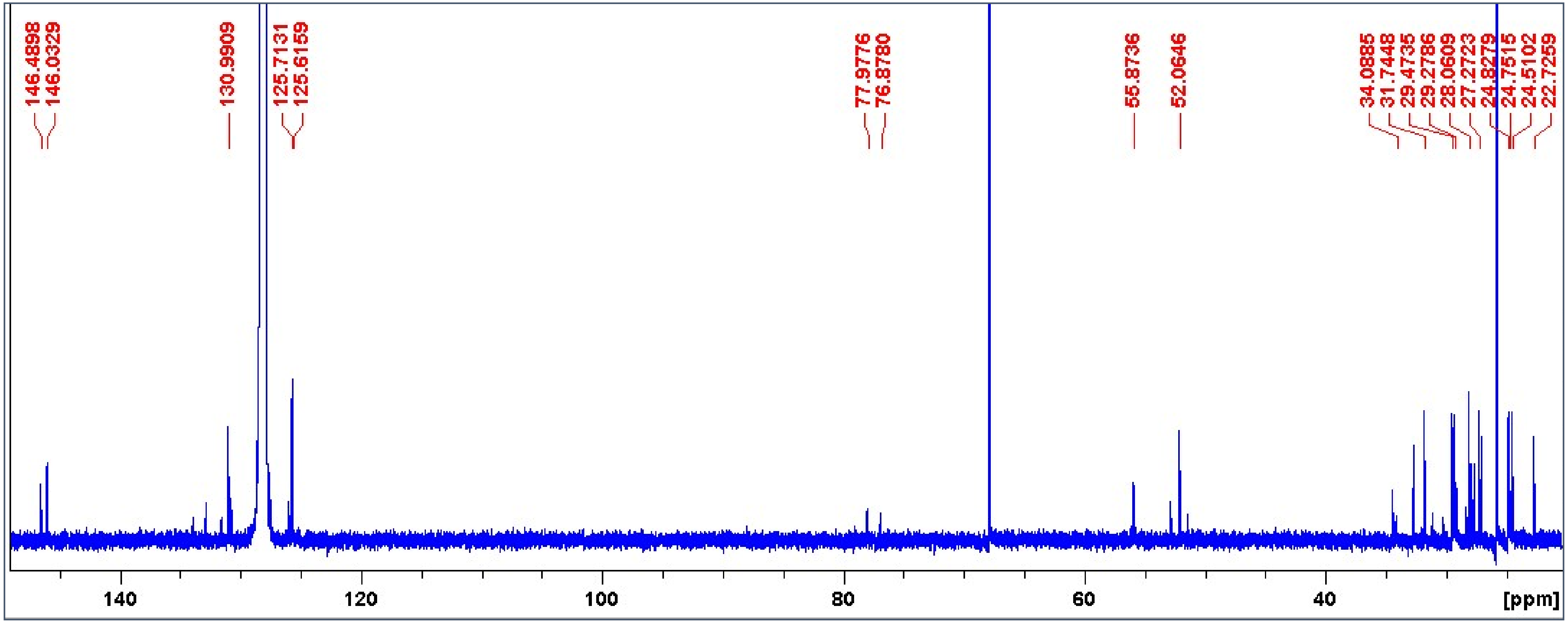

Figure S28. $^{13}C\{^{1}H\}$ NMR spectrum of **9** in C_6D_6. Additional resonances at 68 and 26 ppm correspond to residual THF.

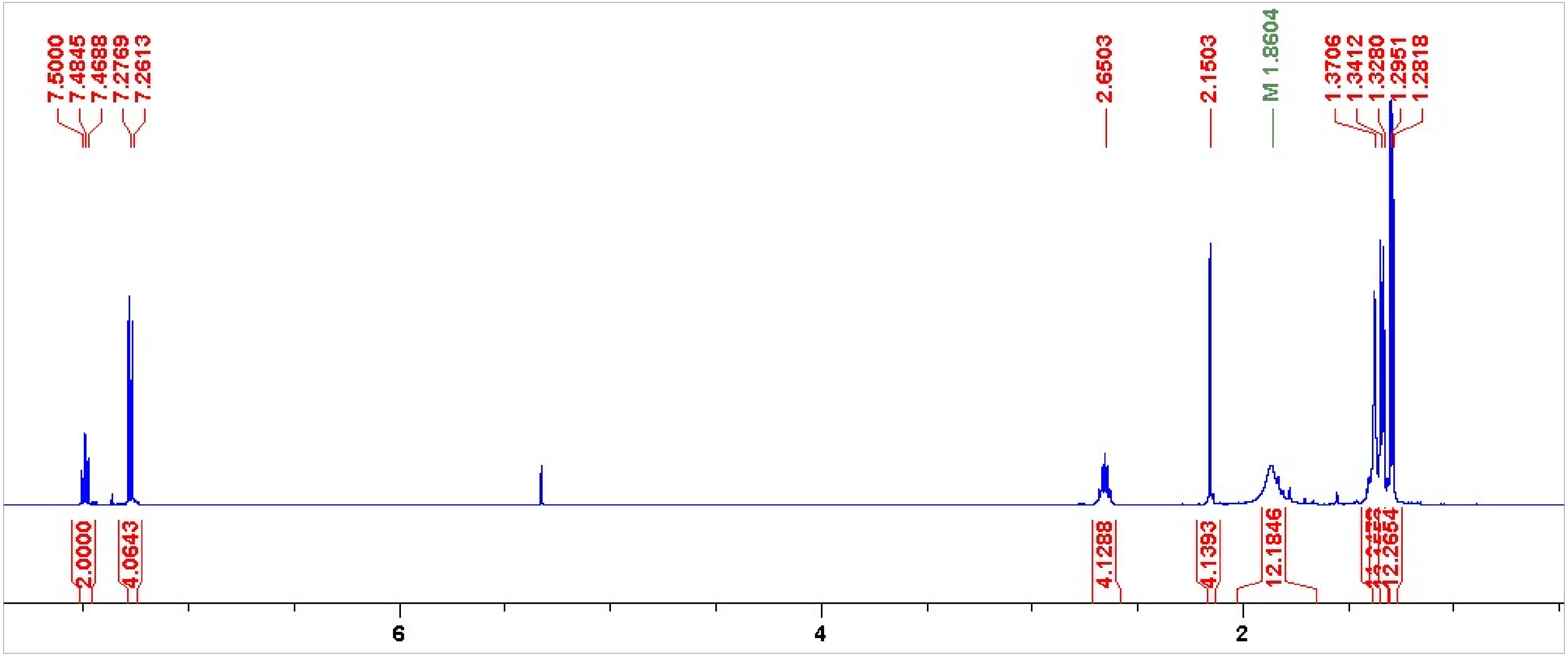

Figure S29. ^{1}H NMR spectrum of **10** in CD_2Cl_2.

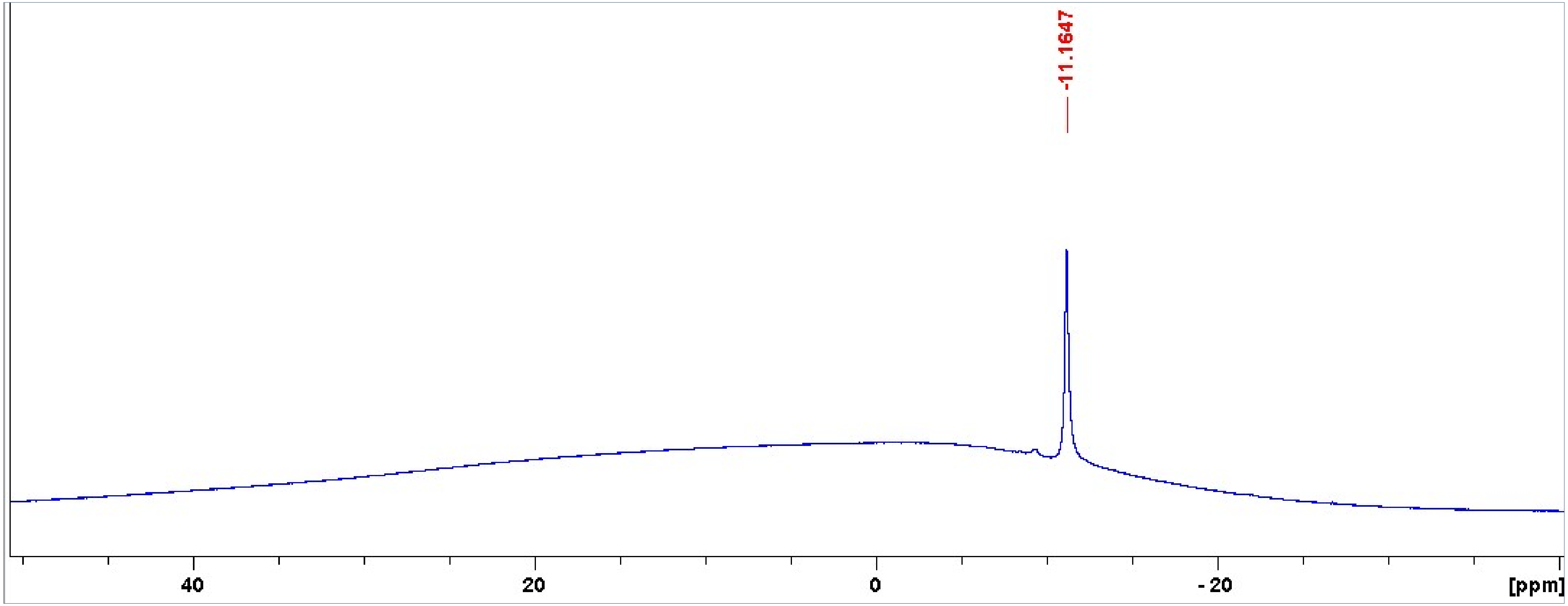

Figure S30. ^{11}B NMR spectrum of **10** in CD_2Cl_2. The small impurity at –9 ppm is the minor reaction product **8**, which invariably co-crystallised in small amounts with **10**.

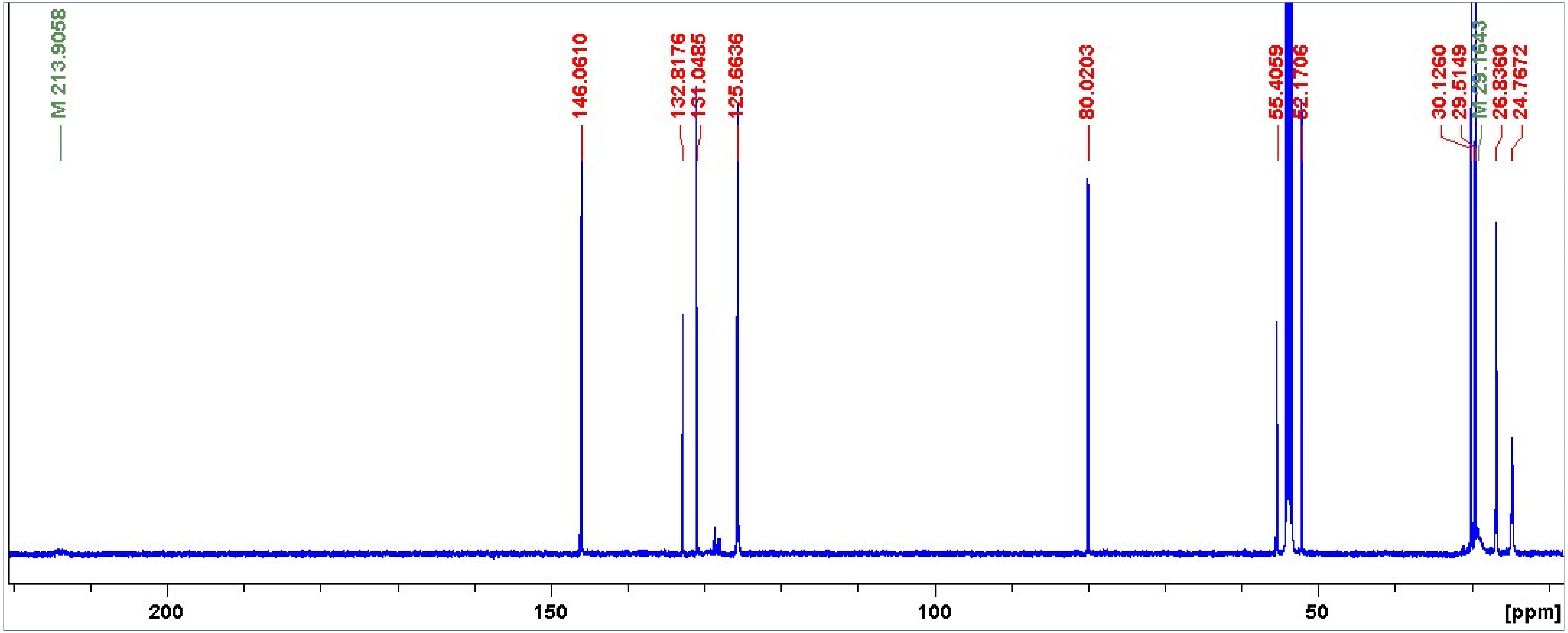

Figure S31. $^{13}C\{^1H\}$ NMR spectrum of **10** in CD_2Cl_2.

Mass Spectrometry

Mass specta were acquired on an Exactive Plus mass spectrometer equipped with a Thermo Scientific Orbitrap detector.

Atmopheric Solids Analysis Probe (ASAP)-MS was carried out on crystalline samples using an Atmospheric Pressure Chemical Ionisation (APCI) source with corona needle and auxiliary gas temperature at 250 °C, except when stated otherwise.

Liquid Injection Field Desorption/Ionization (LIFDI)-MS was carried out on toluene solutions of the compounds using a Linden CMS LIFDI 700 ion source, with the actual measurement carried out on the Exactive Plus instrument. The voltage of the LIFDI emitter was 10 kV, the acceleration voltage 5 V and the heating current was ramped up to 100 mA at a rate of 30 $mA \cdot min^{-1}$.

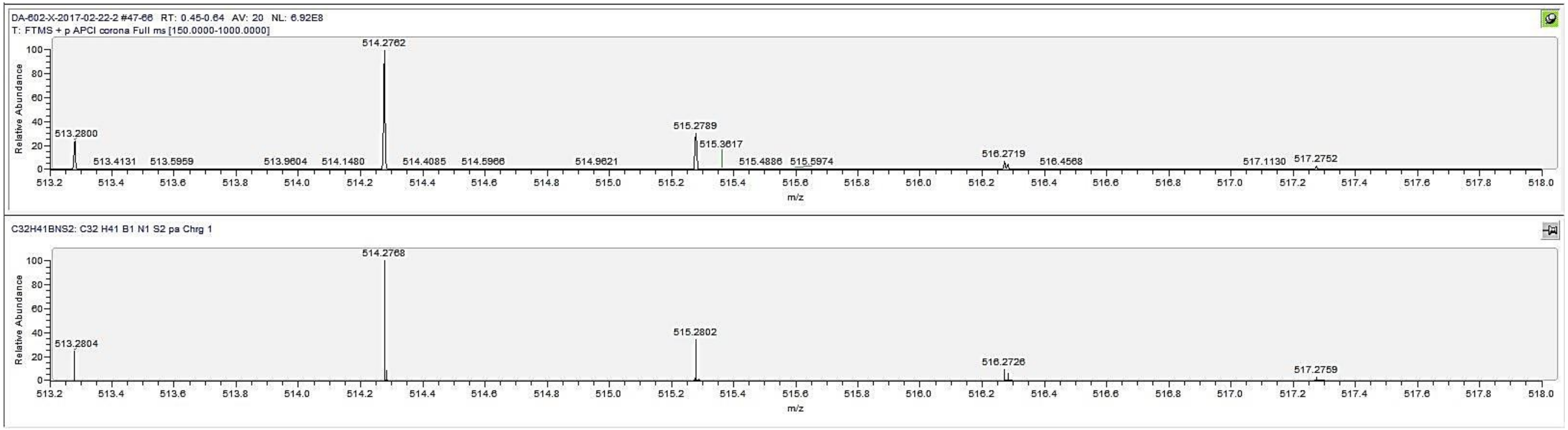

Figure S32. Measured (top) and calculated (bottom) HRMS isotopic distribution pattern for $[C_{32}H_{41}BNS_2]$ = [**1**–CN] (LIFDI-MS).

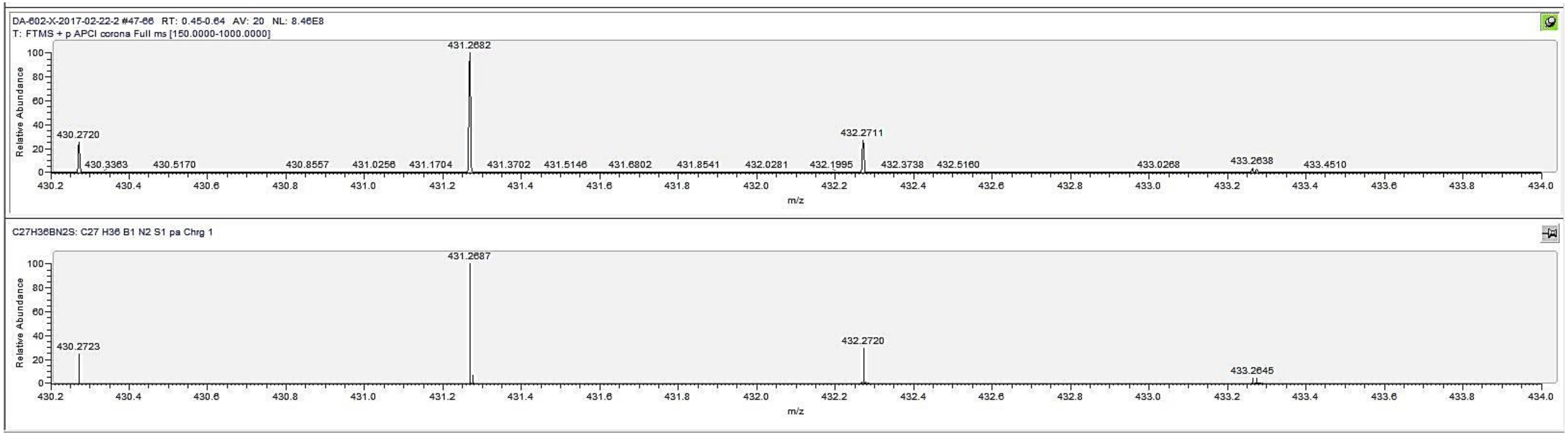

Figure S33. Measured (top) and calculated (bottom) HRMS isotopic distribution pattern for $[C_{27}H_{36}BN_2S]$ = [**1**–SPh] (LIFDI-MS).

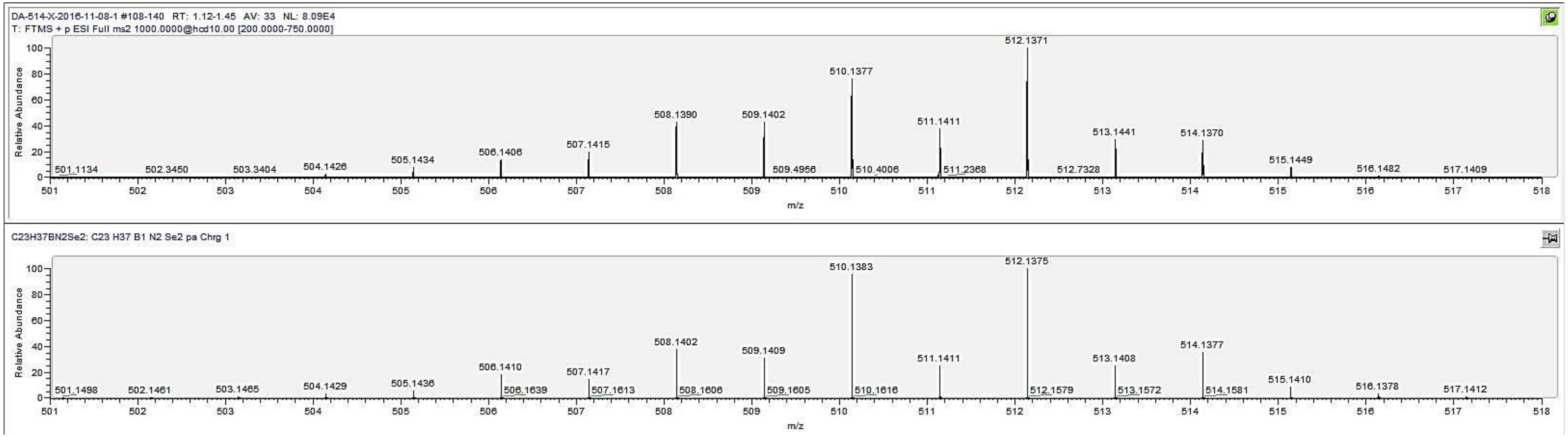

Figure S34. Measured (top) and calculated (bottom) HRMS isotopic distribution pattern for [$C_{23}H_{37}B_1N_2Se_2$] = [**2**] (LIFDI-MS).

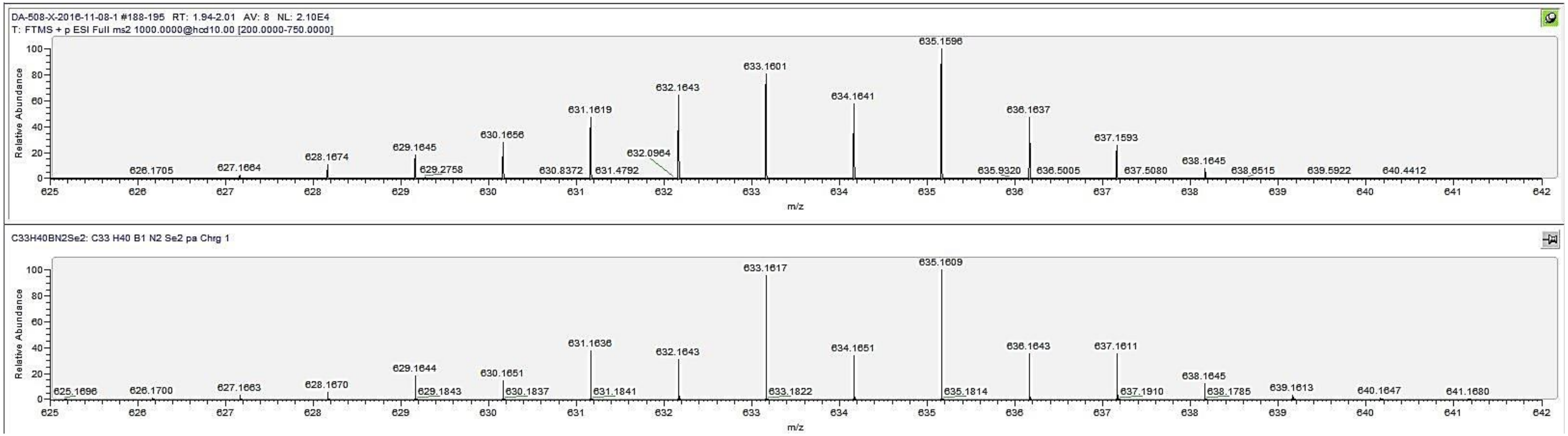

Figure S35. Measured (top) and calculated (bottom) HRMS isotopic distribution pattern for [$C_{33}H_{40}B_1N_2Se_2$] = [**3**] (LIFDI-MS).

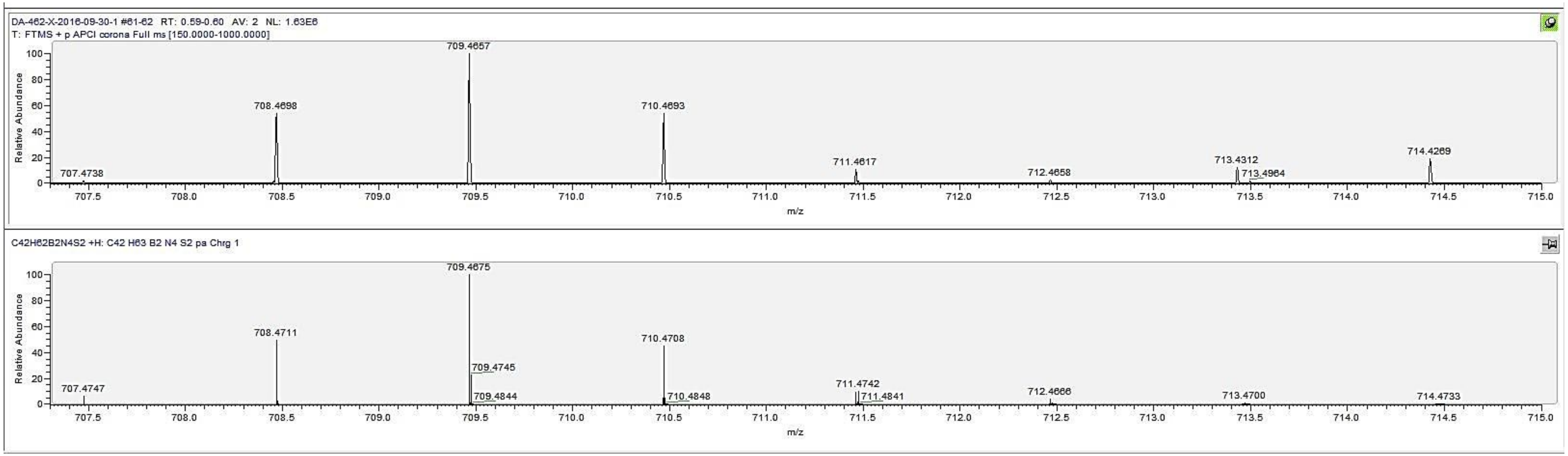

Figure S36. Measured (top) and calculated (bottom) HRMS isotopic distribution pattern for $[C_{42}H_{63}B_2N_4S_2]^+$ = [**4**+H]$^+$ (ASAP positive-MS).

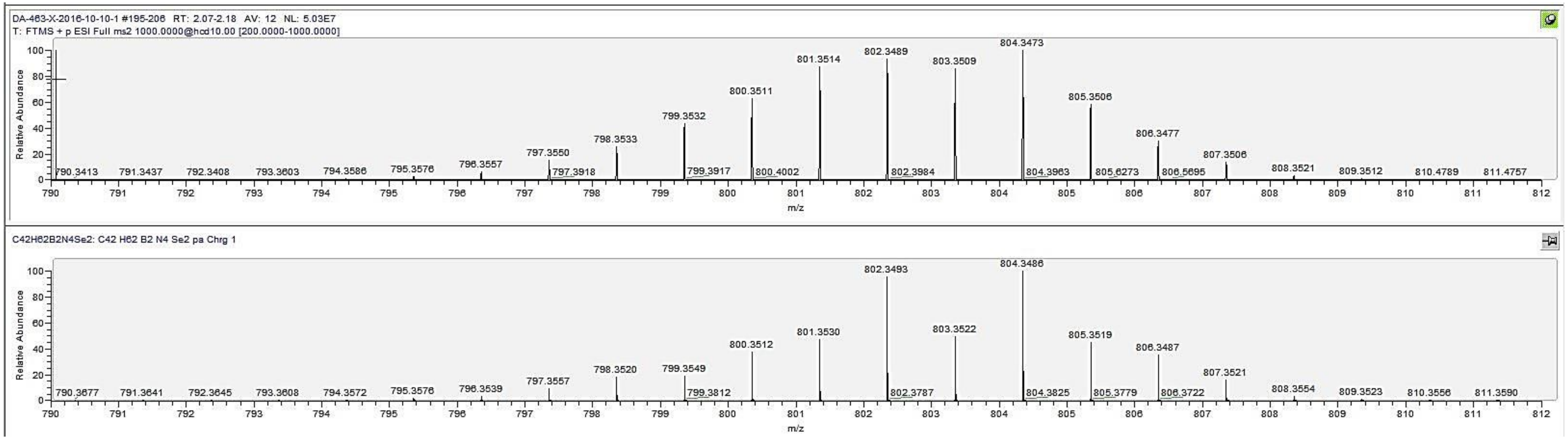

Figure S37. Measured (top) and calculated (bottom) HRMS isotopic distribution pattern for $[C_{42}H_{63}B_2N_4Se_2]^+$ = [**5**+H]$^+$ (LIFDI).

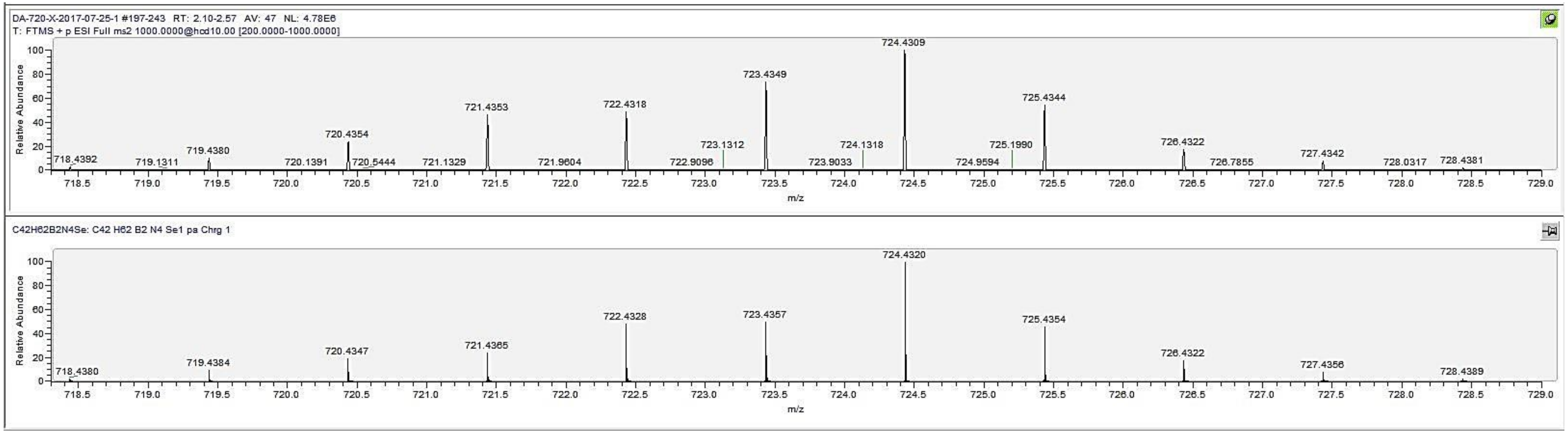

Figure S38. Measured (top) and calculated (bottom) HRMS isotopic distribution pattern for [$C_{42}H_{62}B_2N_4Se$] = [**6**] (LFDI-MS).

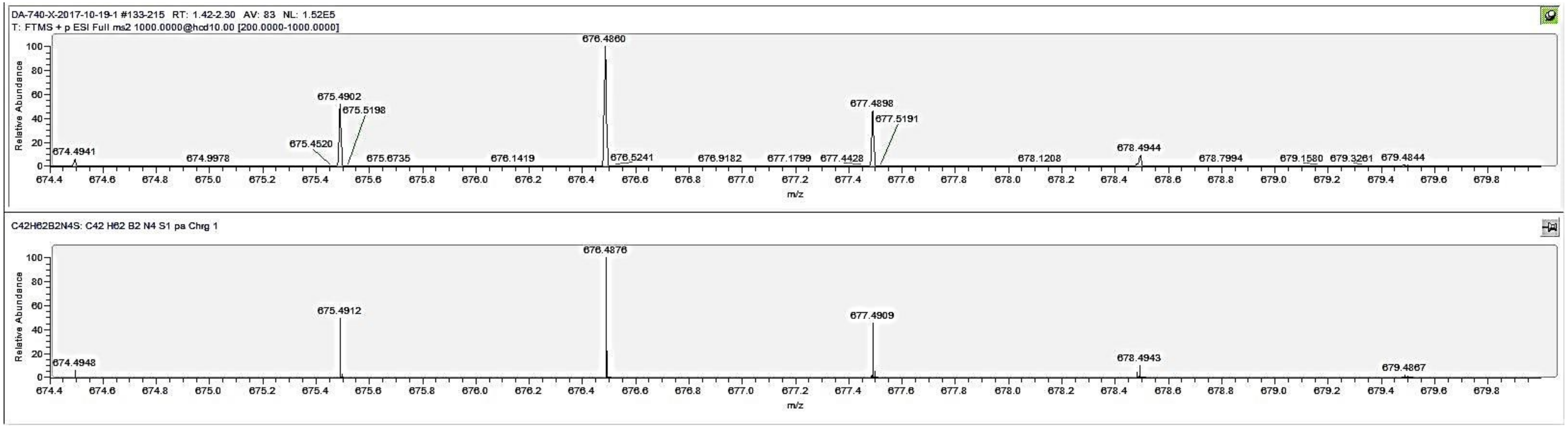

Figure S39. Measured (top) and calculated (bottom) HRMS isotopic distribution pattern for [$C_{42}H_{62}B_2N_4S$] = [**7**] (LIFDI-MS).

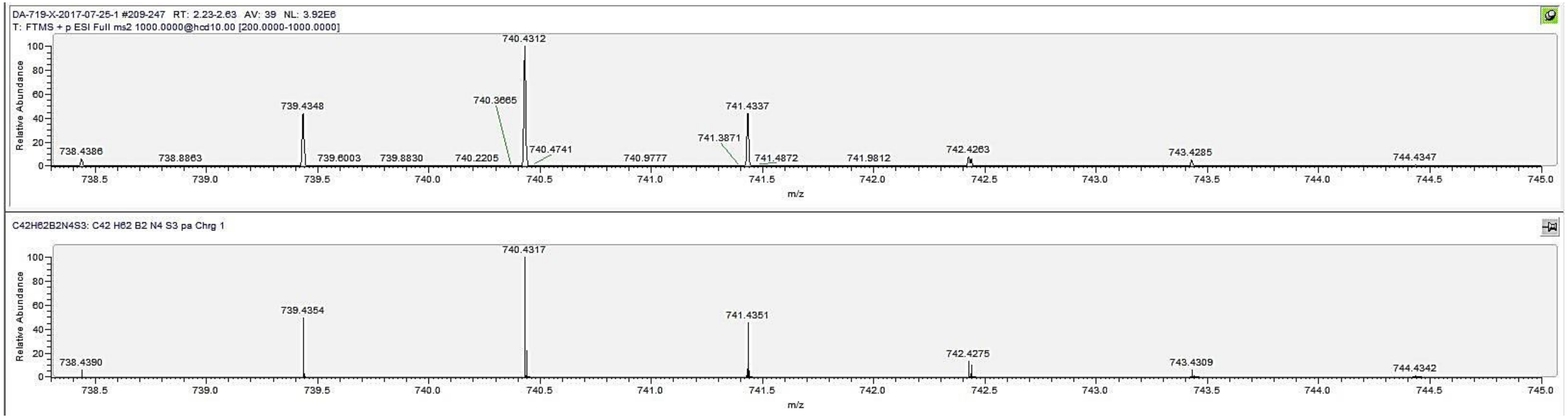

Figure S40. Measured (top) and calculated (bottom) HRMS isotopic distribution pattern for [$C_{42}H_{62}B_2N_4S_3$] = [**8**] (LIFDI-MS).

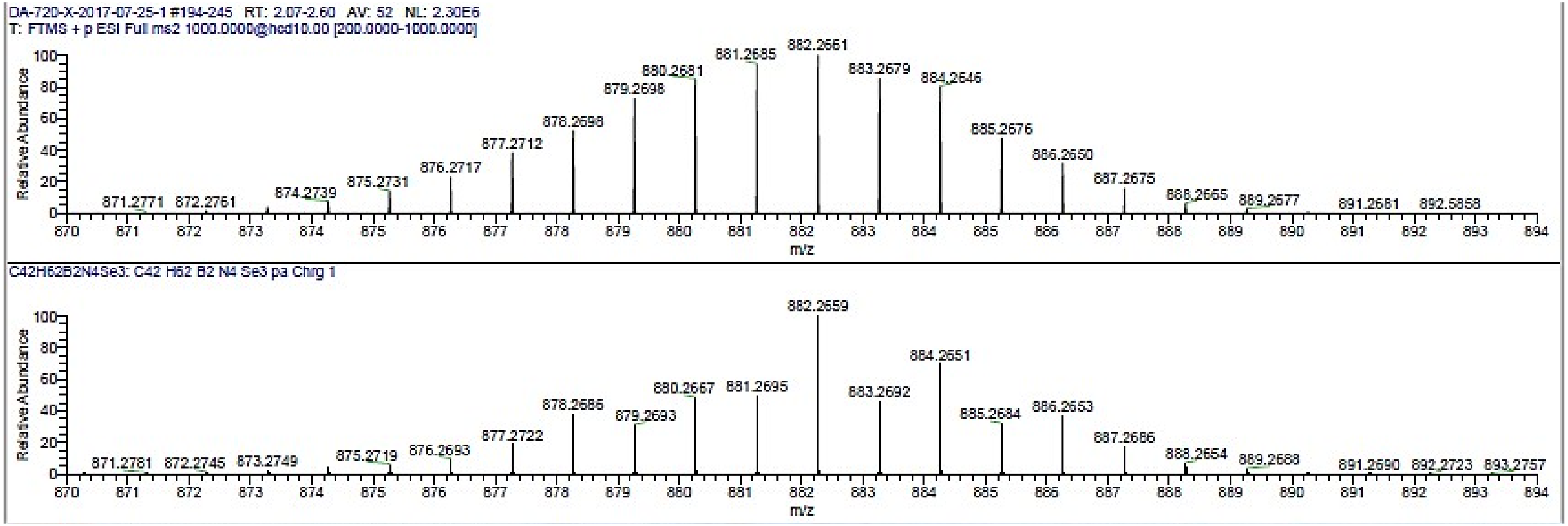

Figure S41. Measured (top) and calculated (bottom) HRMS isotopic distribution pattern for [$C_{42}H_{62}B_2N_4Se_3$] = [**9**] (LIFDI-MS).

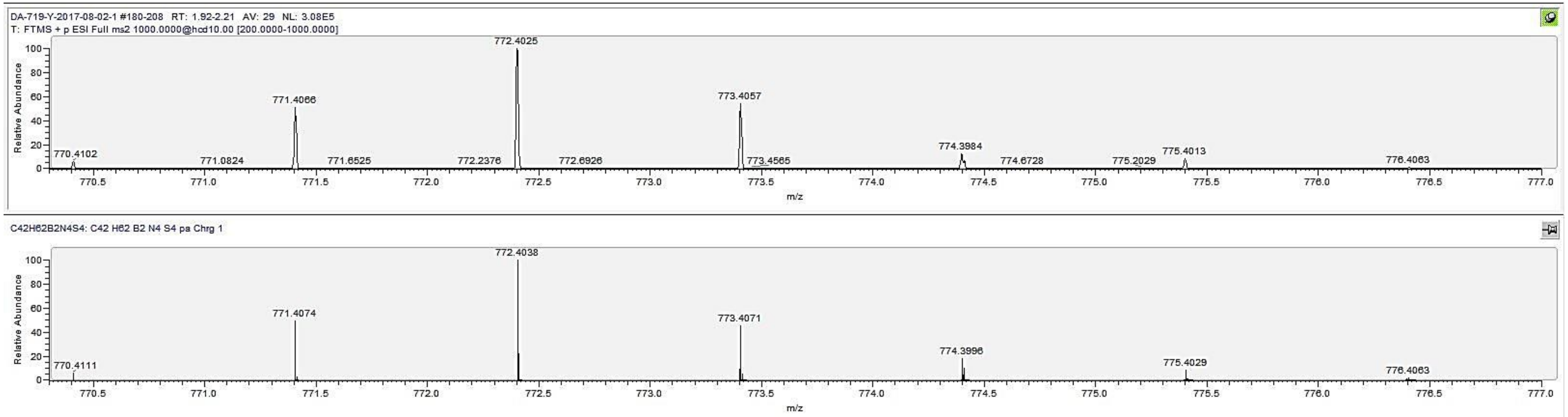

Figure S42. Measured (top) and calculated (bottom) HRMS isotopic distribution pattern for [$C_{42}H_{62}B_2N_4S_4$] [**10**] (LIFDI-MS).

UV-vis spectroscopy

UV-vis spectra were recorded on a JASCO V-660 UV/vis spectrometer in THF at 25 °C.

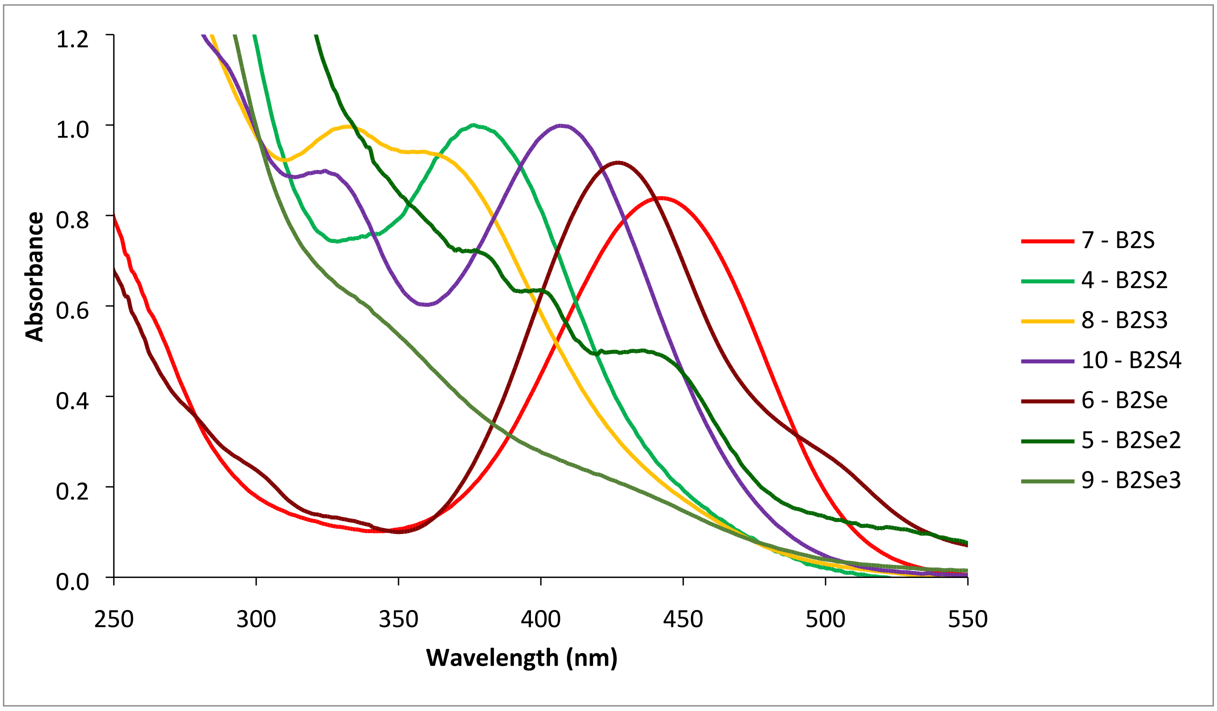

Figure S43. UV-vis spectra of isolated B_2E_n heterocycles: E = S, n = 1 (**7**: λ_{max} = 442), n = 2 (**4**: λ_{max} = 376 nm), 3 (**8**: λ_{max} = 359, 333 nm), 4 (**10**: λ_{max} = 407, 324 nm); E = Se, n = 1 (**6**: λ_{max} = 427 nm), n = 2 (**5**: λ_{max} = 436, 400, 376 nm), 3 (**9**).

X-ray crystallographic details

Crystal data of all compounds were collected on a Bruker X8-APEX II or a Bruker D8 Quest diffractometer with a CCD area detector and *m*-layer mirror monochromated $Mo_{K\alpha}$ radiation. The structures were solved using direct methods, refined with the ShelX software package[3] and expanded using Fourier techniques. All non-hydrogen atoms were refined anisotropically and assigned to idealized positions. Cif files of crystallographic structures have been deposited with the Cambridge Crystallographic Data Centre: CCDC 1582403-1582412.

Refinement details for 1: Two reflections affected by the beamstop were omitted. The cAAC backbone was disordered (C3,C4,C5) in a 76:34 ratio.

Refinement details for 2: The crystal was a very thin, poorly diffracting plate and therefore data were cut at 0.8 Å. The asymmetric unit contains two distinct molecules of the compound, each presenting a twofold disorder in the arrangement of the SeMe ligands, the first in a 63:37 ratio, the second in a 59:41 ratio. Furthermore, the cAAC backbone of the first molecule presents a twofold disorder in the atoms (C6,C7,C8) modelled in a 67:33 ratio. All atom ADPs in disordered parts were restrained using SIMU and DELU. BUMP was added to avoid close H---H interactions caused by disorder.

Refinement details for 6: The entire molecule was twofold disordered in a 88:12 ratio around the central diboraselenirane core in a mirror fashion, with the mirror plane running approximately through the plane containing (N1,Se1,N2). The disordered 2,3-dicyano-2,3-diboraselenirane core (Se1,B1,C21,N2,B2,C51,N4) was freely refined, except for a SAME restraint on the two (B1,C21,N2) parts and SIMU 0.01. The two disordered cAAC ligand frameworks (C1 > C8) and (C31 > C38) were modelled using SAME and SIMU 0.005 restraints. The Dip substituents were kept in common between the two parts of the disorder, only one isopropyl group on each, (C11,C12,C13) and (C41,C42,C43), were also modelled with the twofold disorder to avoid bumping between parts. SAME and SIMU restraints were applied to these two ^{i}Pr groups, as well as a SADI restraint on their attachment to the aryl rings. An additional DFIX 2.2 restraint was added between the hydrogen atom H34B_2 and the isopropyl hydrogen atoms H41_2 and H42B_2 to prevent bumping (a BUMP command was tried but resulted in convergence problems).

Refinement details for 7: After normal integration, the data was refined as a two-component inversion twin with BASF 0.63. An extinction coefficient of 0.003162 was applied. The asymmetric unit contains one toluene molecule disordered over two sites in a 56:44 ratio. The ADPS of these atoms were restrained with SIMU 0.01 and the aromatic rings modelled with AFIX 66.

Refinement details for 10: The data was integrated as a twin with three domains but solved and refined using domain 1 only (ca. 65% of reflections). Subsequent refinement using BASF and

HKLF5 to account for the other two twin domains did not improve R_{int} or R_1 as reflections were too weak beyond 1.00 Å, due to the extreme thinness of the needle-shaped crystals. Multiple attempts to grow larger single crystals of better quality failed but repeated data collections on similarly twinned crystals confirmed the same structural motif. The asymmetric unit contains one molecule of $[(cAAC)B(CN)S_2]_2$ and three molecules of THF. All ADPs were restrained to be similar using SIMU on each of the residues to avoid NPD atoms. The three THF molecules were modelled using SAME. While R_{int}, R_1 and wR_2 are far too high to be of publishable quality, the data provide conclusive proof of connectivity.

Table S1. Details of the X-ray crystallographic diffraction experiments performed on compounds **1-5**.

	**1**	**2**	**3**	**4**	**5**
Formula	$C_{33}H_{41}BN_2S_2$, (C_4H_8O)	$C_{23}H_{37}BN_2Se_2$	$C_{33}H_{41}BN_2Se_2$, (C_4H_8O)	$C_{42}H_{62}B_2N_4S_2$, (C_6H_6)	$C_{42}H_{62}B_2N_4Se_2$, (C_6H_6)
M_W (g·mol^{-1})	612.71	510.27	706.51	786.80	880.64
Colour	colourless	yellow	yellow	yellow	yellow
Crystal system	monoclinic	monoclinic	monoclinic	triclinic	triclinic
Space group	$P\,2_1/n$	$P\,2_1/c$	$P\,2_1/n$	$P\,\bar{1}$	$P\,\bar{1}$
a (Å)	10.672(2)	17.551(6)	10.784(4)	8.8508(9)	8.8289(11)
b (Å)	16.818(4)	15.552(5)	16.852(5)	8.8654(8)	8.8405(16)
c (Å)	18.902(5)	17.729(6)	19.026(6)	14.6186(15)	14.667(3)
α (°)	90	90	90	75.639(3)	85.939(13)
β (°)	91.75(3)	93.183(8)	91.03(3)	86.110(3)	75.916(10)
γ (°)	90	90	90	80.401(3)	79.987(10)
Volume (Å^3)	3390.8(13)	4832(3)	3457.4(19)	1095.24(19)	1093.0(3)
Z	4	8	4	1	1
D_{calc} (mg·m^{-3})	1.200	1.403	1.357	1.193	1.338
μ (mm^{-1})	0.189	3.072	2.170	0.160	1.723
θ_{min} (°)	1.621	2.130	2.417	1.438	1.432
θ_{miax} (°)	26.405	26.370	26.372	27.102	27.876
$F(000)$	1320	2096	1464	426	462
Reflections collected	74082	32869	25978	17332	16496
Independent reflections	6933	9859	7047	4811	5214
R_{int}	0.0648	0.1589	0.2299	0.0769	0.1182
R_1 ($I > 2\sigma$)	0.0352	0.0694	0.0648	0.0605	0.0536
wR_2 (all data)	0.0917	0.2245	0.1440	0.1595	0.1099
Parameters	426	633	396	261	261

Table S2. Details of the X-ray crystallographic diffraction experiments performed on compounds **6-10**.

	**6**	**7**	**8**	**9**	**10 (connectivity)**
Formula	$C_{42}H_{62}B_2N_4Se$	$C_{42}H_{62}B_2N_4S$, (C_7H_8)	$C_{42}H_{62}B_2N_4S_3$, 2 (C_4H_8O)	$C_{42}H_{62}B_2N_4Se_3$, 2 (C_4H_8O)	$C_{42}H_{62}B_2N_4Se_4$, 3 (C_4H_8O)
M_W (g·mol^{-1})	723.53	768.77	884.96	1025.66	989.12
Colour	yellow	orange	yellow	yellow	pale yellow
Crystal system	orthorhombic	monoclinic	monoclinic	monoclinic	orthorhombic
Space group	*P bca*	*P* 2_1/*c*	*C* 2/*c*	*C* 2/*c*	*P bca*
a (Å)	17.8352(15)	18.966(4)	15.945(3)	16.059(3)	16.39(3)
b (Å)	16.7797(16)	13.037(3)	17.530(4)	17.563(3)	17.61(3)
c (Å)	28.365(3)	18.816(5)	17.924(3)	17.928(3)	38.28(7)
α (°)	90	90	90	90	90
β (°)	90	97.143(10)	102.905(4)	103.042(5)	90
γ (°)	90	90	90	90	90
Volume (Å^3)	8488.9(13)	4616.2(18)	4883.6(16)	4926.3(15)	11049(34)
Z	8	4	4	4	8
D_{calc} (mg·m^{-3})	1.117	1.106	1.204	1.383	1.189
μ (mm^{-1})	0.916	0.107	0.195	2.282	0.217
θ_{min} (°)	1.132	1.900	1.751	1.743	1.636
θ_{miax} (°)	27.165	25.678	26.370	27.100	24.713
*F(*000)	3088	1672	1920	2136	4288
Reflections collected	148765	32550	18447	37199	52737
Independent reflections	9396	8748	4997	5443	9390
R_{int}	0.1082	0.1654	0.1820	0.1333	0.9489
R_1 ($I > 2\sigma$)	0.0508	0.0779	0.0738	0.0574	0.2150
wR_2 (all data)	0.1182	0.2174	0.1523	0.1007	0.5370
Parameters	750	565	284	284	621

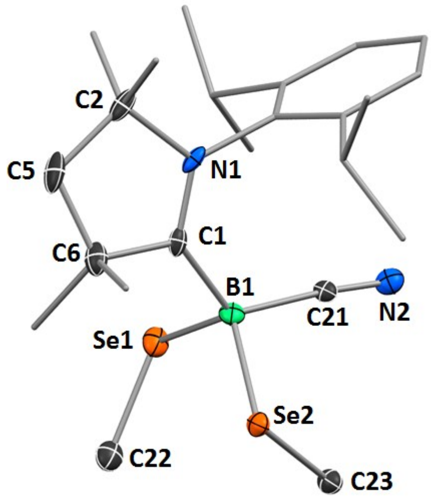

Figure S44. Crystallographically determined solid-state structure of one of the two molecules of **2** found in the asymmetric unit. Atomic displacement ellipsoids depicted at 30% probability level. Hydrogen atoms and atomic displacement ellipsoids of peripheral substituents omitted for clarity.

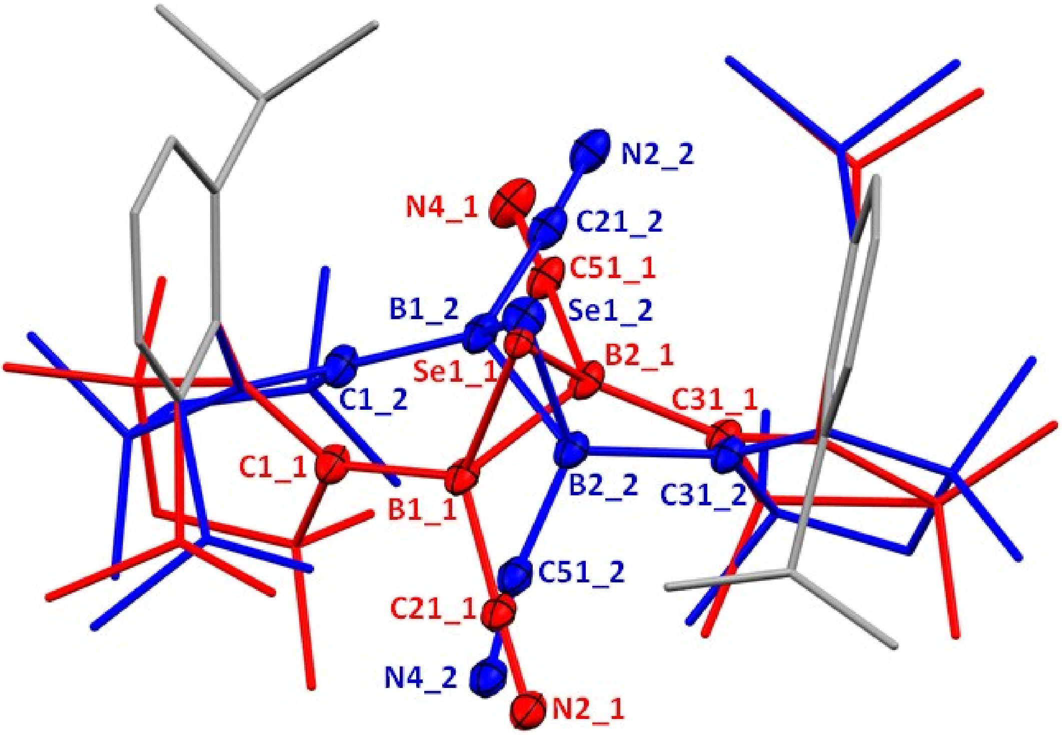

Figure S45. Overlay of the two disordered parts (part 1, red, 88%; part 2, blue, 12%) found in the X-ray crystallographic structure of **6**. The 2,3-dicyano-2,3-diboraselenirane core of each part was freely refined.

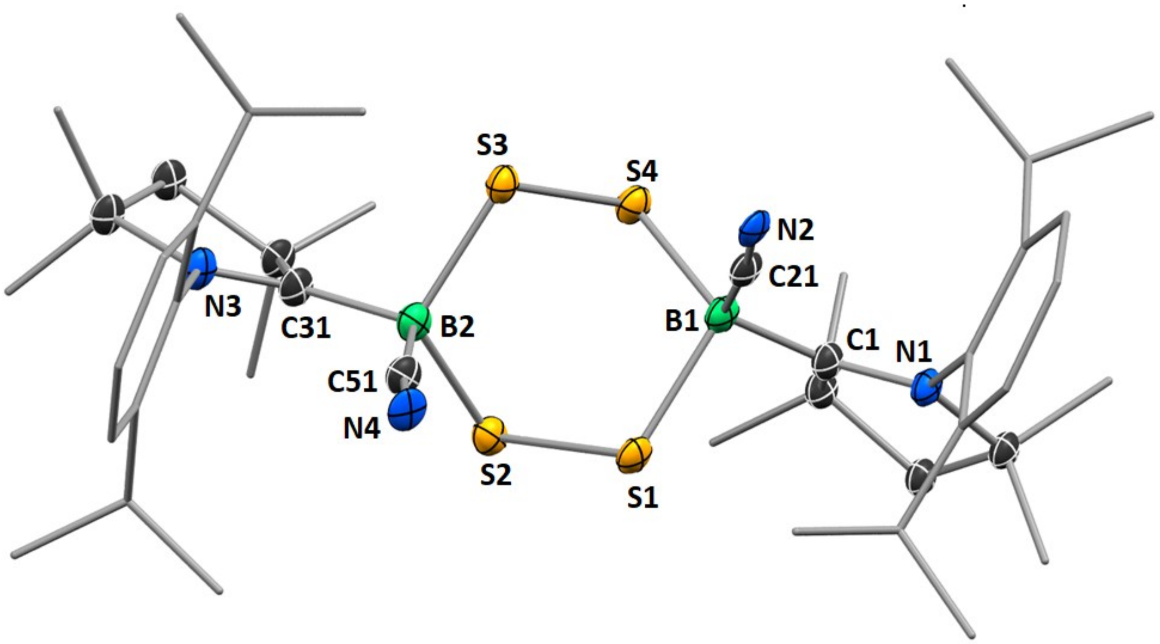

Figure S46. Crystallographically determined solid-state structure of **10** provided as proof of connectivity only. Atomic displacement ellipsoids depicted at 30% probability level. Hydrogen atoms and atomic displacement ellipsoids of peripheral substituents omitted for clarity.

References

[1] M. Arrowsmith, D. Auerhammer, R. Bertermann, H. Braunschweig, G. Bringmann, M. A. Celik, R. D. Dewhurst, M. Finze, M. Grüne, M. Hailmann, T. Hertle and I. Krummenacher, *Angew. Chem. Int. Ed.*, 2016, **55**, 14464.

[2] J. Böhnke, H. Braunschweig, T. Dellermann, W. C. Ewing, T. Kramer, I. Krummenacher and A. Vargas, *Angew. Chem. Int. Ed.*, 2015, **54**, 4469.

[3] G. Sheldrick, *Acta Cryst.*, 2015, **A71**, 3.
